# Supplementary material for: Ki-67 shapes the nucleolus by anchoring chromatin via its amphiphilic properties
Source: EMBO J. 2026 Mar 24;45(9):3156–91. doi: 10.1038/s44318-026-00747-7 (PMC13144362; doi:10.1038/s44318-026-00747-7)
Supplement: Supplementary file 1 — Appendix [file 44318_2026_747_MOESM1_ESM.pdf]

Appendix for

Ki-67 shapes the nucleolus by anchoring chromatin via its amphiphilic properties

Daja Schichler[1,2,†], Yuki Hayashi[1,†], Letitia Fernandez[1,4], Mariam Chupanova[1], Alberto Hernandez-Armendariz[1,2,4,5], Beate Neumann[3], Sara Cuylen-Haering[1,*]

[1] Cell Biology and Biophysics Unit, European Molecular Biology Laboratory (EMBL), Heidelberg, Germany.
[2] Collaboration for Joint PhD Degree between EMBL and Heidelberg University, Faculty of Biosciences, Heidelberg, Germany.
[3]Advanced Light Microscopy Facility, European Molecular Biology Laboratory (EMBL), Heidelberg, Germany.
[4] Present address: Max Planck Institute of Molecular Cell Biology and Genetics, Dresden, Germany
[5] Present address: Cluster of Excellence Physics of Life, TU Dresden, Dresden, Germany
[†] These authors equally contributed

[*] Corresponding author. Email: sara.cuylen-haering@embl.de

Table of Contents

		Page
Appendix Figure S1	Depletion of several candidate proteins that induce nucleolar rounding triggers nucleolar cap formation	2
Appendix Figure S2	Ki-67 depletion or knockout leads to nucleolar rounding	3
Appendix Figure S3	Minimal effects of Ki-67 depletion on nuclear morphology and the number and size of nucleoli	4
Appendix Figure S4	Ki-67 overexpression leads to chromatin incorporation in the nu-cleolar interior	5
Appendix Figure S5	IAA treatment in wild-type Ki-67 cells has no effects on nucleo-lar shape and chromatin enrichment in the nucleolus	6
Appendix Figure S6	Negligible spectral bleed-through from overexpressed Ki-67	7

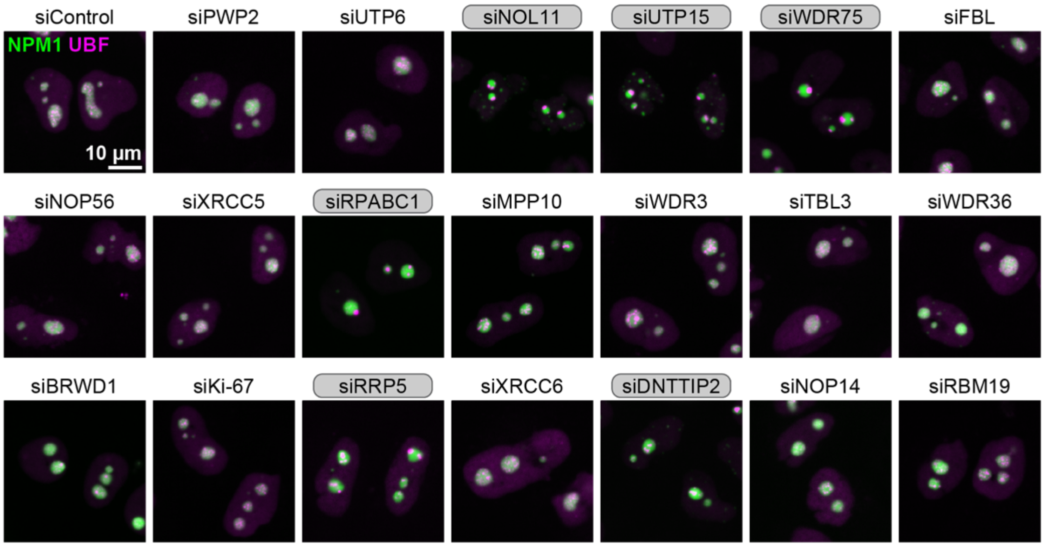

Appendix Figure S1. Depletion of several candidate proteins that induce nucleolar rounding triggers nucleolar cap formation, related to Figure 1.

Nucleolar cap formation following depletion of some top 20 candidates causes nucleolar rounding. HeLa cells endogenously tagged with Halo-UBF and stably expressing SNAP-NPM1 were transfected with a non-targeting control siRNA (siControl) and the siRNAs for the top 20 candidate proteins (Fig. 1). SNAP-NPM1 was labelled with SNAP-SiR (green), and Halo-UBF was labelled with Halo-Tetramethylrhodamine (TMR) (magenta). Nucleolar cap formation was manually annotated (grey boxes) based on the localisation of UBF.

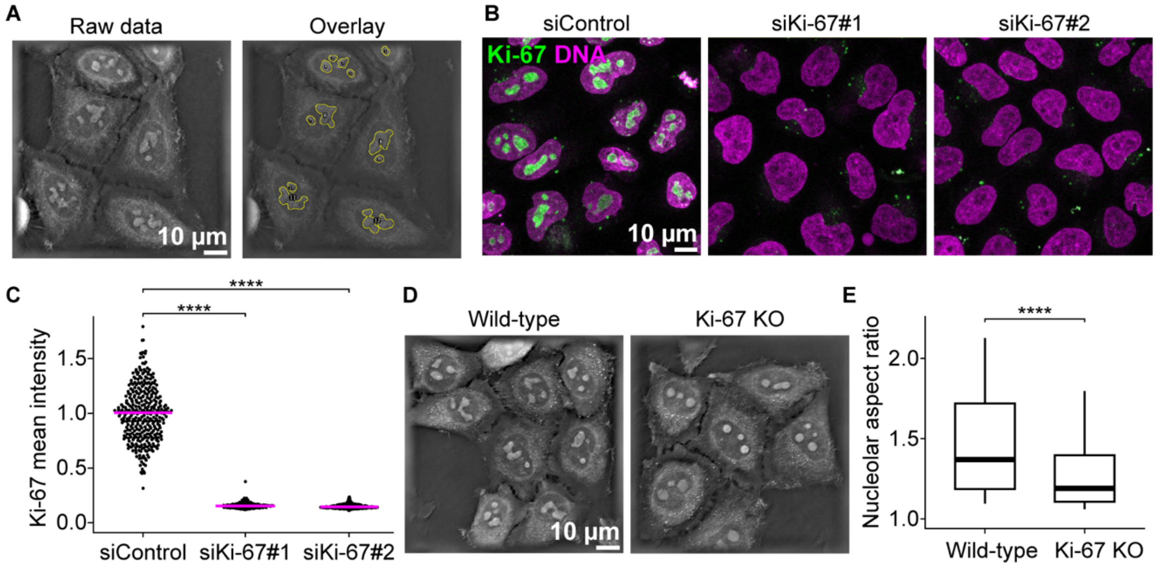

Appendix Figure S2. Ki-67 depletion or knockout leads to nucleolar rounding, related to Figure 1.

(A) Segmentation of nucleoli in holotomographic images. Using a convolutional neural network, nucleoli were segmented from holotomographic images (left), with the segmented regions overlaid in yellow (right).

(B, C) Confirmation of Ki-67 depletion by siRNAs. Cells expressing endogenously tagged EGFP-Ki-67 were transfected with two different siRNAs for Ki-67. DNA was stained with SiR-DNA (B). EGFP-Ki-67 mean nuclear intensity was measured (C). Statistical comparisons were performed against the siControl sample: siKi-67 #1, $p = 2.53 \times 10^{-127}$; siKi-67 #2, $p = 2.04 \times 10^{-173}$.

(D) Label-free holotomographic live imaging of wild-type HeLa cells and Ki-67 knock-out (KO) cells. Single z-slices of a representative example are shown.

(E) Quantification of nucleolar aspect ratio in holotomographic images. The aspect ratio of nucleoli was measured in wild-type and Ki-67 KO cells. Boxplots display the median (centre line), interquartile range (box), and whiskers extending to the 10th and 90th percentiles. Statistical comparison: $p = 8.17 \times 10^{-40}$.

For (C), n = 346 nuclei (siControl), 313 nuclei (siKi-67#1), 357 nuclei (siKi-67#2), 2 biological replicates. Statistical tests were performed with Kruskal–Wallis test followed by Dunn's test,**** P < 0.0001.

For (E), n = 1407 nucleoli (wild-type cells), n = 1405 (Ki-67 KO), 3 biological replicates. Statistical tests were performed with Kolmogorov-Smirnov tests, ****P<0.0001.

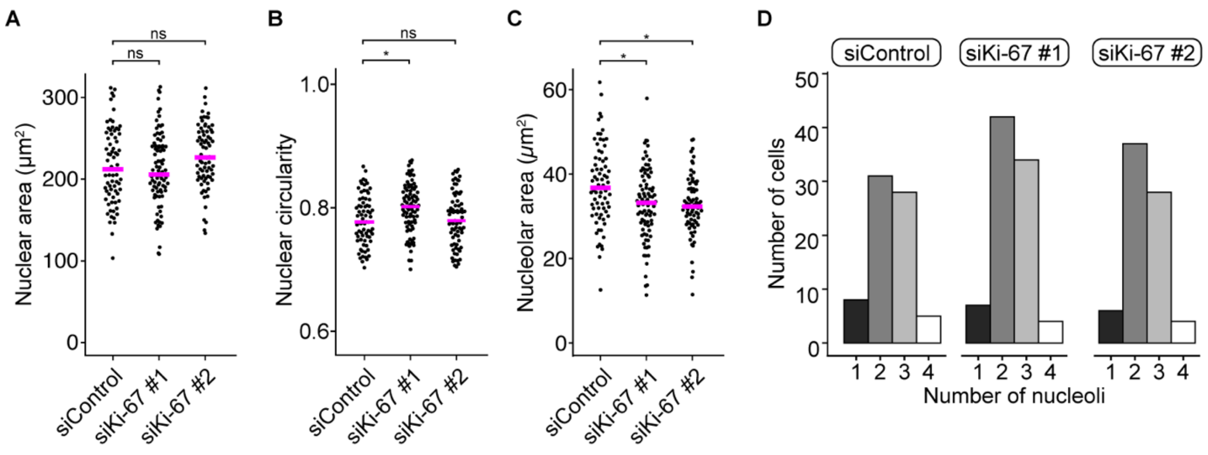

Appendix Figure S3. Minimal effects of Ki-67 depletion on nuclear morphology and the number and size of nucleoli, related to Figure 3.

(A, B) Quantification of nuclear morphologies following Ki-67 depletion. Using data shown in Fig. 3, nuclear area (A) and nuclear aspect ratio (B) were measured. Statistical comparisons were performed against the siControl sample. For (A), siKi-67 #1, $p = 0.649$; siKi-67 #2, $p = 0.103$. For (B), siKi-67 #1, $p = 0.0290$; siKi-67 #2, $p = 0.804$.

(C, D) Quantification of nucleolar area and number following Ki-67 depletion. Using data shown in Fig. 3, total nucleolar area (C) and its number (D) per nucleus were measured. Statistical comparisons in (C) were performed against the siControl sample: siKi-67 #1, $p = 0.0112$; siKi-67 #2, $p = 0.0112$.

For (B–D), n = 72 nuclei (siControl), 87 nuclei (siKi-67#1), 75 nuclei (siKi-67#2), 2 biological replicates.

Statistical tests were performed with Kruskal–Wallis test followed by Dunn's test, ns (not significant) $p > 0.05$, * $p < 0.05$

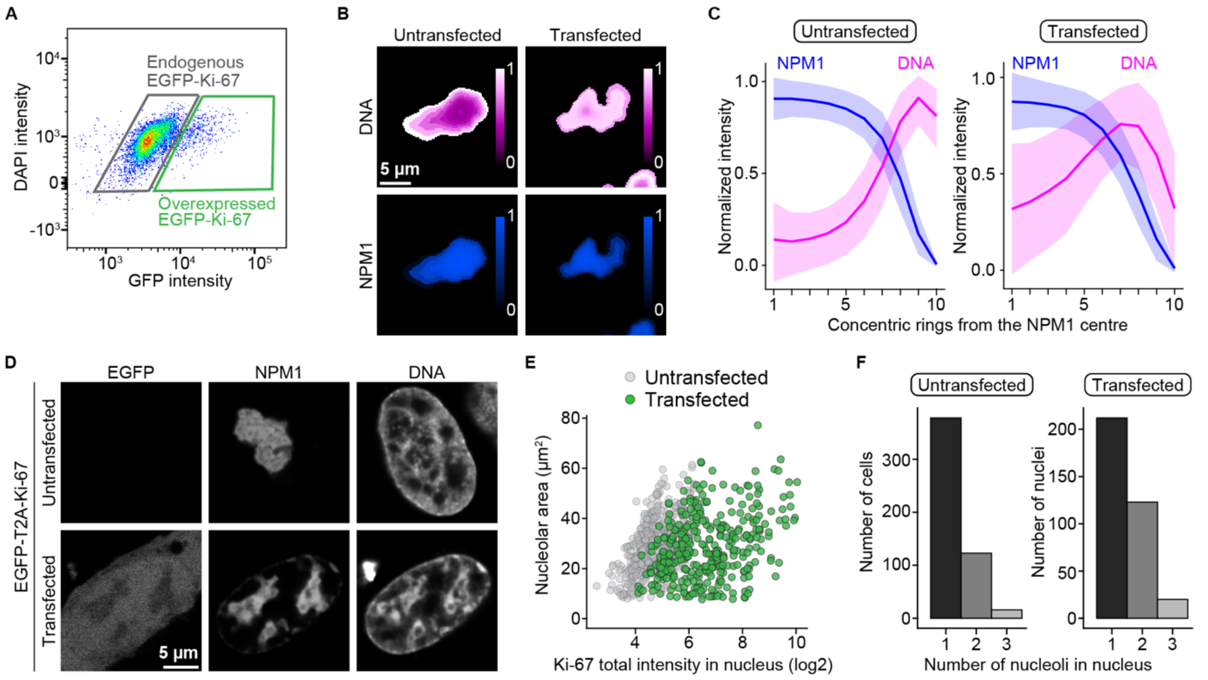

Appendix Figure S4. Ki-67 overexpression leads to chromatin incorporation in the nucleolar interior, related to Figure 4.

(A) Isolation of Ki-67 overexpressing cells by FACS sorting. Cells expressing endogenous EGFP-Ki-67 were used as a reference (grey gate). Cells expressing higher Ki-67 levels than the reference (green gate) were isolated based on the GFP signal intensities by FACS sorting.
(B) Signal distributions of DNA and NPM1 in untransfected cells and Ki-67 overexpressing cells. The nucleolus was segmented into 10 concentric rings from the centre of the nucleolus to the edge of the expanded nucleolus. The colour scale represents the relative intensity.
(C) Quantification of the signal intensity in concentric rings in untransfected cells and Ki-67 overexpressing cells. Mean intensities in each concentric ring were normalised by min-max scaling. Line and shaded areas indicate mean ± SD.
(D) Aberrant nucleolar morphology and chromatin enrichment is caused by Ki-67 itself. HeLa cells expressing SNAP-NPM1 were transfected with EGFP-T2A-Ki-67. Images were acquired following fluorescence labelling of NPM1 and DNA with SNAP-SiR and SPY555-DNA, respectively.
(E) Minimal effects on nucleolar size. The total nucleolar area per nucleus is plotted as a function of the total intensity of EGFP-Ki-67 in the nucleus.
(F) The number of nucleoli per nucleus is shown for untransfected and EGFP-Ki-67–overexpressing cells.
For (C–E), n = 517 nuclei (Untransfected); n = 355 nuclei (Transfected), 2 biological replicates.

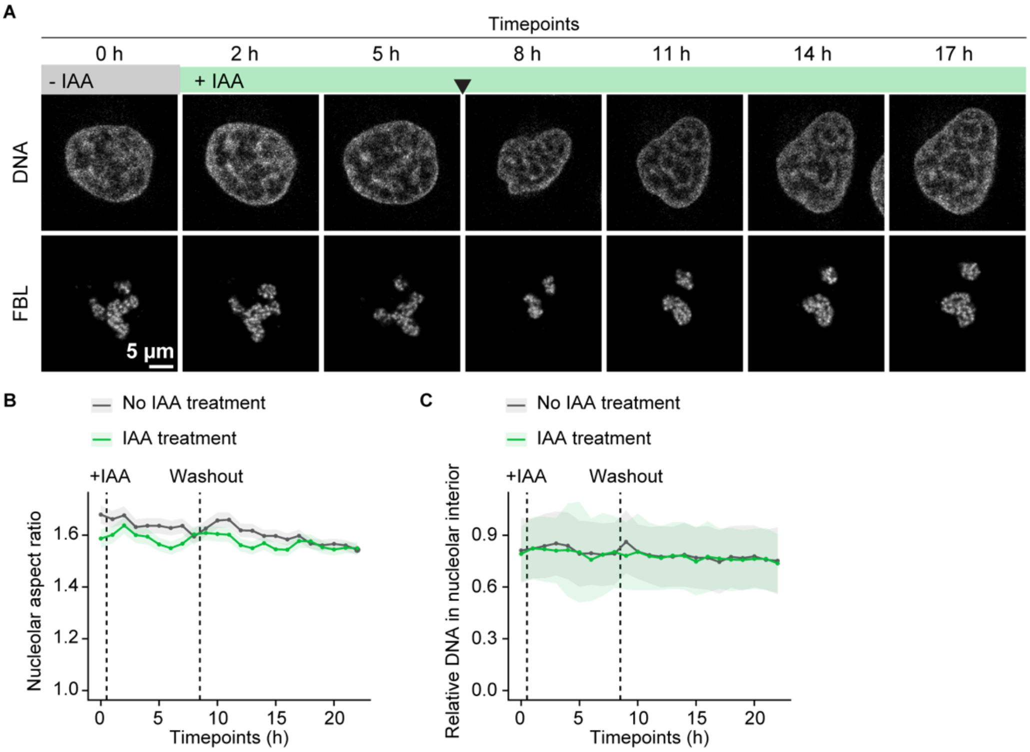

Appendix Figure S5. IAA treatment in wild-type Ki-67 cells has no effects on nucleolar shape and chromatin enrichment in the nucleolus, related to Figure 5.

(A) Treatment with IAA in control cells expressing wild-type Ki-67 and stably overexpressing FBL-TagRFP. IAA was added 0.5 h after the start of time-lapse imaging. DNA was labelled with SiR-DNA. Single z-slice of a representative example quantified in (B) and (C) is shown.

(B, C) Quantification of nucleolar aspect ratio and DNA enrichment within the nucleolus. The aspect ratio of the nucleolus and the relative DNA mean intensity in the nucleolar interior over the nucleus were measured in non-treated cells (grey) and IAA-treated cells (green). Bars and shades indicate mean ± SEM, (B) and mean ± SD (C).

For (B, C), n = 192 to 384 nuclei (No IAA treatment), n = 148 to 363 nuclei (IAA treatment, No washout per time point, 2 biological replicates.

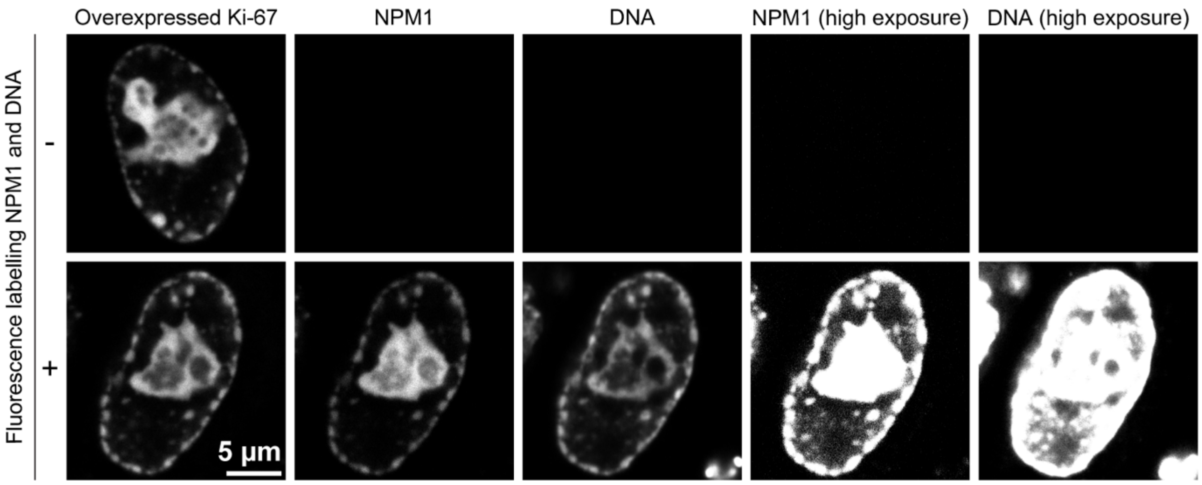

Appendix Figure S6. Negligible spectral bleed-through from overexpressed Ki-67, related to Figure 4.

HeLa cells expressing SNAP-NPM1 were transfected with EGFP-Ki-67. Images were acquired with the same settings with or without fluorescence labelling of SNAP-NPM1 and DNA with SNAP-SiR and SPY555-DNA, respectively.
