# Supplementary material for: Ki-67 shapes the nucleolus by anchoring chromatin via its amphiphilic properties
Source: EMBO J. 2026 Mar 24;45(9):3156–91. doi: 10.1038/s44318-026-00747-7 (PMC13144362; doi:10.1038/s44318-026-00747-7)
Supplement: Supplementary file 4 — Figure Source Data Appendix [file 44318_2026_747_MOESM4_ESM.zip › Appendix_Figure_S4/A/Full_FACS_results.pdf]

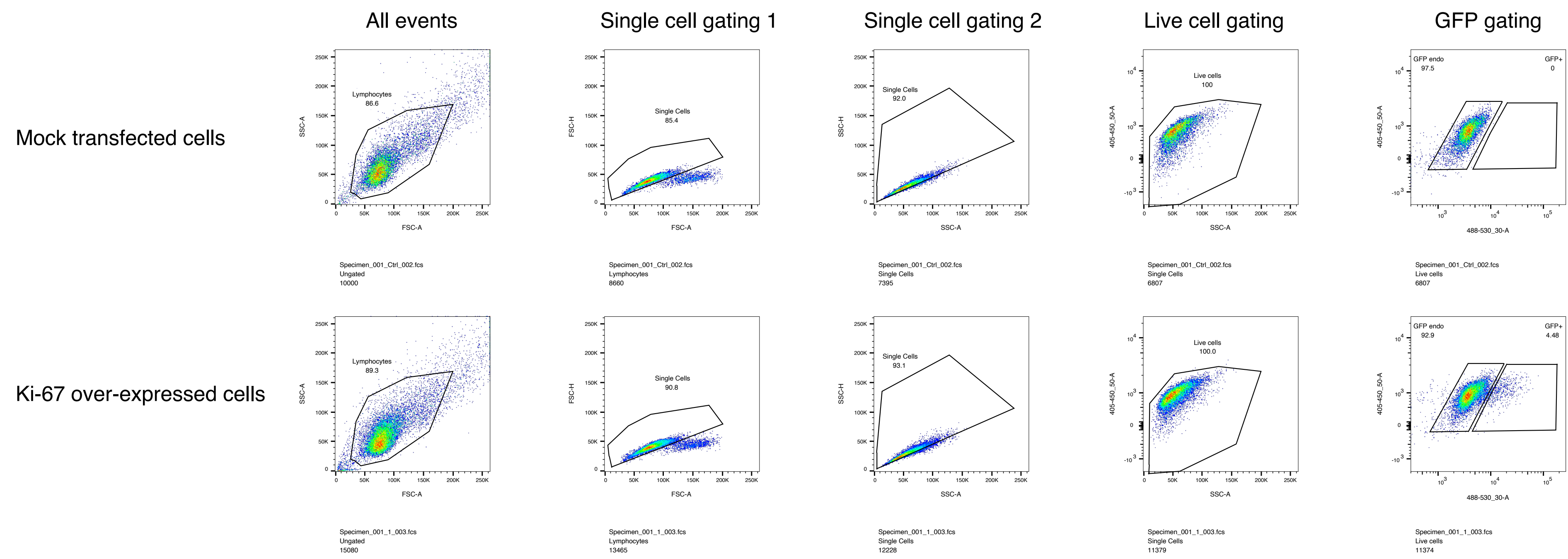
All events
Single cell gating 1
Single cell gating 2
Live cell gating
GFP gating
Mock transfected cells
Lymphocytes
86.6
SSC-A
FSC-A
Specimen_001_Ctrl_002.fcs
Ungated
10000
Single Cells
85.4
FSC-H
FSC-A
Specimen_001_Ctrl_002.fcs
Lymphocytes
8660
Single Cells
92.0
SSC-H
SSC-A
Specimen_001_Ctrl_002.fcs
Single Cells
7395
Live cells
100
405-450_50-A
SSC-A
Specimen_001_Ctrl_002.fcs
Single Cells
6807
GFP endo
97.5
GFP+
0
405-450_50-A
488-530_30-A
Specimen_001_Ctrl_002.fcs
Live cells
6807
Ki-67 over-expressed cells
Lymphocytes
89.3
SSC-A
FSC-A
Specimen_001_1_003.fcs
Ungated
15080
Single Cells
90.8
FSC-H
FSC-A
Specimen_001_1_003.fcs
Lymphocytes
13465
Single Cells
93.1
SSC-H
SSC-A
Specimen_001_1_003.fcs
Single Cells
12228
Live cells
100.0
405-450_50-A
SSC-A
Specimen_001_1_003.fcs
Single Cells
11379
GFP endo
92.9
GFP+
4.48
405-450_50-A
488-530_30-A
Specimen_001_1_003.fcs
Live cells
11374
