# Supplementary figures and images for: Ki-67 shapes the nucleolus by anchoring chromatin via its amphiphilic properties (part 2 of 5)
Source: EMBO J. 2026 Mar 24;45(9):3156–91. doi: 10.1038/s44318-026-00747-7 (PMC13144362; doi:10.1038/s44318-026-00747-7)

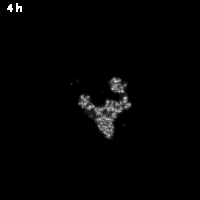

Supplement: Supplementary file 4 — Figure Source Data Appendix [file 44318_2026_747_MOESM4_ESM.zip › Appendix_Figure_S5/A/RGB/cell_23_1_e-0905_W0023_all_channels_stack_Greyscale_hyperstack.tifCh-8_FBL-TagRFP.tif_frame_5.tif]

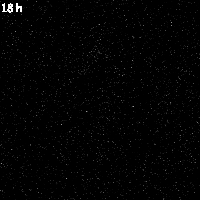

Supplement: Supplementary file 4 — Figure Source Data Appendix [file 44318_2026_747_MOESM4_ESM.zip › Appendix_Figure_S5/A/RGB/cell_23_1_e-0905_W0023_all_channels_stack_Greyscale_hyperstack.tifCh-8_EGFP-AID-Ki-67.tif_frame_19.tif]

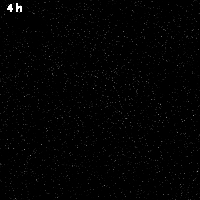

Supplement: Supplementary file 4 — Figure Source Data Appendix [file 44318_2026_747_MOESM4_ESM.zip › Appendix_Figure_S5/A/RGB/cell_23_1_e-0905_W0023_all_channels_stack_Greyscale_hyperstack.tifCh-8_EGFP-AID-Ki-67.tif_frame_5.tif]

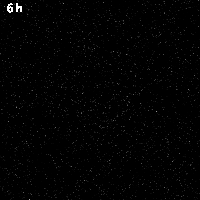

Supplement: Supplementary file 4 — Figure Source Data Appendix [file 44318_2026_747_MOESM4_ESM.zip › Appendix_Figure_S5/A/RGB/cell_23_1_e-0905_W0023_all_channels_stack_Greyscale_hyperstack.tifCh-8_EGFP-AID-Ki-67.tif_frame_7.tif]

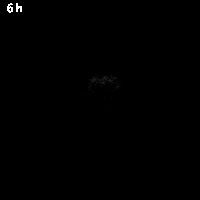

Supplement: Supplementary file 4 — Figure Source Data Appendix [file 44318_2026_747_MOESM4_ESM.zip › Appendix_Figure_S5/A/RGB/cell_23_1_e-0905_W0023_all_channels_stack_Greyscale_hyperstack.tifCh-8_FBL-TagRFP.tif_frame_7.tif]

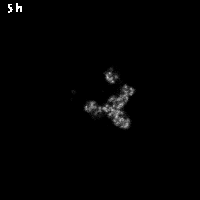

Supplement: Supplementary file 4 — Figure Source Data Appendix [file 44318_2026_747_MOESM4_ESM.zip › Appendix_Figure_S5/A/RGB/cell_23_1_e-0905_W0023_all_channels_stack_Greyscale_hyperstack.tifCh-8_FBL-TagRFP.tif_frame_6.tif]

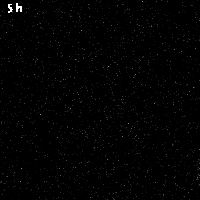

Supplement: Supplementary file 4 — Figure Source Data Appendix [file 44318_2026_747_MOESM4_ESM.zip › Appendix_Figure_S5/A/RGB/cell_23_1_e-0905_W0023_all_channels_stack_Greyscale_hyperstack.tifCh-8_EGFP-AID-Ki-67.tif_frame_6.tif]

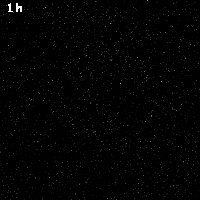

Supplement: Supplementary file 4 — Figure Source Data Appendix [file 44318_2026_747_MOESM4_ESM.zip › Appendix_Figure_S5/A/RGB/cell_23_1_e-0905_W0023_all_channels_stack_Greyscale_hyperstack.tifCh-8_EGFP-AID-Ki-67.tif_frame_2.tif]

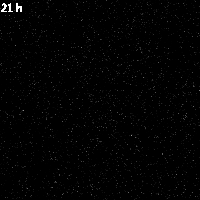

Supplement: Supplementary file 4 — Figure Source Data Appendix [file 44318_2026_747_MOESM4_ESM.zip › Appendix_Figure_S5/A/RGB/cell_23_1_e-0905_W0023_all_channels_stack_Greyscale_hyperstack.tifCh-8_EGFP-AID-Ki-67.tif_frame_22.tif]

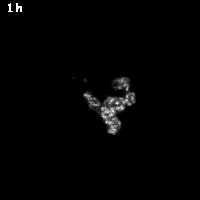

Supplement: Supplementary file 4 — Figure Source Data Appendix [file 44318_2026_747_MOESM4_ESM.zip › Appendix_Figure_S5/A/RGB/cell_23_1_e-0905_W0023_all_channels_stack_Greyscale_hyperstack.tifCh-8_FBL-TagRFP.tif_frame_2.tif]

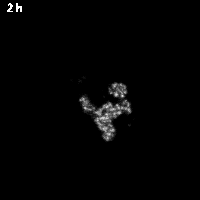

Supplement: Supplementary file 4 — Figure Source Data Appendix [file 44318_2026_747_MOESM4_ESM.zip › Appendix_Figure_S5/A/RGB/cell_23_1_e-0905_W0023_all_channels_stack_Greyscale_hyperstack.tifCh-8_FBL-TagRFP.tif_frame_3.tif]

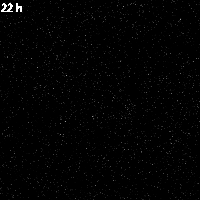

Supplement: Supplementary file 4 — Figure Source Data Appendix [file 44318_2026_747_MOESM4_ESM.zip › Appendix_Figure_S5/A/RGB/cell_23_1_e-0905_W0023_all_channels_stack_Greyscale_hyperstack.tifCh-8_EGFP-AID-Ki-67.tif_frame_23.tif]

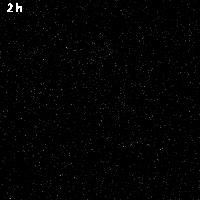

Supplement: Supplementary file 4 — Figure Source Data Appendix [file 44318_2026_747_MOESM4_ESM.zip › Appendix_Figure_S5/A/RGB/cell_23_1_e-0905_W0023_all_channels_stack_Greyscale_hyperstack.tifCh-8_EGFP-AID-Ki-67.tif_frame_3.tif]

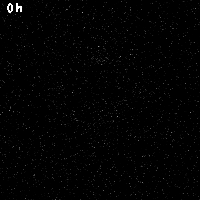

Supplement: Supplementary file 4 — Figure Source Data Appendix [file 44318_2026_747_MOESM4_ESM.zip › Appendix_Figure_S5/A/RGB/cell_23_1_e-0905_W0023_all_channels_stack_Greyscale_hyperstack.tifCh-8_EGFP-AID-Ki-67.tif_frame_1.tif]

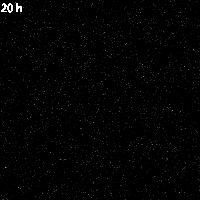

Supplement: Supplementary file 4 — Figure Source Data Appendix [file 44318_2026_747_MOESM4_ESM.zip › Appendix_Figure_S5/A/RGB/cell_23_1_e-0905_W0023_all_channels_stack_Greyscale_hyperstack.tifCh-8_EGFP-AID-Ki-67.tif_frame_21.tif]

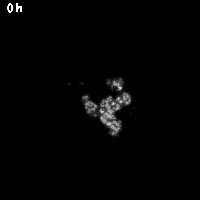

Supplement: Supplementary file 4 — Figure Source Data Appendix [file 44318_2026_747_MOESM4_ESM.zip › Appendix_Figure_S5/A/RGB/cell_23_1_e-0905_W0023_all_channels_stack_Greyscale_hyperstack.tifCh-8_FBL-TagRFP.tif_frame_1.tif]

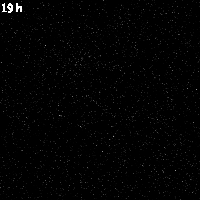

Supplement: Supplementary file 4 — Figure Source Data Appendix [file 44318_2026_747_MOESM4_ESM.zip › Appendix_Figure_S5/A/RGB/cell_23_1_e-0905_W0023_all_channels_stack_Greyscale_hyperstack.tifCh-8_EGFP-AID-Ki-67.tif_frame_20.tif]

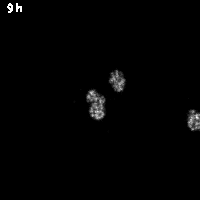

Supplement: Supplementary file 4 — Figure Source Data Appendix [file 44318_2026_747_MOESM4_ESM.zip › Appendix_Figure_S5/A/RGB/cell_23_1_e-0905_W0023_all_channels_stack_Greyscale_hyperstack.tifCh-8_FBL-TagRFP.tif_frame_10.tif]

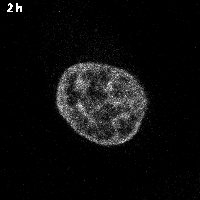

Supplement: Supplementary file 4 — Figure Source Data Appendix [file 44318_2026_747_MOESM4_ESM.zip › Appendix_Figure_S5/A/RGB/cell_23_1_e-0905_W0023_all_channels_stack_Greyscale_hyperstack.tifCh-6_SiR-DNA.tif_frame_3.tif]

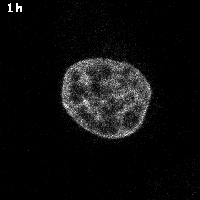

Supplement: Supplementary file 4 — Figure Source Data Appendix [file 44318_2026_747_MOESM4_ESM.zip › Appendix_Figure_S5/A/RGB/cell_23_1_e-0905_W0023_all_channels_stack_Greyscale_hyperstack.tifCh-6_SiR-DNA.tif_frame_2.tif]

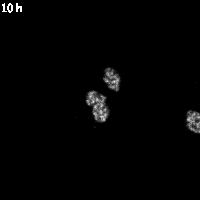

Supplement: Supplementary file 4 — Figure Source Data Appendix [file 44318_2026_747_MOESM4_ESM.zip › Appendix_Figure_S5/A/RGB/cell_23_1_e-0905_W0023_all_channels_stack_Greyscale_hyperstack.tifCh-8_FBL-TagRFP.tif_frame_11.tif]

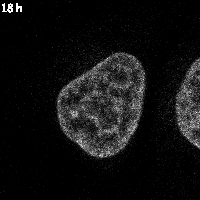

Supplement: Supplementary file 4 — Figure Source Data Appendix [file 44318_2026_747_MOESM4_ESM.zip › Appendix_Figure_S5/A/RGB/cell_23_1_e-0905_W0023_all_channels_stack_Greyscale_hyperstack.tifCh-6_SiR-DNA.tif_frame_19.tif]

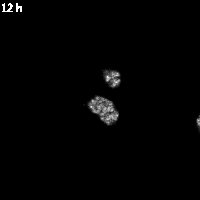

Supplement: Supplementary file 4 — Figure Source Data Appendix [file 44318_2026_747_MOESM4_ESM.zip › Appendix_Figure_S5/A/RGB/cell_23_1_e-0905_W0023_all_channels_stack_Greyscale_hyperstack.tifCh-8_FBL-TagRFP.tif_frame_13.tif]

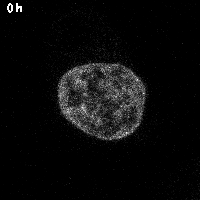

Supplement: Supplementary file 4 — Figure Source Data Appendix [file 44318_2026_747_MOESM4_ESM.zip › Appendix_Figure_S5/A/RGB/cell_23_1_e-0905_W0023_all_channels_stack_Greyscale_hyperstack.tifCh-6_SiR-DNA.tif_frame_1.tif]

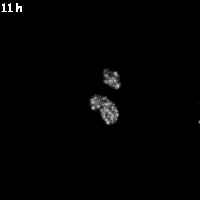

Supplement: Supplementary file 4 — Figure Source Data Appendix [file 44318_2026_747_MOESM4_ESM.zip › Appendix_Figure_S5/A/RGB/cell_23_1_e-0905_W0023_all_channels_stack_Greyscale_hyperstack.tifCh-8_FBL-TagRFP.tif_frame_12.tif]

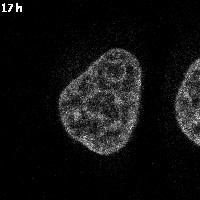

Supplement: Supplementary file 4 — Figure Source Data Appendix [file 44318_2026_747_MOESM4_ESM.zip › Appendix_Figure_S5/A/RGB/cell_23_1_e-0905_W0023_all_channels_stack_Greyscale_hyperstack.tifCh-6_SiR-DNA.tif_frame_18.tif]

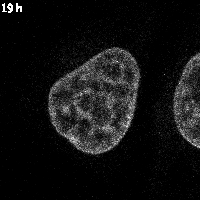

Supplement: Supplementary file 4 — Figure Source Data Appendix [file 44318_2026_747_MOESM4_ESM.zip › Appendix_Figure_S5/A/RGB/cell_23_1_e-0905_W0023_all_channels_stack_Greyscale_hyperstack.tifCh-6_SiR-DNA.tif_frame_20.tif]

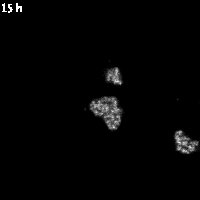

Supplement: Supplementary file 4 — Figure Source Data Appendix [file 44318_2026_747_MOESM4_ESM.zip › Appendix_Figure_S5/A/RGB/cell_23_1_e-0905_W0023_all_channels_stack_Greyscale_hyperstack.tifCh-8_FBL-TagRFP.tif_frame_16.tif]

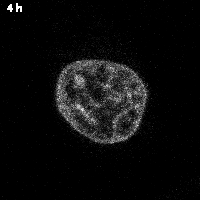

Supplement: Supplementary file 4 — Figure Source Data Appendix [file 44318_2026_747_MOESM4_ESM.zip › Appendix_Figure_S5/A/RGB/cell_23_1_e-0905_W0023_all_channels_stack_Greyscale_hyperstack.tifCh-6_SiR-DNA.tif_frame_5.tif]

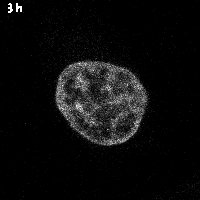

Supplement: Supplementary file 4 — Figure Source Data Appendix [file 44318_2026_747_MOESM4_ESM.zip › Appendix_Figure_S5/A/RGB/cell_23_1_e-0905_W0023_all_channels_stack_Greyscale_hyperstack.tifCh-6_SiR-DNA.tif_frame_4.tif]

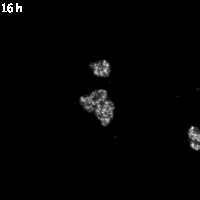

Supplement: Supplementary file 4 — Figure Source Data Appendix [file 44318_2026_747_MOESM4_ESM.zip › Appendix_Figure_S5/A/RGB/cell_23_1_e-0905_W0023_all_channels_stack_Greyscale_hyperstack.tifCh-8_FBL-TagRFP.tif_frame_17.tif]

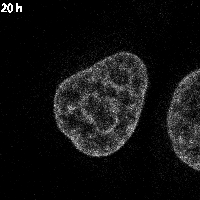

Supplement: Supplementary file 4 — Figure Source Data Appendix [file 44318_2026_747_MOESM4_ESM.zip › Appendix_Figure_S5/A/RGB/cell_23_1_e-0905_W0023_all_channels_stack_Greyscale_hyperstack.tifCh-6_SiR-DNA.tif_frame_21.tif]

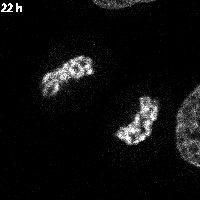

Supplement: Supplementary file 4 — Figure Source Data Appendix [file 44318_2026_747_MOESM4_ESM.zip › Appendix_Figure_S5/A/RGB/cell_23_1_e-0905_W0023_all_channels_stack_Greyscale_hyperstack.tifCh-6_SiR-DNA.tif_frame_23.tif]

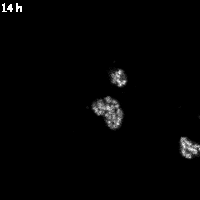

Supplement: Supplementary file 4 — Figure Source Data Appendix [file 44318_2026_747_MOESM4_ESM.zip › Appendix_Figure_S5/A/RGB/cell_23_1_e-0905_W0023_all_channels_stack_Greyscale_hyperstack.tifCh-8_FBL-TagRFP.tif_frame_15.tif]

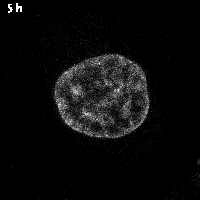

Supplement: Supplementary file 4 — Figure Source Data Appendix [file 44318_2026_747_MOESM4_ESM.zip › Appendix_Figure_S5/A/RGB/cell_23_1_e-0905_W0023_all_channels_stack_Greyscale_hyperstack.tifCh-6_SiR-DNA.tif_frame_6.tif]

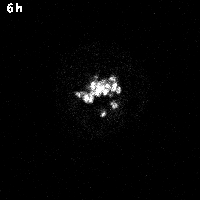

Supplement: Supplementary file 4 — Figure Source Data Appendix [file 44318_2026_747_MOESM4_ESM.zip › Appendix_Figure_S5/A/RGB/cell_23_1_e-0905_W0023_all_channels_stack_Greyscale_hyperstack.tifCh-6_SiR-DNA.tif_frame_7.tif]

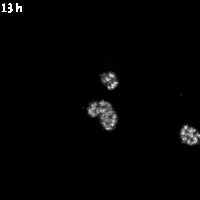

Supplement: Supplementary file 4 — Figure Source Data Appendix [file 44318_2026_747_MOESM4_ESM.zip › Appendix_Figure_S5/A/RGB/cell_23_1_e-0905_W0023_all_channels_stack_Greyscale_hyperstack.tifCh-8_FBL-TagRFP.tif_frame_14.tif]

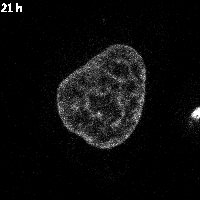

Supplement: Supplementary file 4 — Figure Source Data Appendix [file 44318_2026_747_MOESM4_ESM.zip › Appendix_Figure_S5/A/RGB/cell_23_1_e-0905_W0023_all_channels_stack_Greyscale_hyperstack.tifCh-6_SiR-DNA.tif_frame_22.tif]

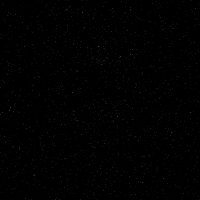

Supplement: Supplementary file 4 — Figure Source Data Appendix [file 44318_2026_747_MOESM4_ESM.zip › Appendix_Figure_S5/A/raw/cell_23_1_e-0905_W0023_all_channels_stack_Greyscale_hyperstack.tifCh-8_EGFP-AID-Ki-67.tif]

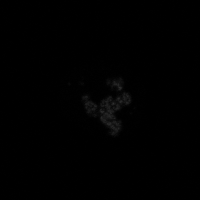

Supplement: Supplementary file 4 — Figure Source Data Appendix [file 44318_2026_747_MOESM4_ESM.zip › Appendix_Figure_S5/A/raw/cell_23_1_e-0905_W0023_all_channels_stack_Greyscale_hyperstack.tifCh-8_FBL-TagRFP.tif]

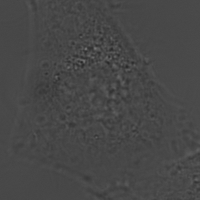

Supplement: Supplementary file 4 — Figure Source Data Appendix [file 44318_2026_747_MOESM4_ESM.zip › Appendix_Figure_S5/A/raw/cell_23_1_e-0905_W0023_all_channels_stack_Greyscale_hyperstack.tifCh-7_TM.tif]

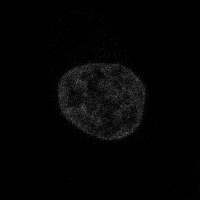

Supplement: Supplementary file 4 — Figure Source Data Appendix [file 44318_2026_747_MOESM4_ESM.zip › Appendix_Figure_S5/A/raw/cell_23_1_e-0905_W0023_all_channels_stack_Greyscale_hyperstack.tifCh-6_SiR-DNA.tif]

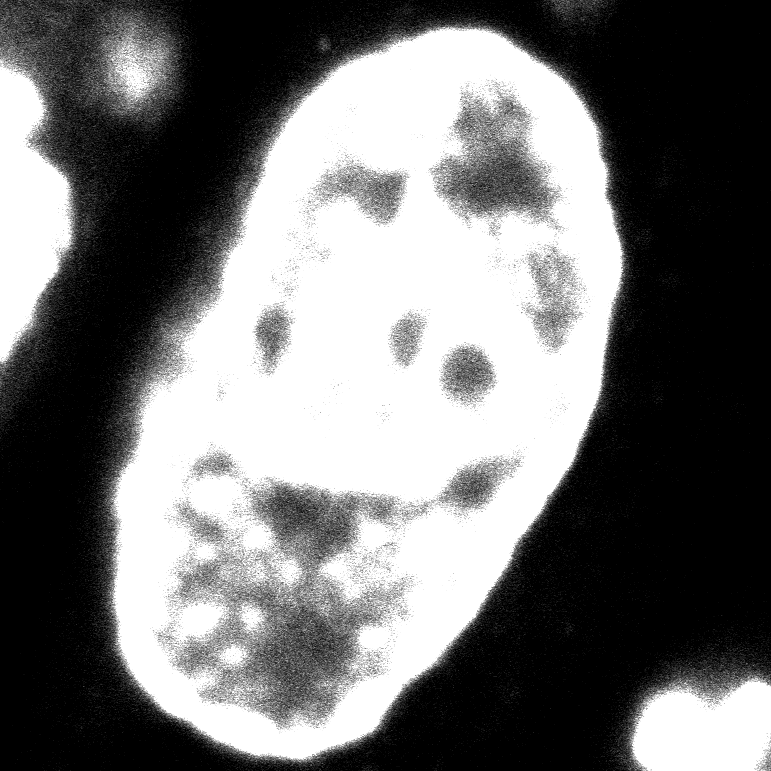

Supplement: Supplementary file 4 — Figure Source Data Appendix [file 44318_2026_747_MOESM4_ESM.zip › Appendix_Figure_S6/02_gray_scale_same_brightness_for_SPY555-DNA_comparison/e1575_p343_SPY-SNAP_zoom8_01-1_ch04_high_exposure.tif]

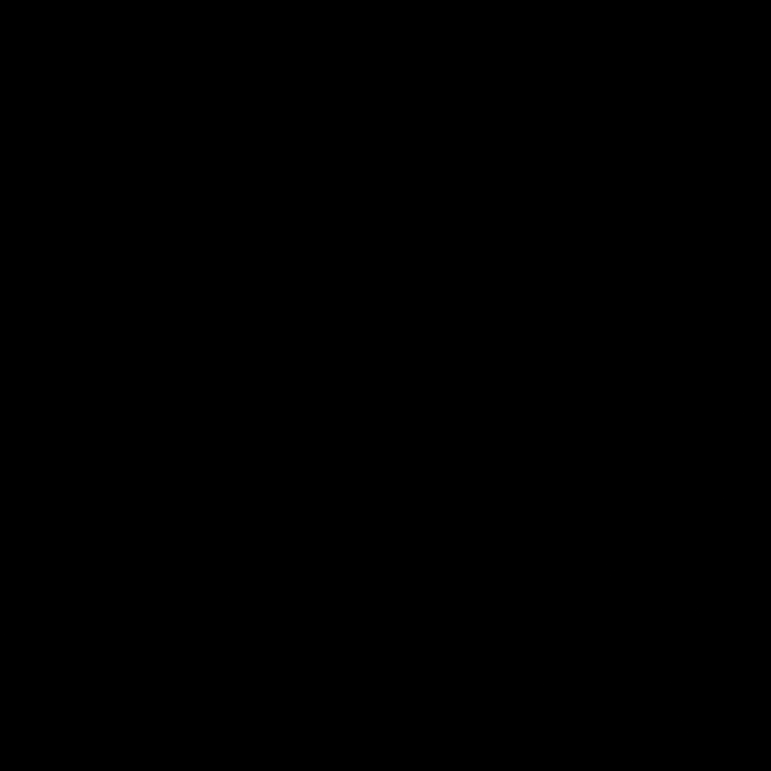

Supplement: Supplementary file 4 — Figure Source Data Appendix [file 44318_2026_747_MOESM4_ESM.zip › Appendix_Figure_S6/02_gray_scale_same_brightness_for_SPY555-DNA_comparison/e1575_p343_NoLabelling_zoom8_03-1_ch04.tif]

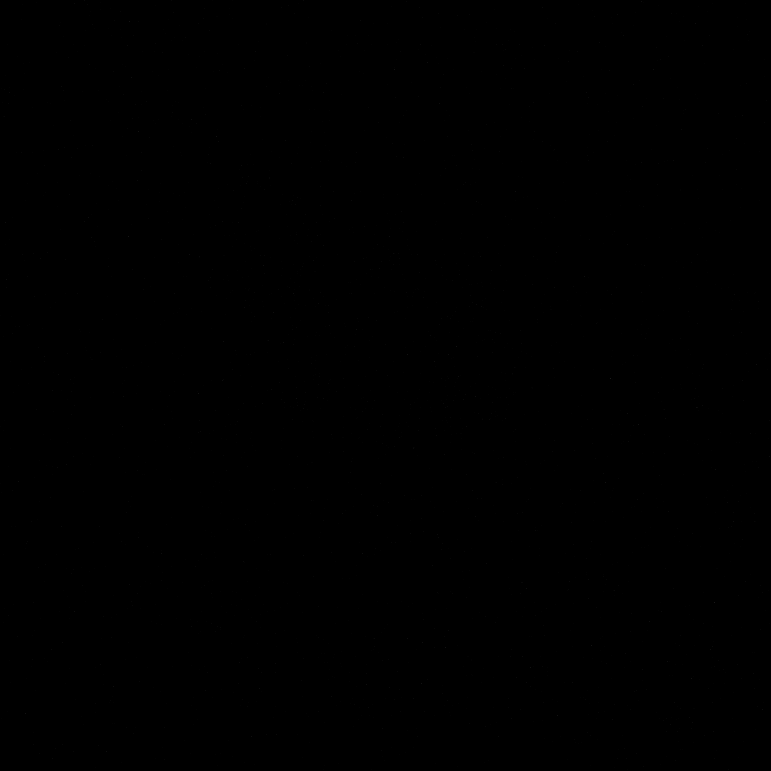

Supplement: Supplementary file 4 — Figure Source Data Appendix [file 44318_2026_747_MOESM4_ESM.zip › Appendix_Figure_S6/02_gray_scale_same_brightness_for_SPY555-DNA_comparison/e1575_p343_NoLabelling_zoom8_03-1_ch02.tif]

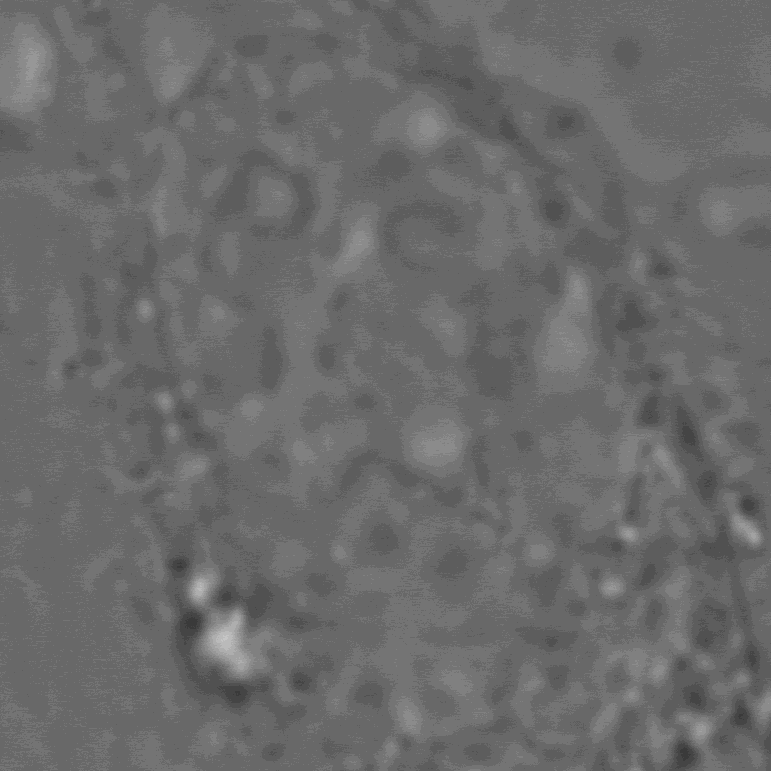

Supplement: Supplementary file 4 — Figure Source Data Appendix [file 44318_2026_747_MOESM4_ESM.zip › Appendix_Figure_S6/02_gray_scale_same_brightness_for_SPY555-DNA_comparison/e1575_p343_NoLabelling_zoom8_03-1_ch03.tif]

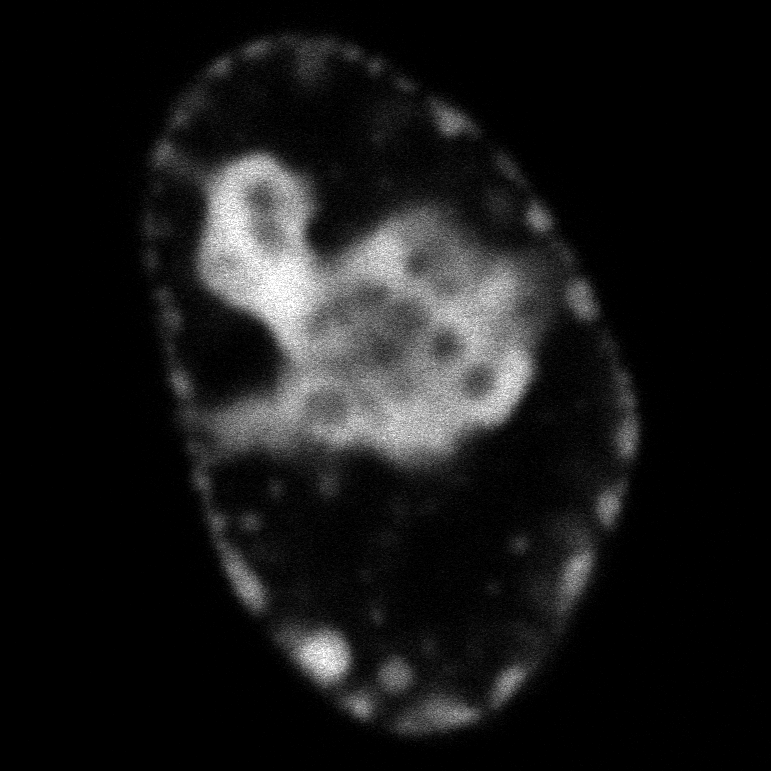

Supplement: Supplementary file 4 — Figure Source Data Appendix [file 44318_2026_747_MOESM4_ESM.zip › Appendix_Figure_S6/02_gray_scale_same_brightness_for_SPY555-DNA_comparison/e1575_p343_NoLabelling_zoom8_03-1_ch01.tif]

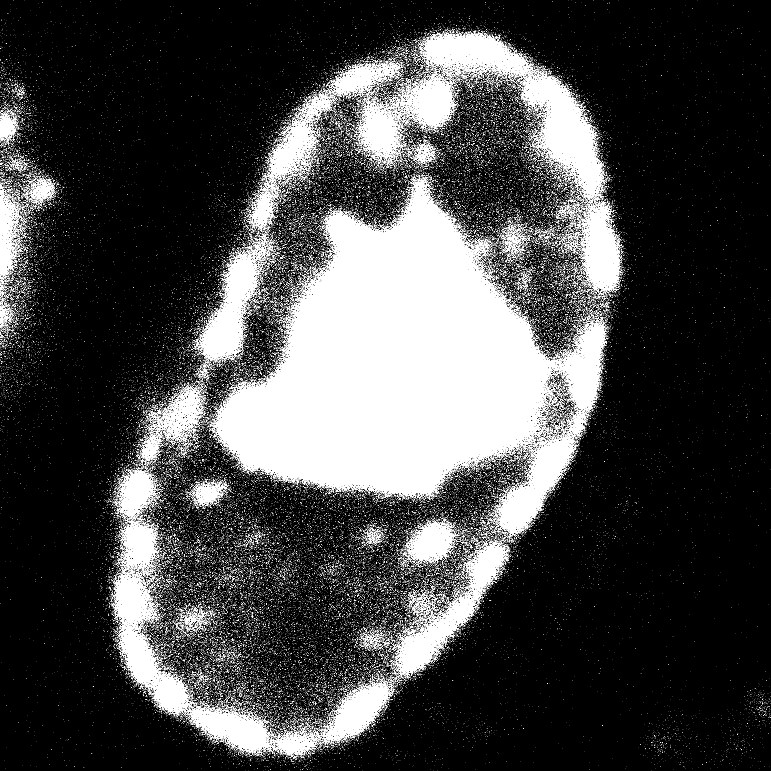

Supplement: Supplementary file 4 — Figure Source Data Appendix [file 44318_2026_747_MOESM4_ESM.zip › Appendix_Figure_S6/02_gray_scale_same_brightness_for_SPY555-DNA_comparison/e1575_p343_SPY-SNAP_zoom8_01-1_ch02_high_exposure.tif]

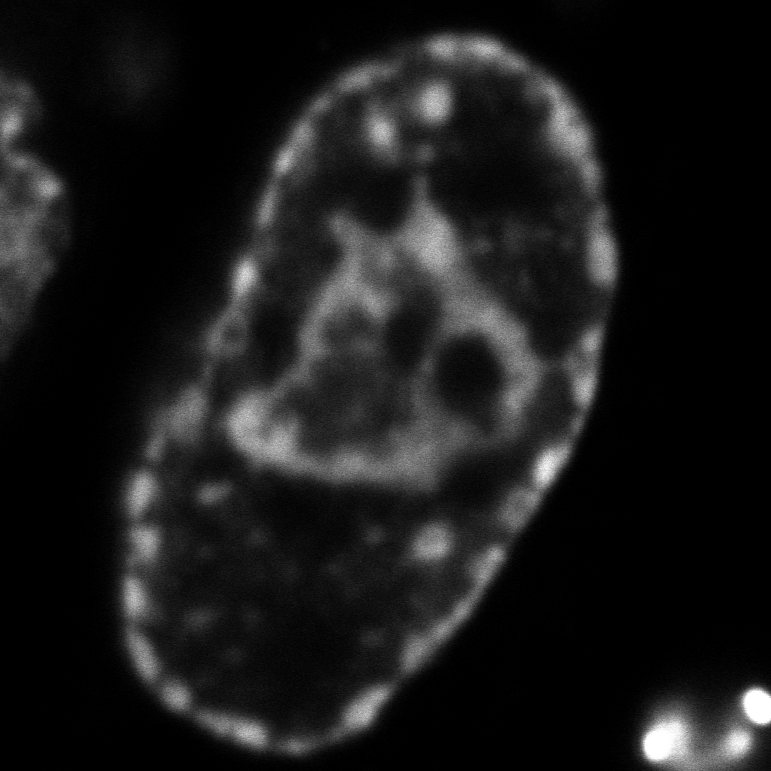

Supplement: Supplementary file 4 — Figure Source Data Appendix [file 44318_2026_747_MOESM4_ESM.zip › Appendix_Figure_S6/02_gray_scale_same_brightness_for_SPY555-DNA_comparison/e1575_p343_SPY-SNAP_zoom8_01-1_ch04.tif]

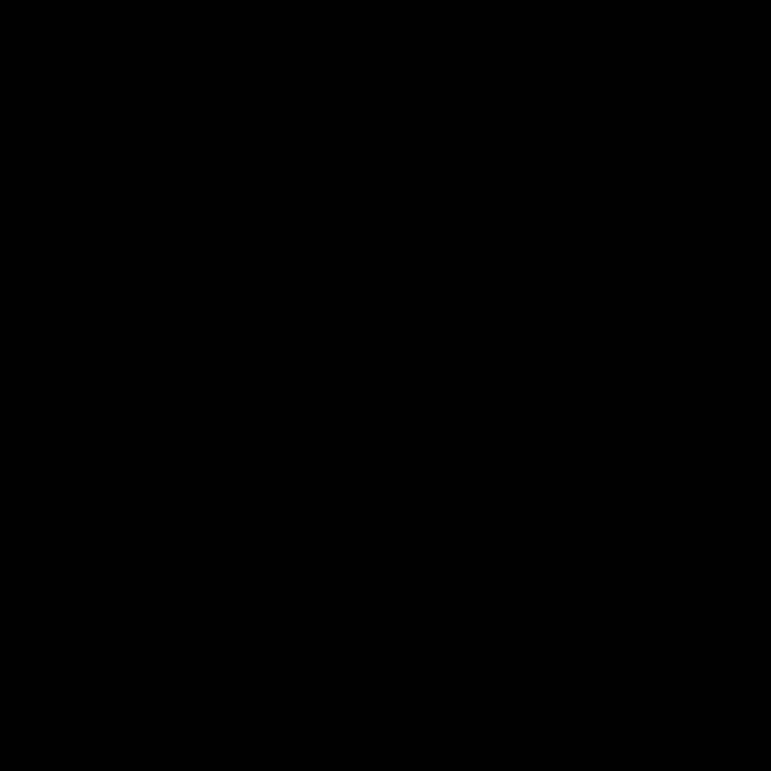

Supplement: Supplementary file 4 — Figure Source Data Appendix [file 44318_2026_747_MOESM4_ESM.zip › Appendix_Figure_S6/02_gray_scale_same_brightness_for_SPY555-DNA_comparison/e1575_p343_NoLabelling_zoom8_03-1_ch04_high_exposure.tif]

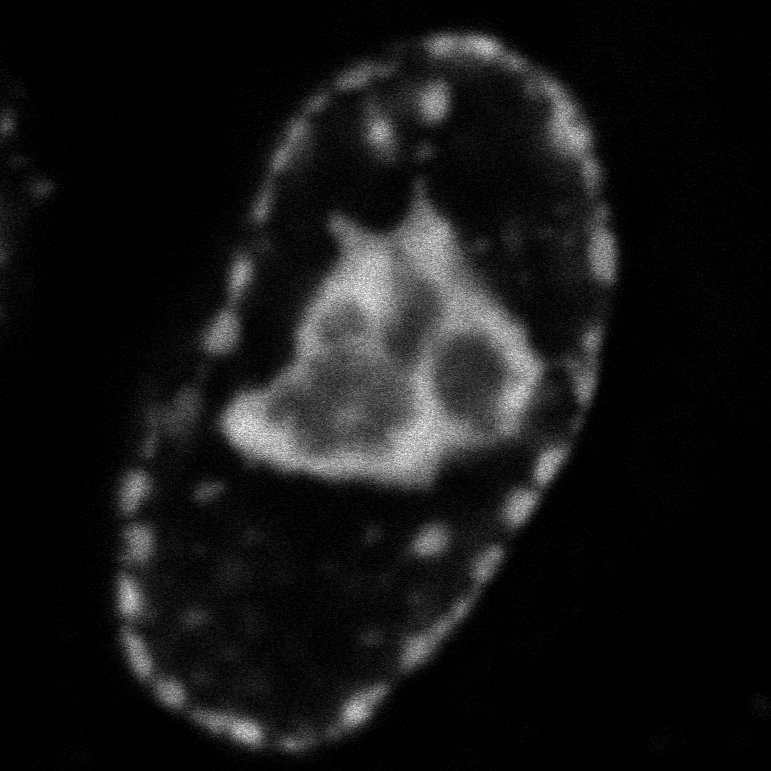

Supplement: Supplementary file 4 — Figure Source Data Appendix [file 44318_2026_747_MOESM4_ESM.zip › Appendix_Figure_S6/02_gray_scale_same_brightness_for_SPY555-DNA_comparison/e1575_p343_SPY-SNAP_zoom8_01-1_ch01.tif]

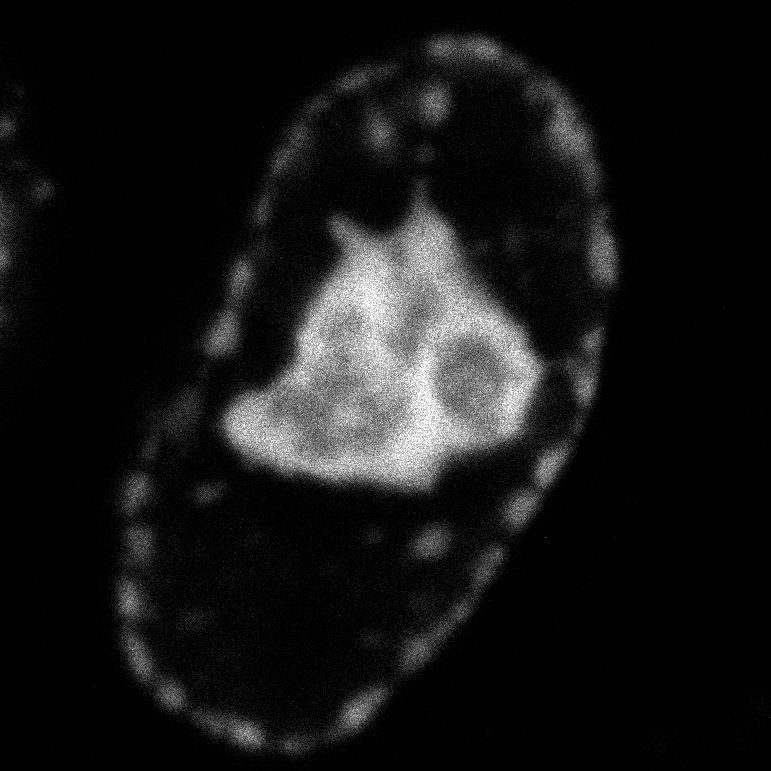

Supplement: Supplementary file 4 — Figure Source Data Appendix [file 44318_2026_747_MOESM4_ESM.zip › Appendix_Figure_S6/02_gray_scale_same_brightness_for_SPY555-DNA_comparison/e1575_p343_SPY-SNAP_zoom8_01-1_ch02.tif]

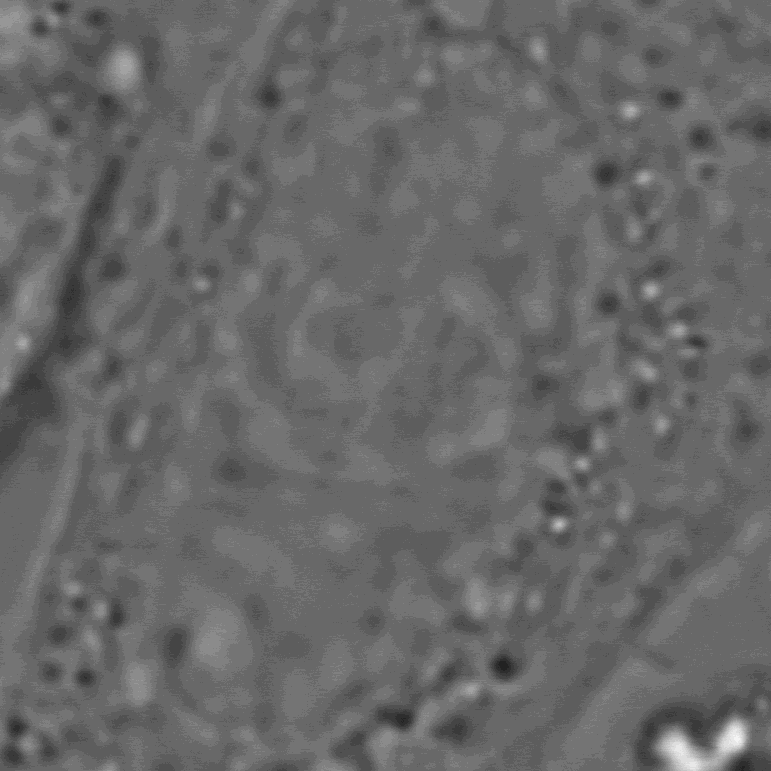

Supplement: Supplementary file 4 — Figure Source Data Appendix [file 44318_2026_747_MOESM4_ESM.zip › Appendix_Figure_S6/02_gray_scale_same_brightness_for_SPY555-DNA_comparison/e1575_p343_SPY-SNAP_zoom8_01-1_ch03.tif]

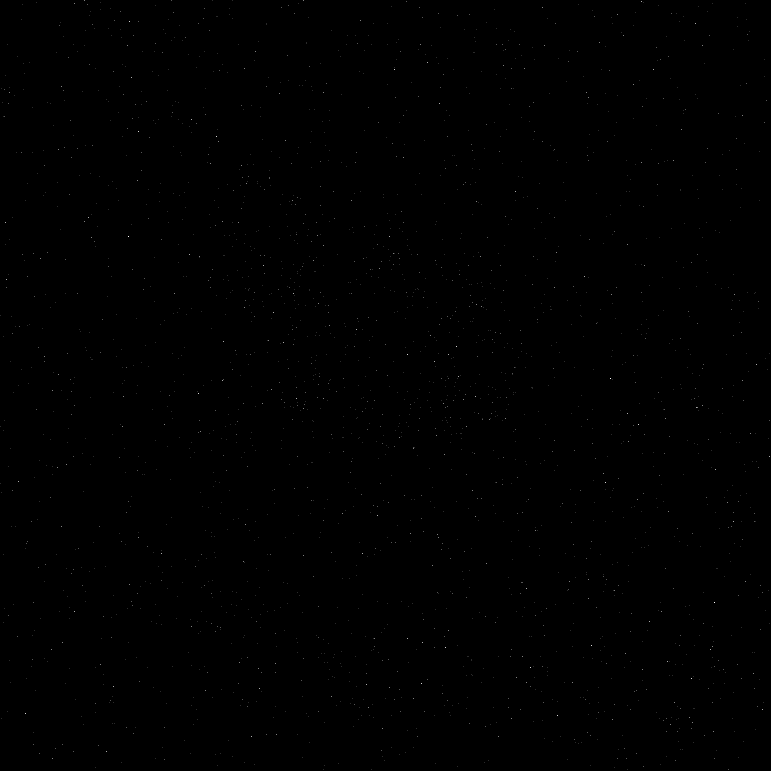

Supplement: Supplementary file 4 — Figure Source Data Appendix [file 44318_2026_747_MOESM4_ESM.zip › Appendix_Figure_S6/02_gray_scale_same_brightness_for_SPY555-DNA_comparison/e1575_p343_NoLabelling_zoom8_03-1_ch02_high_exposure.tif]

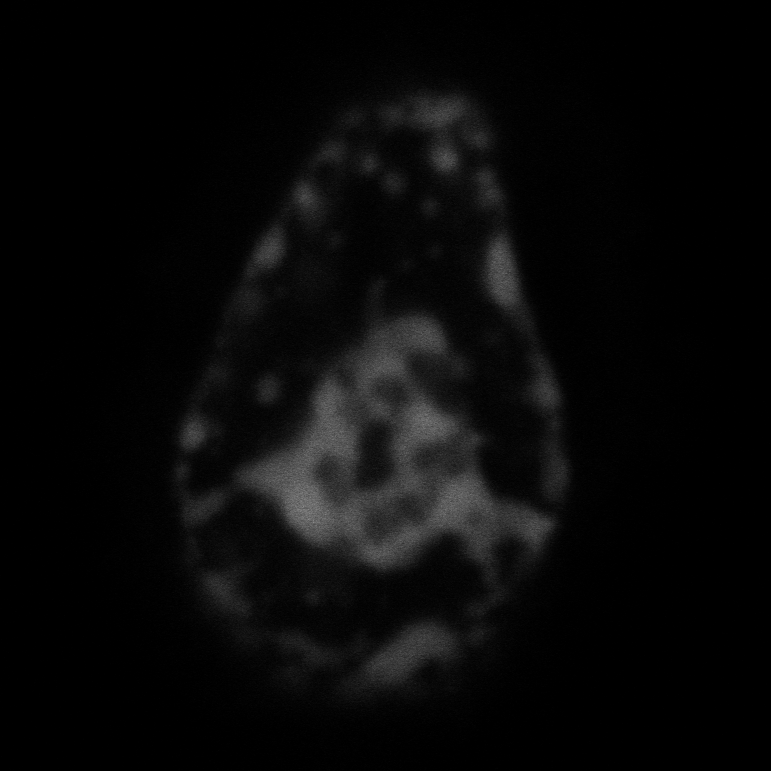

Supplement: Supplementary file 4 — Figure Source Data Appendix [file 44318_2026_747_MOESM4_ESM.zip › Appendix_Figure_S6/01_raw_data/e1575_p343_NoLabelling_zoom8_02-1.tif]

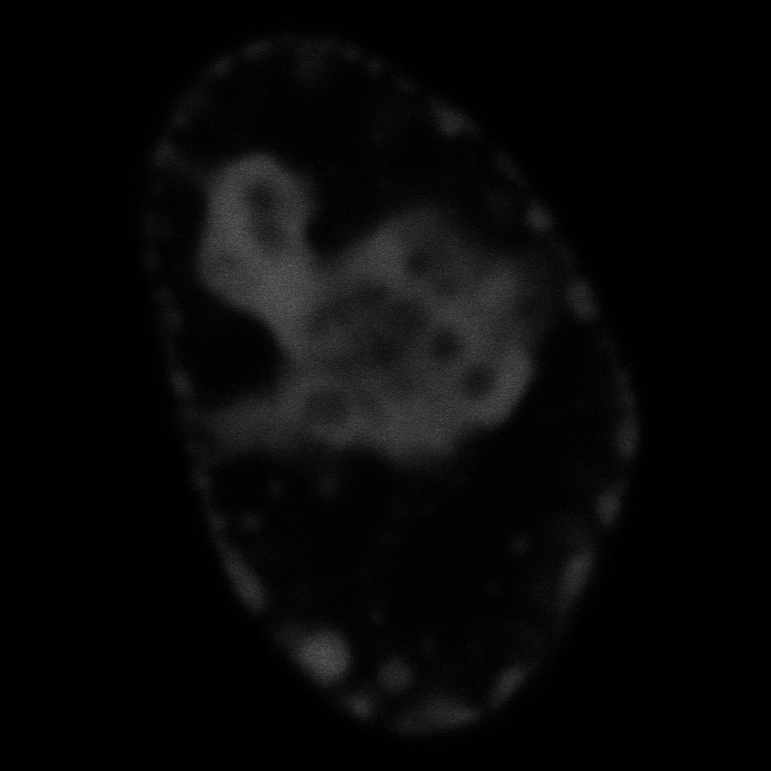

Supplement: Supplementary file 4 — Figure Source Data Appendix [file 44318_2026_747_MOESM4_ESM.zip › Appendix_Figure_S6/01_raw_data/e1575_p343_NoLabelling_zoom8_03-1.tif]

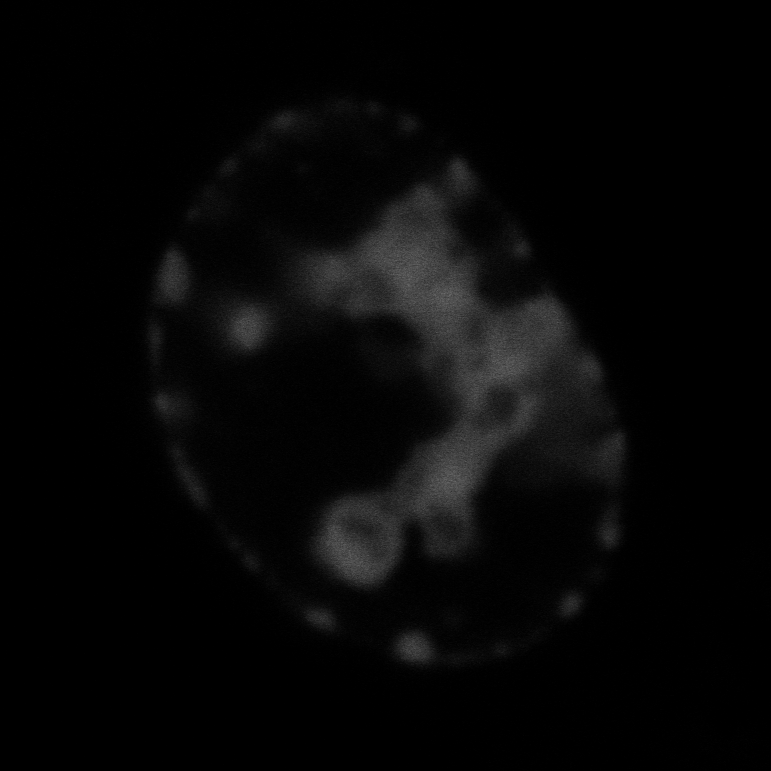

Supplement: Supplementary file 4 — Figure Source Data Appendix [file 44318_2026_747_MOESM4_ESM.zip › Appendix_Figure_S6/01_raw_data/e1575_p343_SiRDNA_zoom8_01-1.tif]

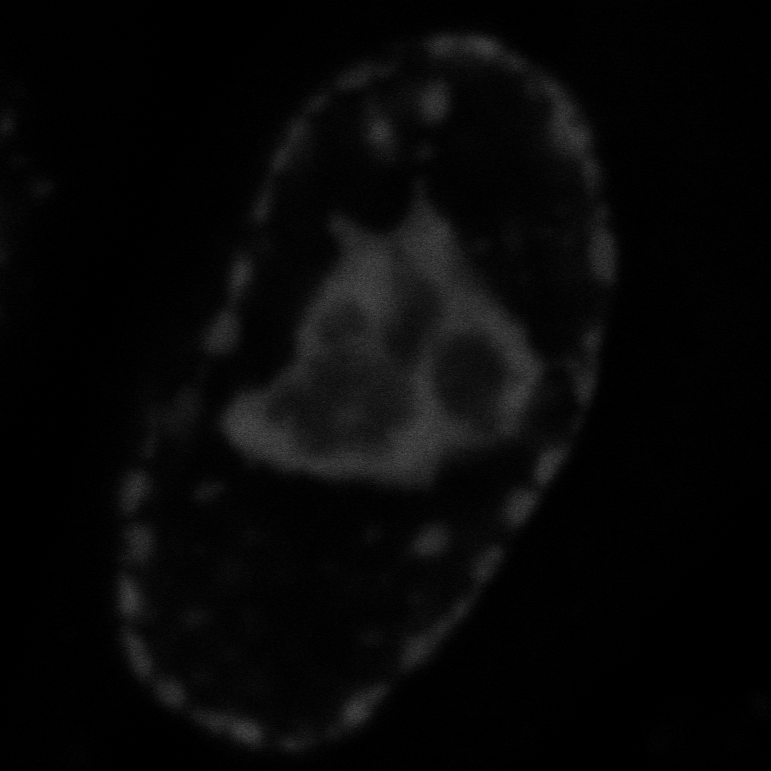

Supplement: Supplementary file 4 — Figure Source Data Appendix [file 44318_2026_747_MOESM4_ESM.zip › Appendix_Figure_S6/01_raw_data/e1575_p343_SPY-SNAP_zoom8_01-1.tif]

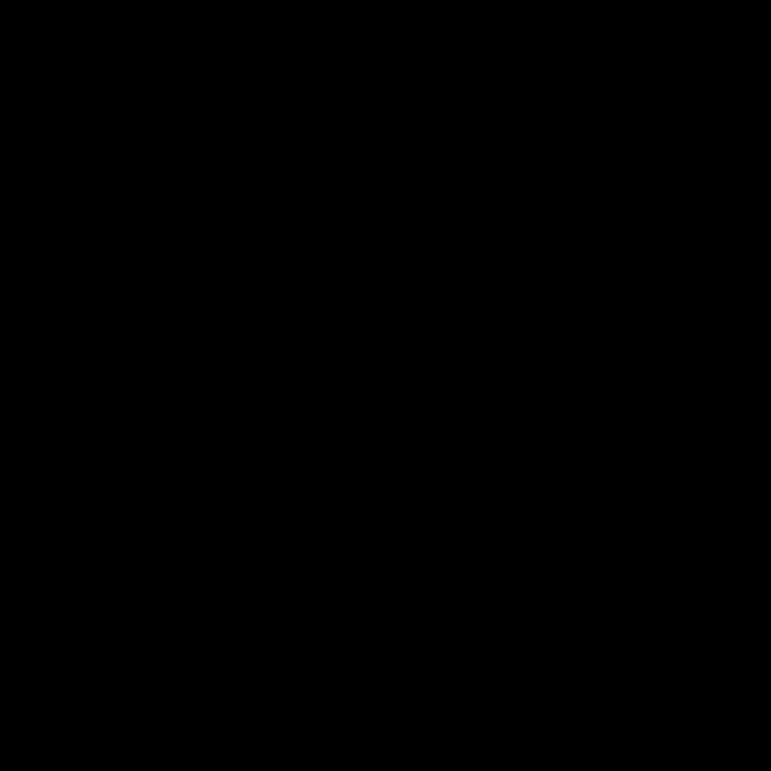

Supplement: Supplementary file 4 — Figure Source Data Appendix [file 44318_2026_747_MOESM4_ESM.zip › Appendix_Figure_S6/03_gray_scale_same_brightness_for_SiR-DNA_comparison/e1575_p343_SiRDNA_zoom8_01-1_ch04.tif]

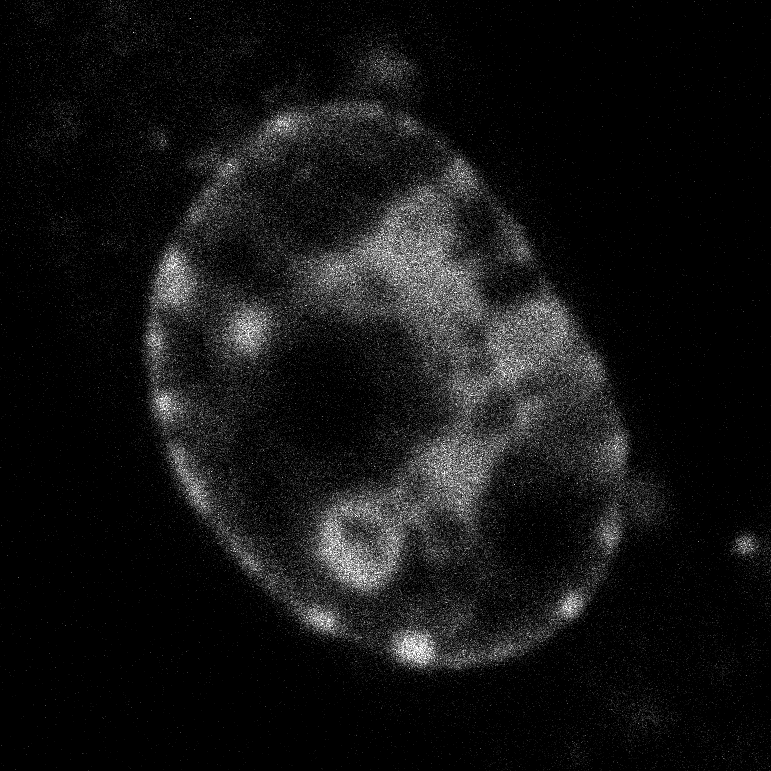

Supplement: Supplementary file 4 — Figure Source Data Appendix [file 44318_2026_747_MOESM4_ESM.zip › Appendix_Figure_S6/03_gray_scale_same_brightness_for_SiR-DNA_comparison/e1575_p343_SiRDNA_zoom8_01-1_ch02.tif]

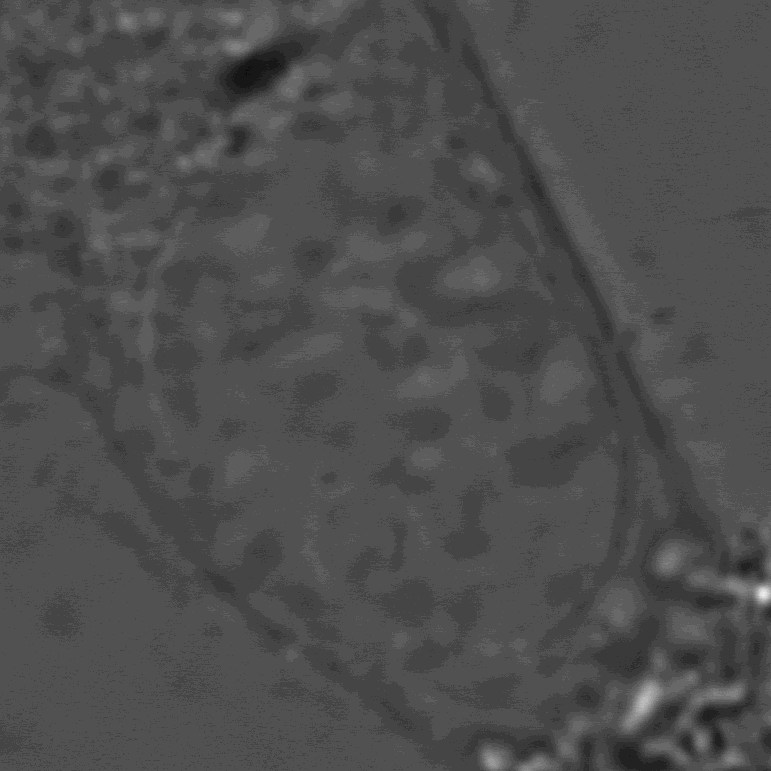

Supplement: Supplementary file 4 — Figure Source Data Appendix [file 44318_2026_747_MOESM4_ESM.zip › Appendix_Figure_S6/03_gray_scale_same_brightness_for_SiR-DNA_comparison/e1575_p343_SiRDNA_zoom8_01-1_ch03.tif]

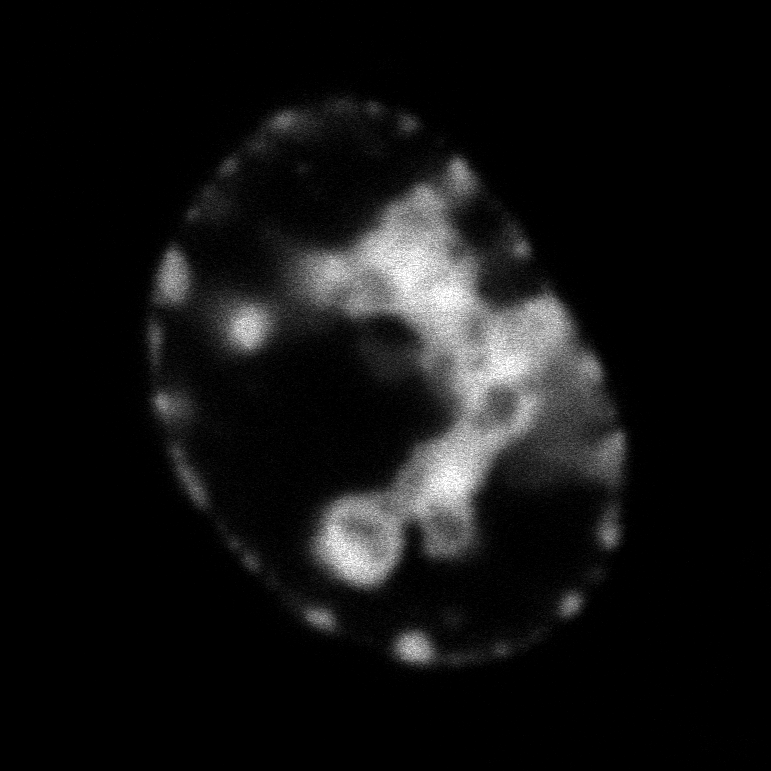

Supplement: Supplementary file 4 — Figure Source Data Appendix [file 44318_2026_747_MOESM4_ESM.zip › Appendix_Figure_S6/03_gray_scale_same_brightness_for_SiR-DNA_comparison/e1575_p343_SiRDNA_zoom8_01-1_ch01.tif]

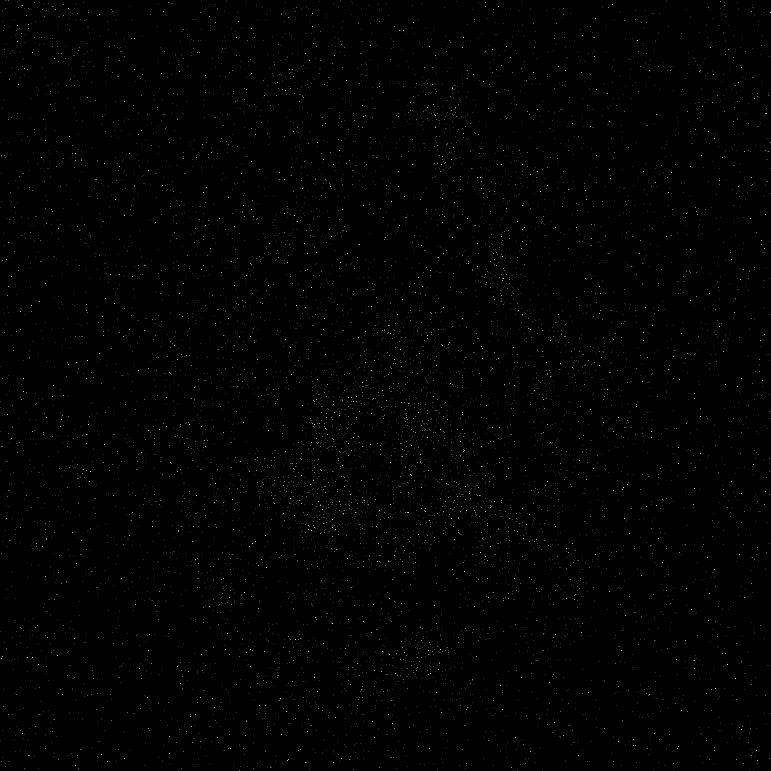

Supplement: Supplementary file 4 — Figure Source Data Appendix [file 44318_2026_747_MOESM4_ESM.zip › Appendix_Figure_S6/03_gray_scale_same_brightness_for_SiR-DNA_comparison/e1575_p343_NoLabelling_zoom8_02-1_ch02_high_exposure.tif]

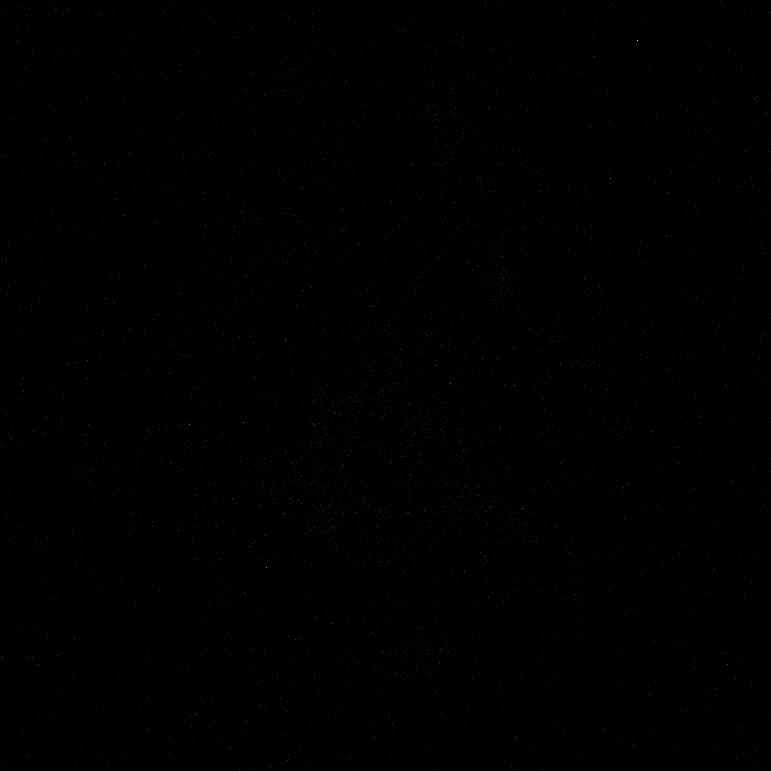

Supplement: Supplementary file 4 — Figure Source Data Appendix [file 44318_2026_747_MOESM4_ESM.zip › Appendix_Figure_S6/03_gray_scale_same_brightness_for_SiR-DNA_comparison/e1575_p343_NoLabelling_zoom8_02-1_ch02.tif]

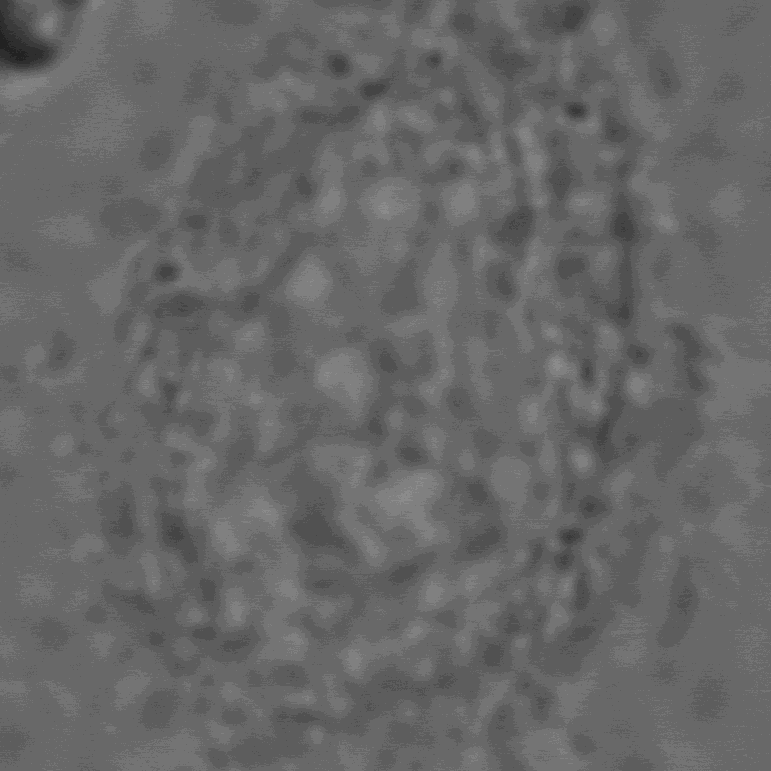

Supplement: Supplementary file 4 — Figure Source Data Appendix [file 44318_2026_747_MOESM4_ESM.zip › Appendix_Figure_S6/03_gray_scale_same_brightness_for_SiR-DNA_comparison/e1575_p343_NoLabelling_zoom8_02-1_ch03.tif]

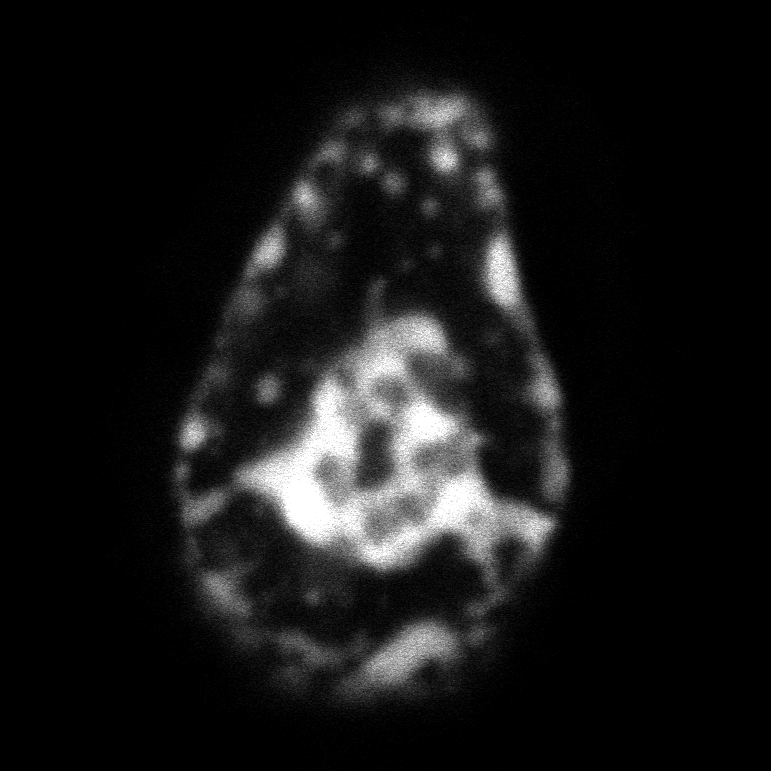

Supplement: Supplementary file 4 — Figure Source Data Appendix [file 44318_2026_747_MOESM4_ESM.zip › Appendix_Figure_S6/03_gray_scale_same_brightness_for_SiR-DNA_comparison/e1575_p343_NoLabelling_zoom8_02-1_ch01.tif]

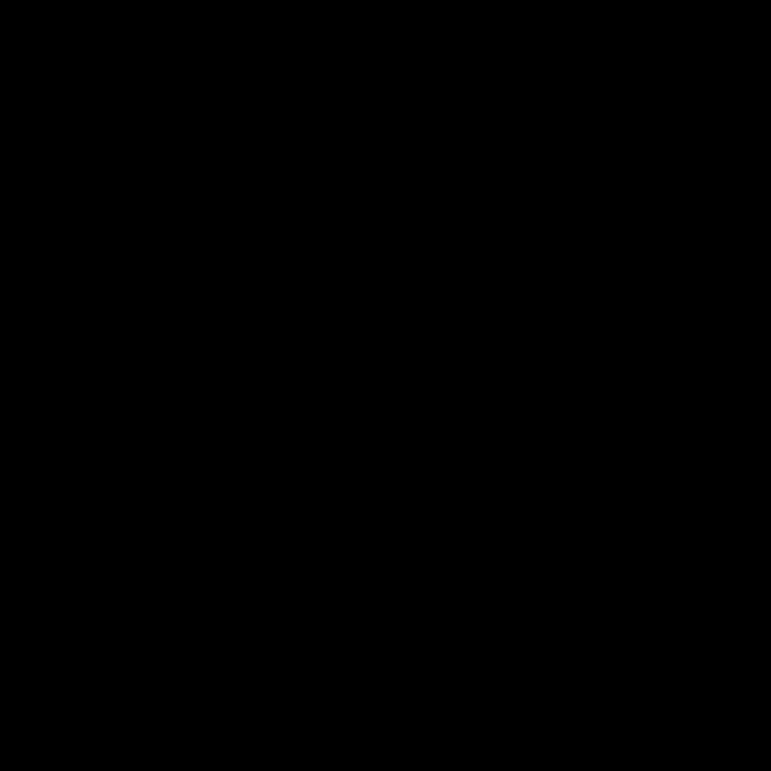

Supplement: Supplementary file 4 — Figure Source Data Appendix [file 44318_2026_747_MOESM4_ESM.zip › Appendix_Figure_S6/03_gray_scale_same_brightness_for_SiR-DNA_comparison/e1575_p343_NoLabelling_zoom8_02-1_ch04.tif]

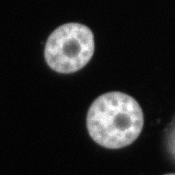

Supplement: Supplementary file 5 — Source data Fig. 1 [file 44318_2026_747_MOESM5_ESM.zip › Figure 1/C/raw_images/e-0337_W0197--s28830--BRWD1_P004--s28830--BRWD1_cropped.tif]

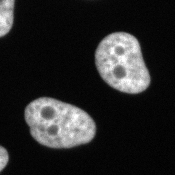

Supplement: Supplementary file 5 — Source data Fig. 1 [file 44318_2026_747_MOESM5_ESM.zip › Figure 1/C/raw_images/e-0470_W0001--s43866--WDR36_P004--s43866--WDR36_cropped.tif]

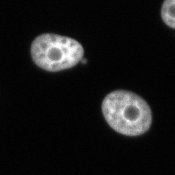

Supplement: Supplementary file 5 — Source data Fig. 1 [file 44318_2026_747_MOESM5_ESM.zip › Figure 1/C/raw_images/e-0470_W0332--s21380--WDR3_P002--s21380--WDR3_cropped.tif]

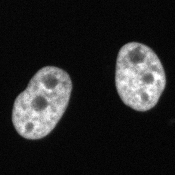

Supplement: Supplementary file 5 — Source data Fig. 1 [file 44318_2026_747_MOESM5_ESM.zip › Figure 1/C/raw_images/e-0470_W0004--s20641--NOP56_P002--s20641--NOP56_cropped.tif]

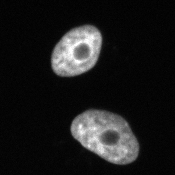

Supplement: Supplementary file 5 — Source data Fig. 1 [file 44318_2026_747_MOESM5_ESM.zip › Figure 1/C/raw_images/e-0470_W0201--s19196--RBM19_P004--s19196--RBM19_cropped.tif]

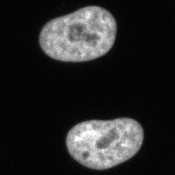

Supplement: Supplementary file 5 — Source data Fig. 1 [file 44318_2026_747_MOESM5_ESM.zip › Figure 1/C/raw_images/e-0470_W0019--s444246--XWNeg9_P002--s444246--XWNeg9_cropped.tif]

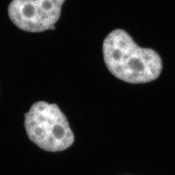

Supplement: Supplementary file 5 — Source data Fig. 1 [file 44318_2026_747_MOESM5_ESM.zip › Figure 1/C/raw_images/e-0470_W0329--s16357--NOP14_P001--s16357--NOP14_cropped.tif]

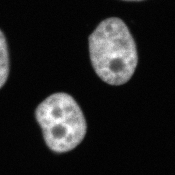

Supplement: Supplementary file 5 — Source data Fig. 1 [file 44318_2026_747_MOESM5_ESM.zip › Figure 1/C/raw_images/e-0335_W0240--s20823--TBL3_P003--s20823--TBL3_cropped.tif]

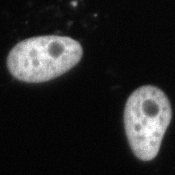

Supplement: Supplementary file 5 — Source data Fig. 1 [file 44318_2026_747_MOESM5_ESM.zip › Figure 1/C/raw_images/e-0337_W0014--s14952--XRCC5_P004--s14952--XRCC5_cropped.tif]

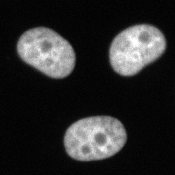

Supplement: Supplementary file 5 — Source data Fig. 1 [file 44318_2026_747_MOESM5_ESM.zip › Figure 1/C/raw_images/e-0470_W0216--s31575--UTP6_P003--s31575--UTP6_cropped.tif]

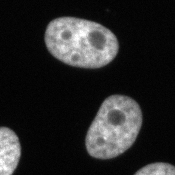

Supplement: Supplementary file 5 — Source data Fig. 1 [file 44318_2026_747_MOESM5_ESM.zip › Figure 1/C/raw_images/e-0274_W0353--s8796--MKI67_P004--s8796--MKI67_cropped.tif]

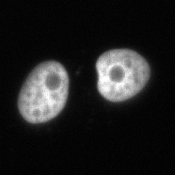

Supplement: Supplementary file 5 — Source data Fig. 1 [file 44318_2026_747_MOESM5_ESM.zip › Figure 1/C/raw_images/e-0337_W0101--s5456--XRCC6_P003--s5456--XRCC6_cropped.tif]

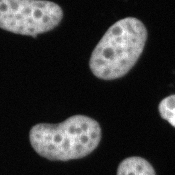

Supplement: Supplementary file 5 — Source data Fig. 1 [file 44318_2026_747_MOESM5_ESM.zip › Figure 1/C/raw_images/e-0482_W0159--s224296--POLR2E_P001--s224296--POLR2E_cropped.tif]

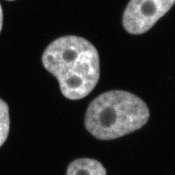

Supplement: Supplementary file 5 — Source data Fig. 1 [file 44318_2026_747_MOESM5_ESM.zip › Figure 1/C/raw_images/e-0470_W0159--s19879--MPHOSPH10_P002--s19879--MPHOSPH10_cropped.tif]

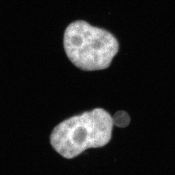

Supplement: Supplementary file 5 — Source data Fig. 1 [file 44318_2026_747_MOESM5_ESM.zip › Figure 1/C/raw_images/e-0470_W0203--s22772--PDCD11_P004--s22772--PDCD11_cropped.tif]

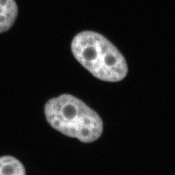

Supplement: Supplementary file 5 — Source data Fig. 1 [file 44318_2026_747_MOESM5_ESM.zip › Figure 1/C/raw_images/e-0470_W0168--s4821--FBL_P001--s4821--FBL_cropped.tif]

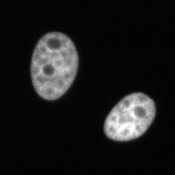

Supplement: Supplementary file 5 — Source data Fig. 1 [file 44318_2026_747_MOESM5_ESM.zip › Figure 1/C/raw_images/e-0470_W0041--s26948--DNTTIP2_P001--s26948--DNTTIP2_cropped.tif]

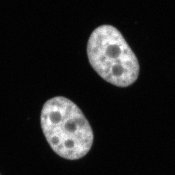

Supplement: Supplementary file 5 — Source data Fig. 1 [file 44318_2026_747_MOESM5_ESM.zip › Figure 1/C/raw_images/e-0470_W0204--s11610--PWP2_P004--s11610--PWP2_cropped.tif]

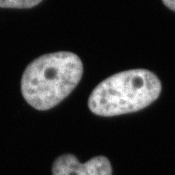

Supplement: Supplementary file 5 — Source data Fig. 1 [file 44318_2026_747_MOESM5_ESM.zip › Figure 1/C/raw_images/e-0274_W0062--s38530--WDR75_P004--s38530--WDR75_cropped.tif]

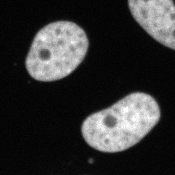

Supplement: Supplementary file 5 — Source data Fig. 1 [file 44318_2026_747_MOESM5_ESM.zip › Figure 1/C/raw_images/e-0470_W0370--s24764--NOL11_P002--s24764--NOL11_cropped.tif]

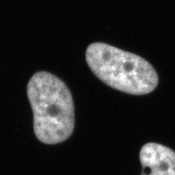

Supplement: Supplementary file 5 — Source data Fig. 1 [file 44318_2026_747_MOESM5_ESM.zip › Figure 1/C/raw_images/e-0470_W0356--s38549--UTP15_P002--s38549--UTP15_cropped.tif]

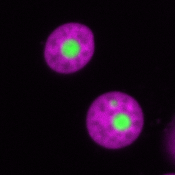

Supplement: Supplementary file 5 — Source data Fig. 1 [file 44318_2026_747_MOESM5_ESM.zip › Figure 1/C/RGB_images/e-0337_W0197--s28830--BRWD1_P004--s28830--BRWD1_cropped.tif]

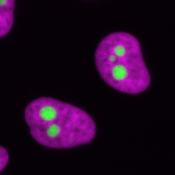

Supplement: Supplementary file 5 — Source data Fig. 1 [file 44318_2026_747_MOESM5_ESM.zip › Figure 1/C/RGB_images/e-0470_W0001--s43866--WDR36_P004--s43866--WDR36_cropped.tif]

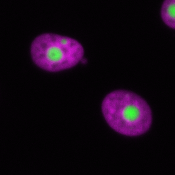

Supplement: Supplementary file 5 — Source data Fig. 1 [file 44318_2026_747_MOESM5_ESM.zip › Figure 1/C/RGB_images/e-0470_W0332--s21380--WDR3_P002--s21380--WDR3_cropped.tif]

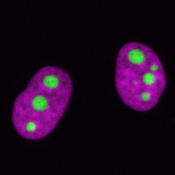

Supplement: Supplementary file 5 — Source data Fig. 1 [file 44318_2026_747_MOESM5_ESM.zip › Figure 1/C/RGB_images/e-0470_W0004--s20641--NOP56_P002--s20641--NOP56_cropped.tif]

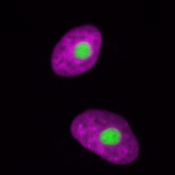

Supplement: Supplementary file 5 — Source data Fig. 1 [file 44318_2026_747_MOESM5_ESM.zip › Figure 1/C/RGB_images/e-0470_W0201--s19196--RBM19_P004--s19196--RBM19_cropped.tif]

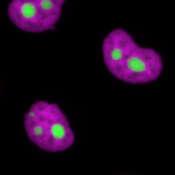

Supplement: Supplementary file 5 — Source data Fig. 1 [file 44318_2026_747_MOESM5_ESM.zip › Figure 1/C/RGB_images/e-0470_W0329--s16357--NOP14_P001--s16357--NOP14_cropped.tif]

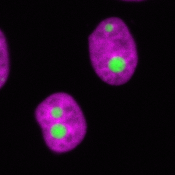

Supplement: Supplementary file 5 — Source data Fig. 1 [file 44318_2026_747_MOESM5_ESM.zip › Figure 1/C/RGB_images/e-0335_W0240--s20823--TBL3_P003--s20823--TBL3_cropped.tif]

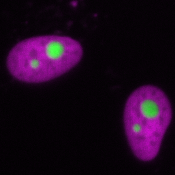

Supplement: Supplementary file 5 — Source data Fig. 1 [file 44318_2026_747_MOESM5_ESM.zip › Figure 1/C/RGB_images/e-0337_W0014--s14952--XRCC5_P004--s14952--XRCC5_cropped.tif]

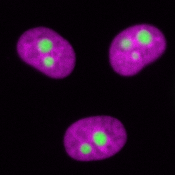

Supplement: Supplementary file 5 — Source data Fig. 1 [file 44318_2026_747_MOESM5_ESM.zip › Figure 1/C/RGB_images/e-0470_W0216--s31575--UTP6_P003--s31575--UTP6_cropped.tif]

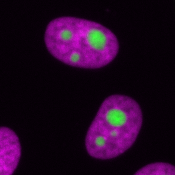

Supplement: Supplementary file 5 — Source data Fig. 1 [file 44318_2026_747_MOESM5_ESM.zip › Figure 1/C/RGB_images/e-0274_W0353--s8796--MKI67_P004--s8796--MKI67_cropped.tif]

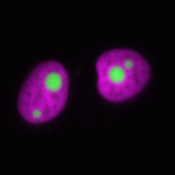

Supplement: Supplementary file 5 — Source data Fig. 1 [file 44318_2026_747_MOESM5_ESM.zip › Figure 1/C/RGB_images/e-0337_W0101--s5456--XRCC6_P003--s5456--XRCC6_cropped.tif]

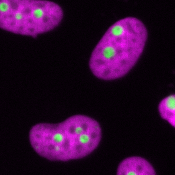

Supplement: Supplementary file 5 — Source data Fig. 1 [file 44318_2026_747_MOESM5_ESM.zip › Figure 1/C/RGB_images/e-0482_W0159--s224296--POLR2E_P001--s224296--POLR2E_cropped.tif]
