# Supplementary figures and images for: Ki-67 shapes the nucleolus by anchoring chromatin via its amphiphilic properties (part 1 of 5)
Source: EMBO J. 2026 Mar 24;45(9):3156–91. doi: 10.1038/s44318-026-00747-7 (PMC13144362; doi:10.1038/s44318-026-00747-7)

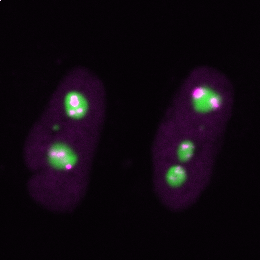

Supplement: Supplementary file 4 — Figure Source Data Appendix [file 44318_2026_747_MOESM4_ESM.zip › Appendix_Figure_S1/RGB/e-0829_W0019--s22772--PDCD11_P00004--s38965--NIFK_cropped.tif]

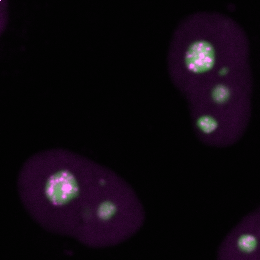

Supplement: Supplementary file 4 — Figure Source Data Appendix [file 44318_2026_747_MOESM4_ESM.zip › Appendix_Figure_S1/RGB/e-0829_W0061--s21380--WDR3_P00001--s13210--SNRNP70_cropped.tif]

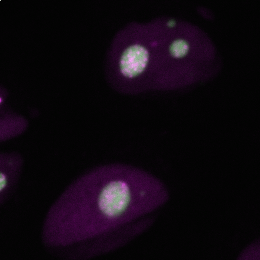

Supplement: Supplementary file 4 — Figure Source Data Appendix [file 44318_2026_747_MOESM4_ESM.zip › Appendix_Figure_S1/RGB/e-0829_W0094--s20823--TBL3_P00004--empty--empty_cropped.tif]

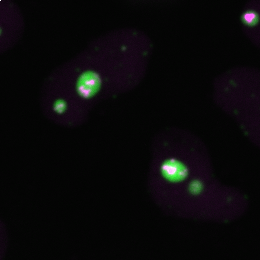

Supplement: Supplementary file 4 — Figure Source Data Appendix [file 44318_2026_747_MOESM4_ESM.zip › Appendix_Figure_S1/RGB/e-0829_W0059--s26948--DNTTIP2_P00002--s14615--UBTF_cropped.tif]

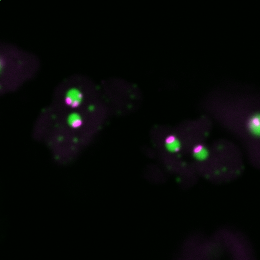

Supplement: Supplementary file 4 — Figure Source Data Appendix [file 44318_2026_747_MOESM4_ESM.zip › Appendix_Figure_S1/RGB/e-0829_W0066--s24764--NOL11_P00004--s18923--BMS1_cropped.tif]

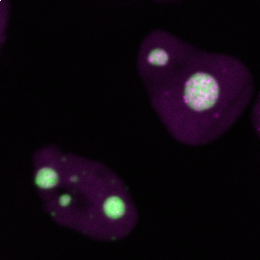

Supplement: Supplementary file 4 — Figure Source Data Appendix [file 44318_2026_747_MOESM4_ESM.zip › Appendix_Figure_S1/RGB/e-0829_W0069--s43866--WDR36_P00004--s14614--UBTF_cropped.tif]

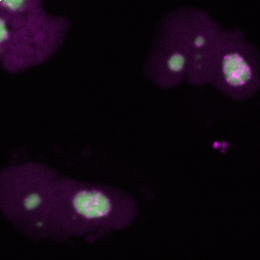

Supplement: Supplementary file 4 — Figure Source Data Appendix [file 44318_2026_747_MOESM4_ESM.zip › Appendix_Figure_S1/RGB/e-0829_W0022--s20641--NOP56_P00002--s444246--XWNeg9_cropped.tif]

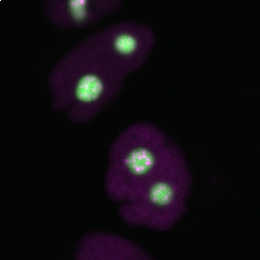

Supplement: Supplementary file 4 — Figure Source Data Appendix [file 44318_2026_747_MOESM4_ESM.zip › Appendix_Figure_S1/RGB/e-0829_W0036--s16357--NOP14_P00003--s7424--INCENP_cropped.tif]

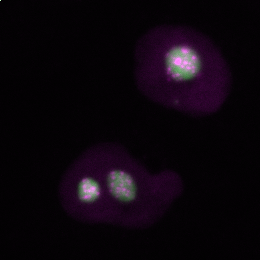

Supplement: Supplementary file 4 — Figure Source Data Appendix [file 44318_2026_747_MOESM4_ESM.zip › Appendix_Figure_S1/RGB/e-0829_W0009--s31575--UTP6_P00001--s28120--DDX41_cropped.tif]

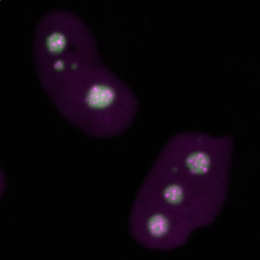

Supplement: Supplementary file 4 — Figure Source Data Appendix [file 44318_2026_747_MOESM4_ESM.zip › Appendix_Figure_S1/RGB/e-0829_W0081--s8796--MKI67_P00004--s15706--CHAF1B_cropped.tif]

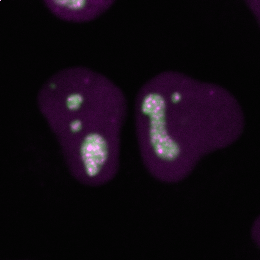

Supplement: Supplementary file 4 — Figure Source Data Appendix [file 44318_2026_747_MOESM4_ESM.zip › Appendix_Figure_S1/RGB/e-0829_W0086--s444246--XWNeg9_P00004--s223399--DDX10_cropped.tif]

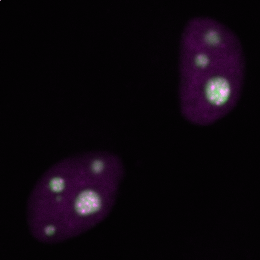

Supplement: Supplementary file 4 — Figure Source Data Appendix [file 44318_2026_747_MOESM4_ESM.zip › Appendix_Figure_S1/RGB/e-0829_W0075--s14952--XRCC5_P00003--s226817--PAK1IP1_cropped.tif]

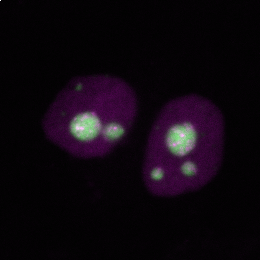

Supplement: Supplementary file 4 — Figure Source Data Appendix [file 44318_2026_747_MOESM4_ESM.zip › Appendix_Figure_S1/RGB/e-0829_W0070--s11610--PWP2_P00001--s25541--AATF_cropped.tif]

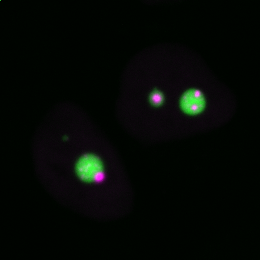

Supplement: Supplementary file 4 — Figure Source Data Appendix [file 44318_2026_747_MOESM4_ESM.zip › Appendix_Figure_S1/RGB/e-0829_W0047--s224296--POLR2E_P00003--s18922--BMS1_cropped.tif]

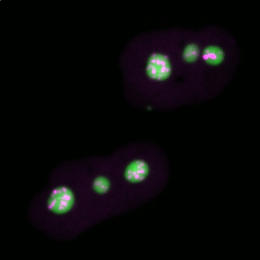

Supplement: Supplementary file 4 — Figure Source Data Appendix [file 44318_2026_747_MOESM4_ESM.zip › Appendix_Figure_S1/RGB/e-0829_W0068--s19879--MPHOSPH10_P00003--s28389--NOP58_cropped.tif]

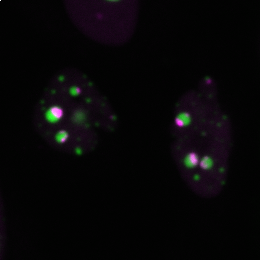

Supplement: Supplementary file 4 — Figure Source Data Appendix [file 44318_2026_747_MOESM4_ESM.zip › Appendix_Figure_S1/RGB/e-0829_W0016--s38549--UTP15_P00001--s14306--TOP1_cropped.tif]

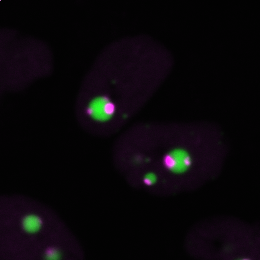

Supplement: Supplementary file 4 — Figure Source Data Appendix [file 44318_2026_747_MOESM4_ESM.zip › Appendix_Figure_S1/RGB/e-0829_W0063--s38530--WDR75_P00004--s4022--DDX10_cropped.tif]

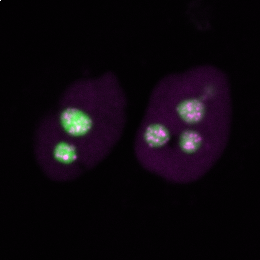

Supplement: Supplementary file 4 — Figure Source Data Appendix [file 44318_2026_747_MOESM4_ESM.zip › Appendix_Figure_S1/RGB/e-0829_W0043--s19196--RBM19_P00001--s23915--PES1_cropped.tif]

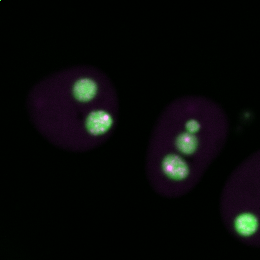

Supplement: Supplementary file 4 — Figure Source Data Appendix [file 44318_2026_747_MOESM4_ESM.zip › Appendix_Figure_S1/RGB/e-0829_W0045--s28830--BRWD1_P00003--s4023--DDX10_cropped.tif]

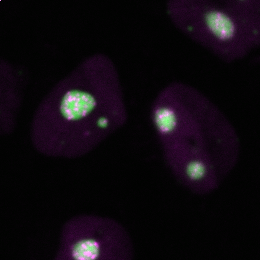

Supplement: Supplementary file 4 — Figure Source Data Appendix [file 44318_2026_747_MOESM4_ESM.zip › Appendix_Figure_S1/RGB/e-0829_W0023--s4821--FBL_P00001--s24668--DCAF13_cropped.tif]

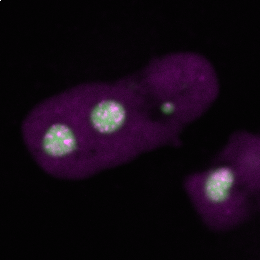

Supplement: Supplementary file 4 — Figure Source Data Appendix [file 44318_2026_747_MOESM4_ESM.zip › Appendix_Figure_S1/RGB/e-0829_W0027--s5456--XRCC6_P00003--s17019--PRPF4B_cropped.tif]

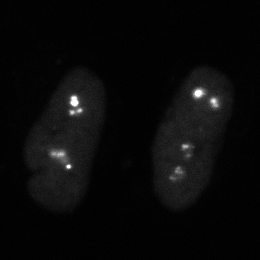

Supplement: Supplementary file 4 — Figure Source Data Appendix [file 44318_2026_747_MOESM4_ESM.zip › Appendix_Figure_S1/raw/e-0829_W0019--s22772--PDCD11_P00004--s38965--NIFK_cropped.tif]

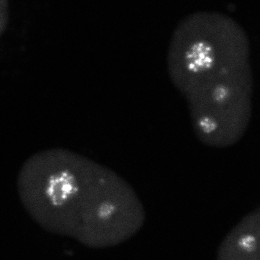

Supplement: Supplementary file 4 — Figure Source Data Appendix [file 44318_2026_747_MOESM4_ESM.zip › Appendix_Figure_S1/raw/e-0829_W0061--s21380--WDR3_P00001--s13210--SNRNP70_cropped.tif]

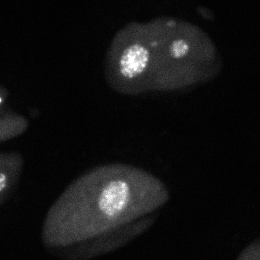

Supplement: Supplementary file 4 — Figure Source Data Appendix [file 44318_2026_747_MOESM4_ESM.zip › Appendix_Figure_S1/raw/e-0829_W0094--s20823--TBL3_P00004--empty--empty_cropped.tif]

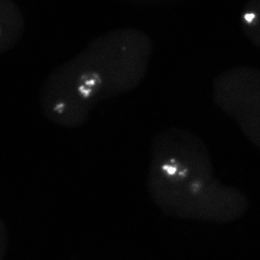

Supplement: Supplementary file 4 — Figure Source Data Appendix [file 44318_2026_747_MOESM4_ESM.zip › Appendix_Figure_S1/raw/e-0829_W0059--s26948--DNTTIP2_P00002--s14615--UBTF_cropped.tif]

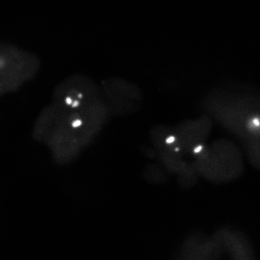

Supplement: Supplementary file 4 — Figure Source Data Appendix [file 44318_2026_747_MOESM4_ESM.zip › Appendix_Figure_S1/raw/e-0829_W0066--s24764--NOL11_P00004--s18923--BMS1_cropped.tif]

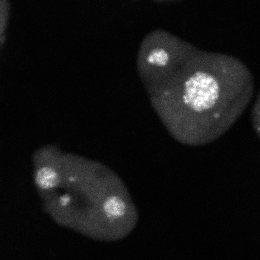

Supplement: Supplementary file 4 — Figure Source Data Appendix [file 44318_2026_747_MOESM4_ESM.zip › Appendix_Figure_S1/raw/e-0829_W0069--s43866--WDR36_P00004--s14614--UBTF_cropped.tif]

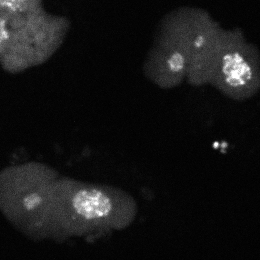

Supplement: Supplementary file 4 — Figure Source Data Appendix [file 44318_2026_747_MOESM4_ESM.zip › Appendix_Figure_S1/raw/e-0829_W0022--s20641--NOP56_P00002--s444246--XWNeg9_cropped.tif]

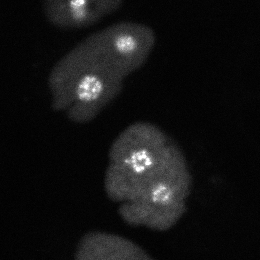

Supplement: Supplementary file 4 — Figure Source Data Appendix [file 44318_2026_747_MOESM4_ESM.zip › Appendix_Figure_S1/raw/e-0829_W0036--s16357--NOP14_P00003--s7424--INCENP_cropped.tif]

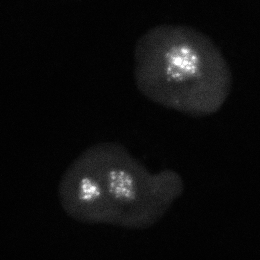

Supplement: Supplementary file 4 — Figure Source Data Appendix [file 44318_2026_747_MOESM4_ESM.zip › Appendix_Figure_S1/raw/e-0829_W0009--s31575--UTP6_P00001--s28120--DDX41_cropped.tif]

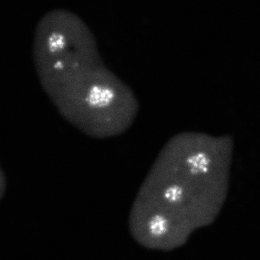

Supplement: Supplementary file 4 — Figure Source Data Appendix [file 44318_2026_747_MOESM4_ESM.zip › Appendix_Figure_S1/raw/e-0829_W0081--s8796--MKI67_P00004--s15706--CHAF1B_cropped.tif]

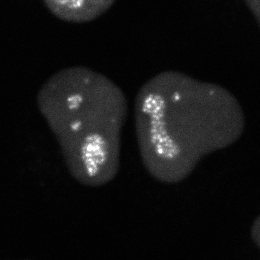

Supplement: Supplementary file 4 — Figure Source Data Appendix [file 44318_2026_747_MOESM4_ESM.zip › Appendix_Figure_S1/raw/e-0829_W0086--s444246--XWNeg9_P00004--s223399--DDX10_cropped.tif]

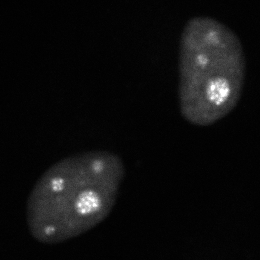

Supplement: Supplementary file 4 — Figure Source Data Appendix [file 44318_2026_747_MOESM4_ESM.zip › Appendix_Figure_S1/raw/e-0829_W0075--s14952--XRCC5_P00003--s226817--PAK1IP1_cropped.tif]

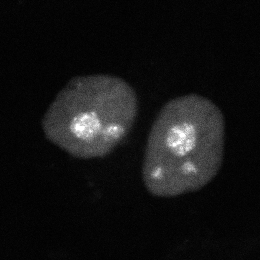

Supplement: Supplementary file 4 — Figure Source Data Appendix [file 44318_2026_747_MOESM4_ESM.zip › Appendix_Figure_S1/raw/e-0829_W0070--s11610--PWP2_P00001--s25541--AATF_cropped.tif]

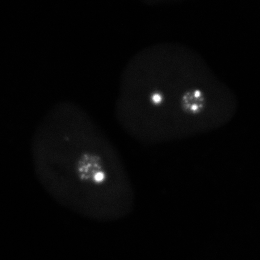

Supplement: Supplementary file 4 — Figure Source Data Appendix [file 44318_2026_747_MOESM4_ESM.zip › Appendix_Figure_S1/raw/e-0829_W0047--s224296--POLR2E_P00003--s18922--BMS1_cropped.tif]

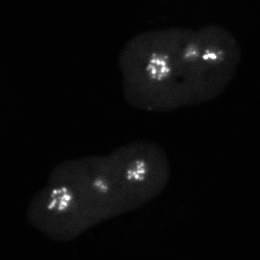

Supplement: Supplementary file 4 — Figure Source Data Appendix [file 44318_2026_747_MOESM4_ESM.zip › Appendix_Figure_S1/raw/e-0829_W0068--s19879--MPHOSPH10_P00003--s28389--NOP58_cropped.tif]

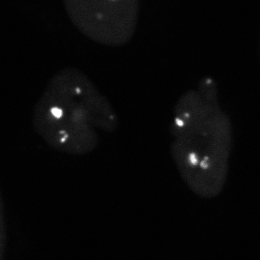

Supplement: Supplementary file 4 — Figure Source Data Appendix [file 44318_2026_747_MOESM4_ESM.zip › Appendix_Figure_S1/raw/e-0829_W0016--s38549--UTP15_P00001--s14306--TOP1_cropped.tif]

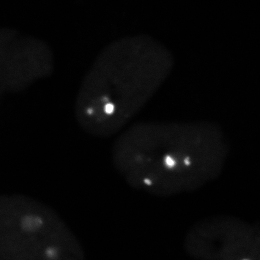

Supplement: Supplementary file 4 — Figure Source Data Appendix [file 44318_2026_747_MOESM4_ESM.zip › Appendix_Figure_S1/raw/e-0829_W0063--s38530--WDR75_P00004--s4022--DDX10_cropped.tif]

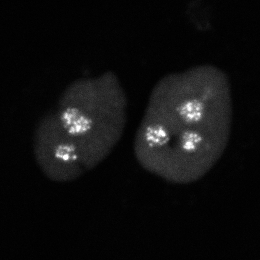

Supplement: Supplementary file 4 — Figure Source Data Appendix [file 44318_2026_747_MOESM4_ESM.zip › Appendix_Figure_S1/raw/e-0829_W0043--s19196--RBM19_P00001--s23915--PES1_cropped.tif]

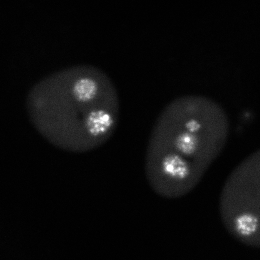

Supplement: Supplementary file 4 — Figure Source Data Appendix [file 44318_2026_747_MOESM4_ESM.zip › Appendix_Figure_S1/raw/e-0829_W0045--s28830--BRWD1_P00003--s4023--DDX10_cropped.tif]

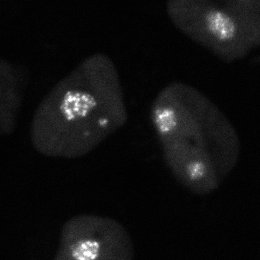

Supplement: Supplementary file 4 — Figure Source Data Appendix [file 44318_2026_747_MOESM4_ESM.zip › Appendix_Figure_S1/raw/e-0829_W0023--s4821--FBL_P00001--s24668--DCAF13_cropped.tif]

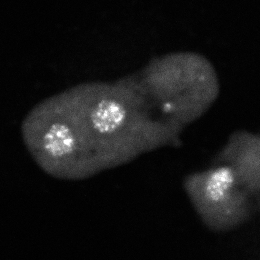

Supplement: Supplementary file 4 — Figure Source Data Appendix [file 44318_2026_747_MOESM4_ESM.zip › Appendix_Figure_S1/raw/e-0829_W0027--s5456--XRCC6_P00003--s17019--PRPF4B_cropped.tif]

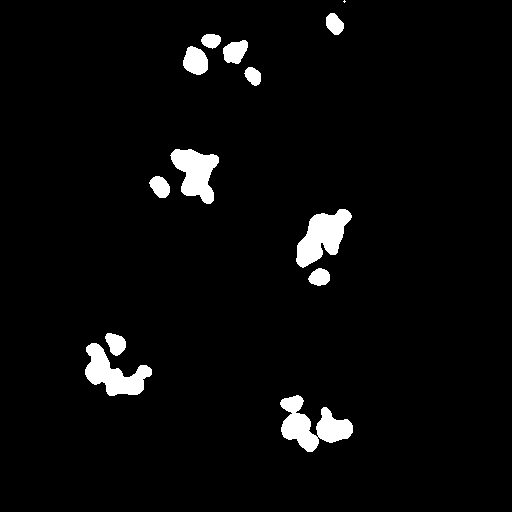

Supplement: Supplementary file 4 — Figure Source Data Appendix [file 44318_2026_747_MOESM4_ESM.zip › Appendix_Figure_S2/A/e-0727_28-05-2021_c-1_pos-26_RI_SEGMENTATION.png]

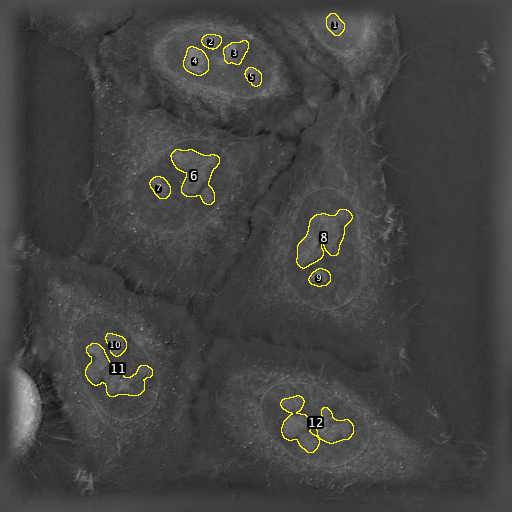

Supplement: Supplementary file 4 — Figure Source Data Appendix [file 44318_2026_747_MOESM4_ESM.zip › Appendix_Figure_S2/A/e-0727_28-05-2021_c-1_pos-26_RI_Z43_ROIs_OVERLAY.tif]

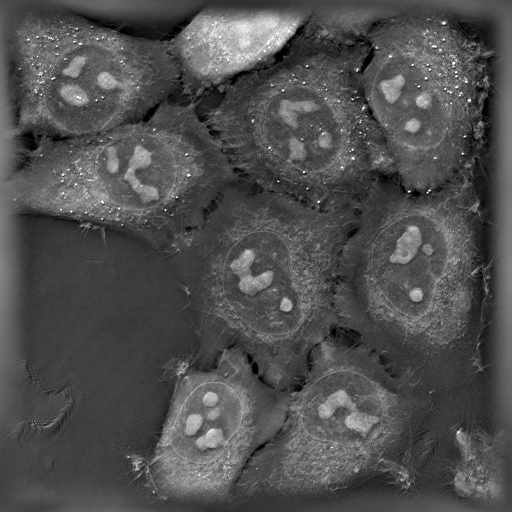

Supplement: Supplementary file 4 — Figure Source Data Appendix [file 44318_2026_747_MOESM4_ESM.zip › Appendix_Figure_S2/D/RGB/BC-adjusted-not-applied_e-0724_27-05-2021_c-1_pos-32_RI_Z44_RGB.tif]

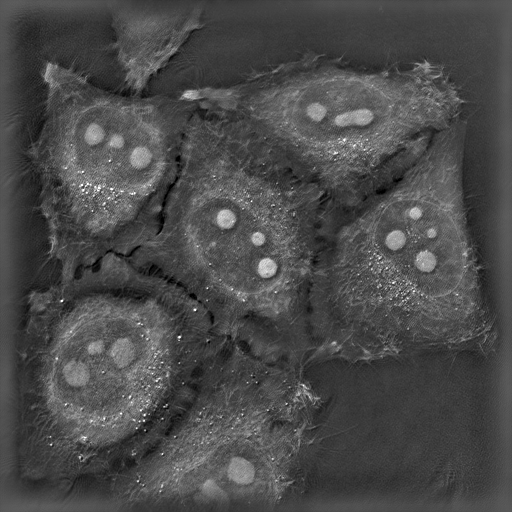

Supplement: Supplementary file 4 — Figure Source Data Appendix [file 44318_2026_747_MOESM4_ESM.zip › Appendix_Figure_S2/D/RGB/BC-adjusted-not-applied_e-0724_27-05-2021_c-63_pos-6_RI_Z38_RGB.tif]

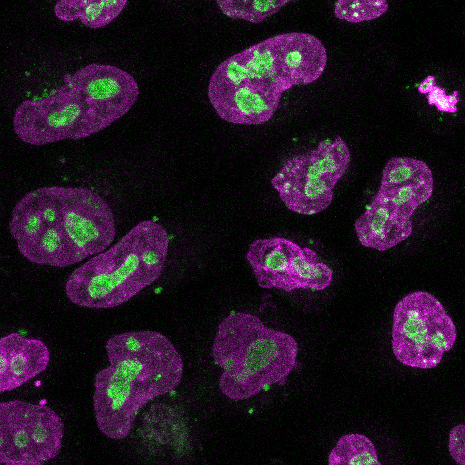

Supplement: Supplementary file 4 — Figure Source Data Appendix [file 44318_2026_747_MOESM4_ESM.zip › Appendix_Figure_S2/B/RGB/e1214_exp04_c77_siControl_01_maxProjection-1.tif (RGB).tif]

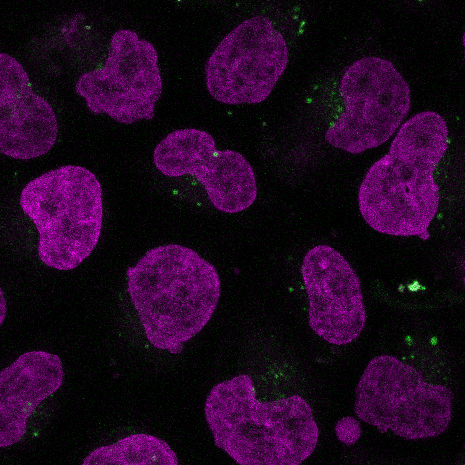

Supplement: Supplementary file 4 — Figure Source Data Appendix [file 44318_2026_747_MOESM4_ESM.zip › Appendix_Figure_S2/B/RGB/e1214_exp04_c77_siKI67_1_02_maxProjection-1.tif (RGB).tif]

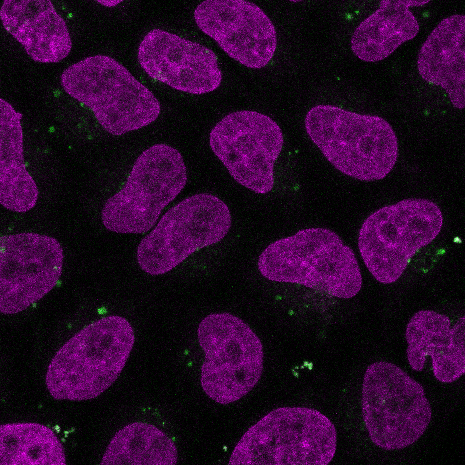

Supplement: Supplementary file 4 — Figure Source Data Appendix [file 44318_2026_747_MOESM4_ESM.zip › Appendix_Figure_S2/B/RGB/e1214_exp04_c77_siKI67_2_04_maxProjection-1.tif (RGB).tif]

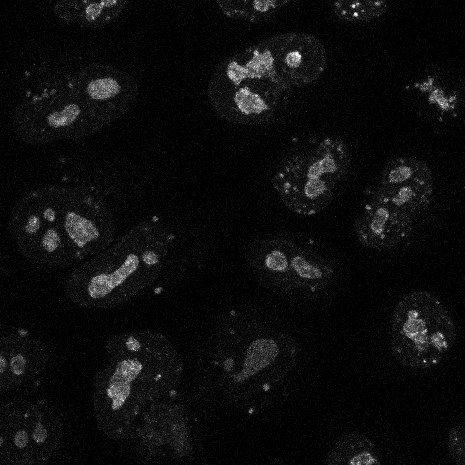

Supplement: Supplementary file 4 — Figure Source Data Appendix [file 44318_2026_747_MOESM4_ESM.zip › Appendix_Figure_S2/B/raw/e1214_exp04_c77_siControl_01_maxProjection-1.tif]

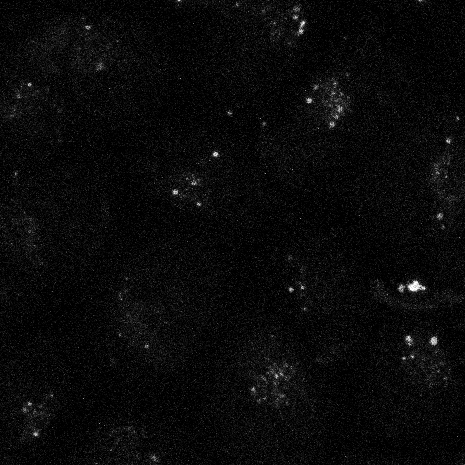

Supplement: Supplementary file 4 — Figure Source Data Appendix [file 44318_2026_747_MOESM4_ESM.zip › Appendix_Figure_S2/B/raw/e1214_exp04_c77_siKI67_1_02_maxProjection-1.tif]

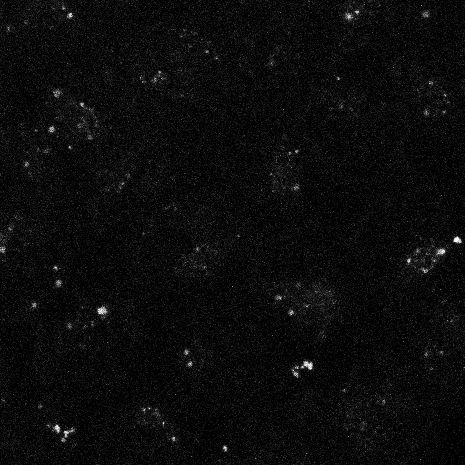

Supplement: Supplementary file 4 — Figure Source Data Appendix [file 44318_2026_747_MOESM4_ESM.zip › Appendix_Figure_S2/B/raw/e1214_exp04_c77_siKI67_2_04_maxProjection-1.tif]

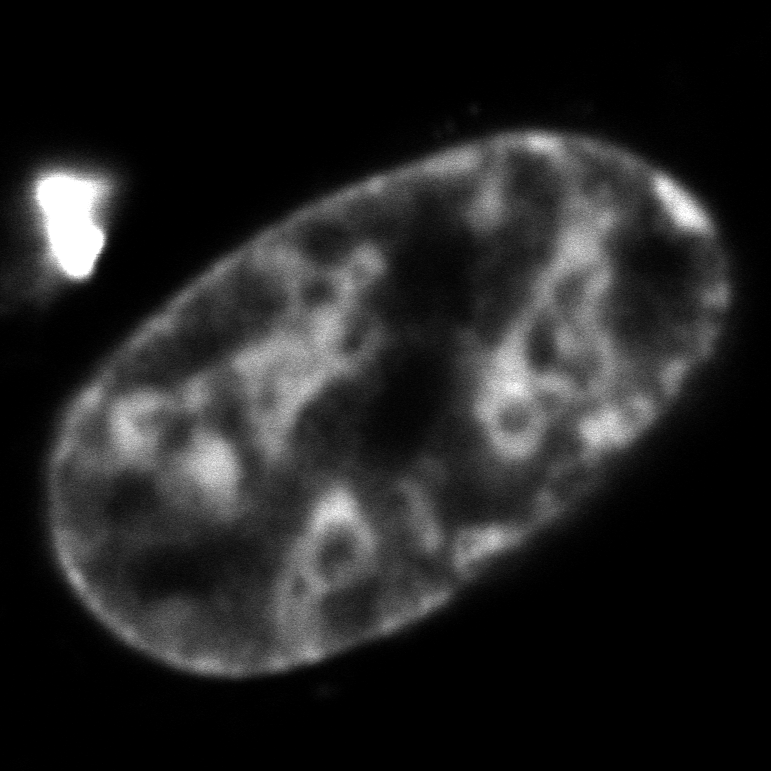

Supplement: Supplementary file 4 — Figure Source Data Appendix [file 44318_2026_747_MOESM4_ESM.zip › Appendix_Figure_S4/D/02_gray_scale_same_brightness/e1575_p1276_SPY-SNAP_zoom8_GFPplus_03-1_ch04.tif]

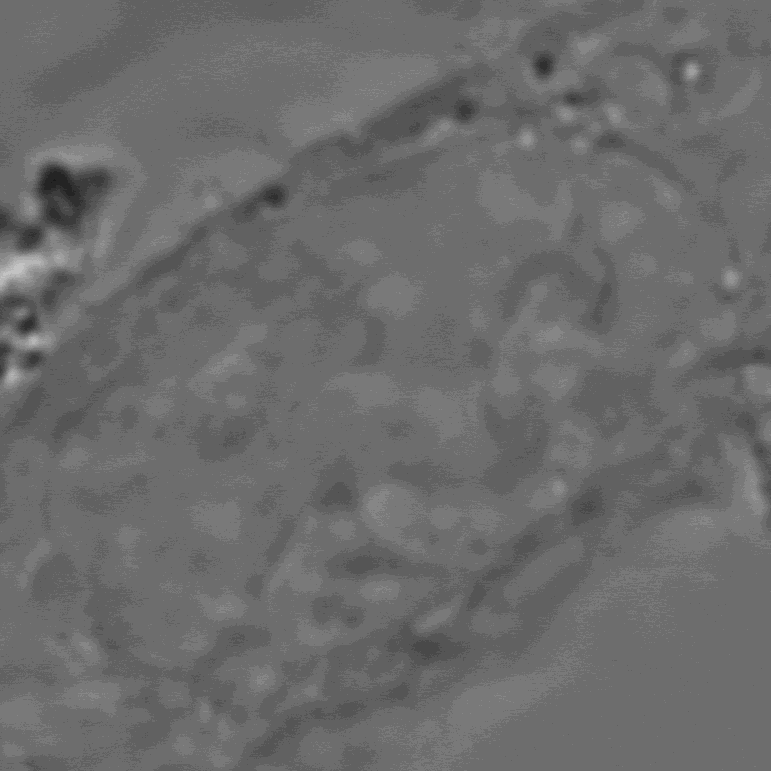

Supplement: Supplementary file 4 — Figure Source Data Appendix [file 44318_2026_747_MOESM4_ESM.zip › Appendix_Figure_S4/D/02_gray_scale_same_brightness/e1575_p1276_SPY-SNAP_zoom8_GFPplus_03-1_ch03.tif]

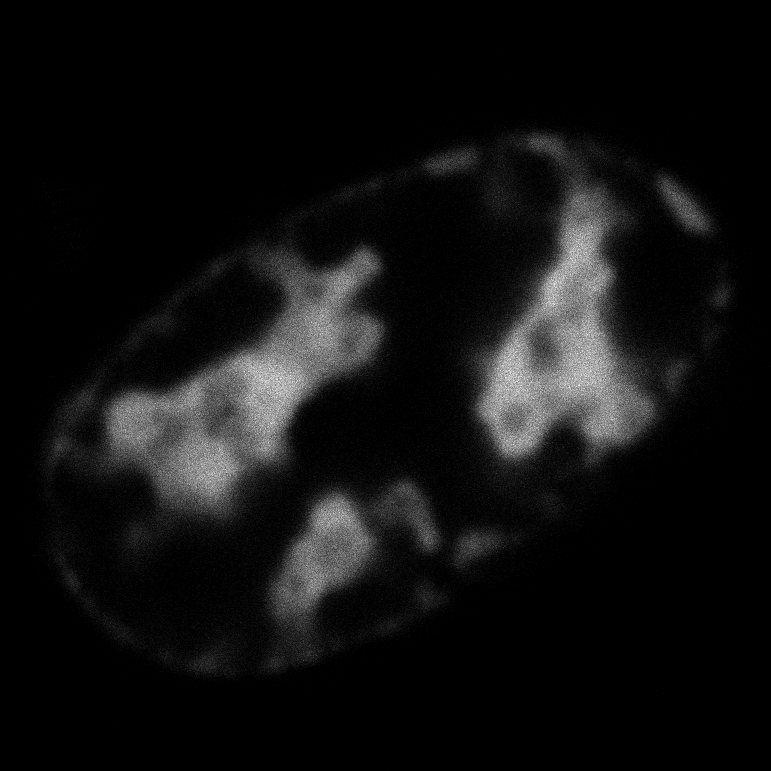

Supplement: Supplementary file 4 — Figure Source Data Appendix [file 44318_2026_747_MOESM4_ESM.zip › Appendix_Figure_S4/D/02_gray_scale_same_brightness/e1575_p1276_SPY-SNAP_zoom8_GFPplus_03-1_ch02.tif]

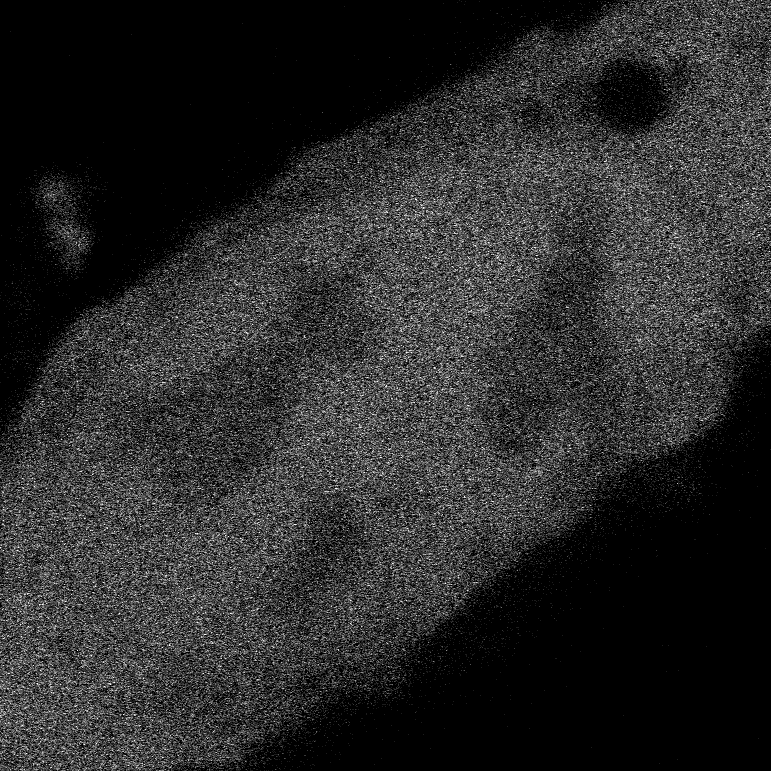

Supplement: Supplementary file 4 — Figure Source Data Appendix [file 44318_2026_747_MOESM4_ESM.zip › Appendix_Figure_S4/D/02_gray_scale_same_brightness/e1575_p1276_SPY-SNAP_zoom8_GFPplus_03-1_ch01.tif]

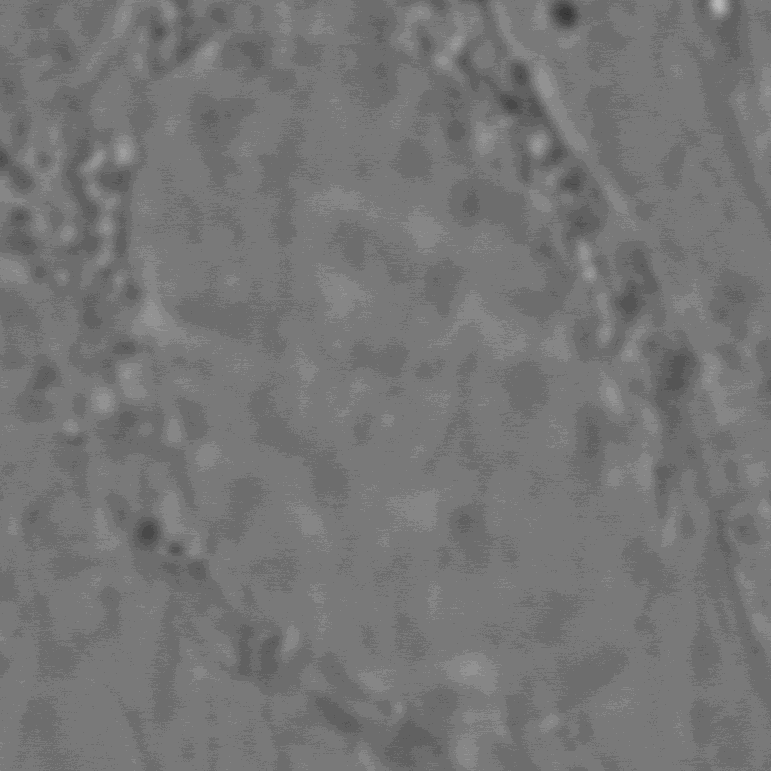

Supplement: Supplementary file 4 — Figure Source Data Appendix [file 44318_2026_747_MOESM4_ESM.zip › Appendix_Figure_S4/D/02_gray_scale_same_brightness/e1575_p1276_SPY-SNAP_zoom8_GFPminus_01-1_ch03.tif]

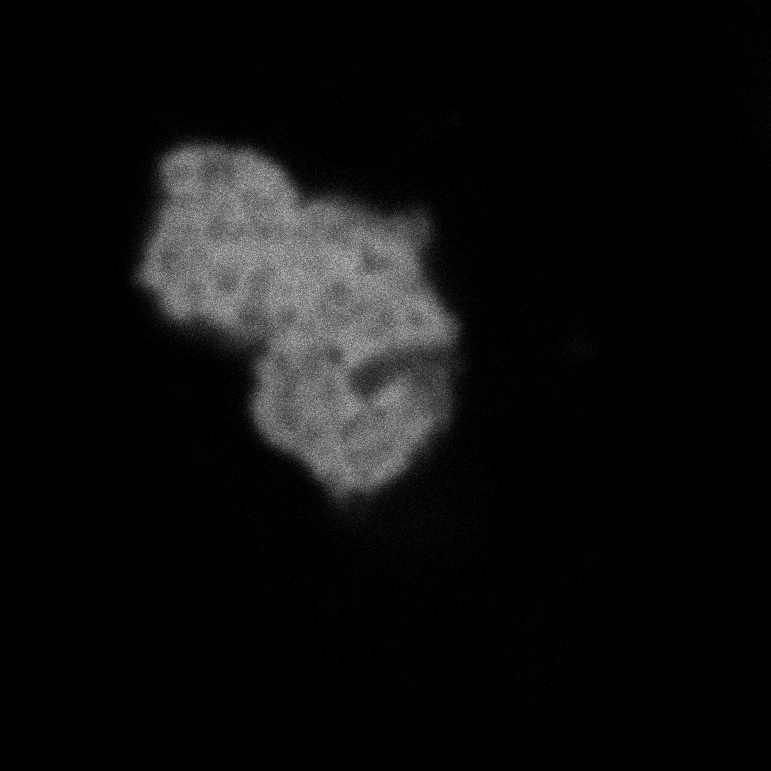

Supplement: Supplementary file 4 — Figure Source Data Appendix [file 44318_2026_747_MOESM4_ESM.zip › Appendix_Figure_S4/D/02_gray_scale_same_brightness/e1575_p1276_SPY-SNAP_zoom8_GFPminus_01-1_ch02.tif]

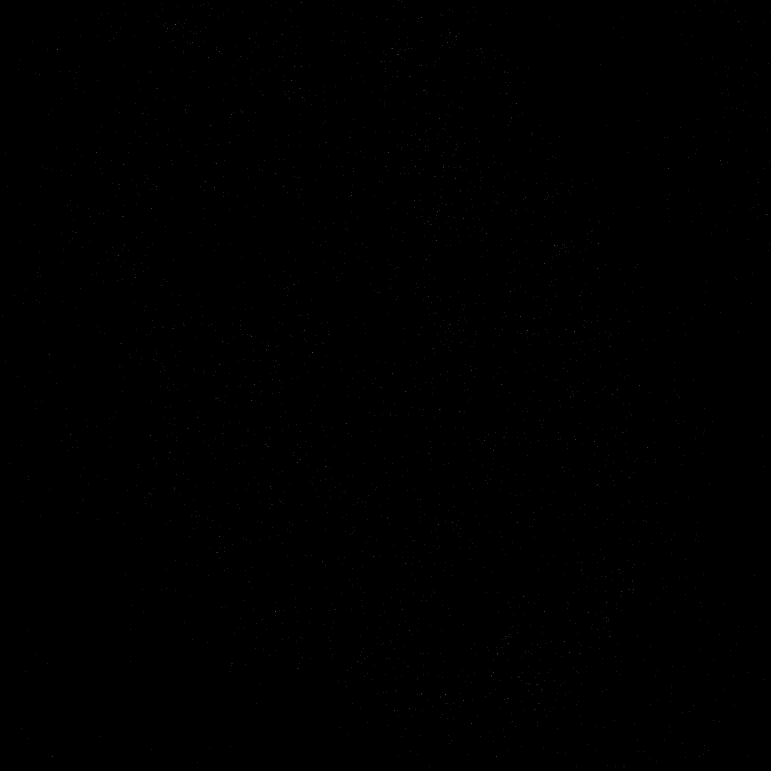

Supplement: Supplementary file 4 — Figure Source Data Appendix [file 44318_2026_747_MOESM4_ESM.zip › Appendix_Figure_S4/D/02_gray_scale_same_brightness/e1575_p1276_SPY-SNAP_zoom8_GFPminus_01-1_ch01.tif]

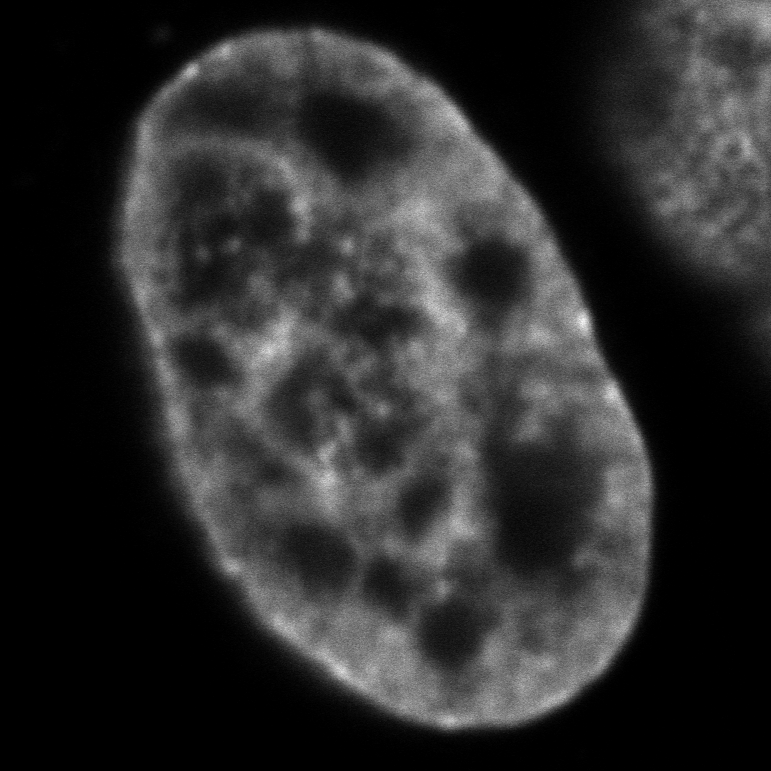

Supplement: Supplementary file 4 — Figure Source Data Appendix [file 44318_2026_747_MOESM4_ESM.zip › Appendix_Figure_S4/D/02_gray_scale_same_brightness/e1575_p1276_SPY-SNAP_zoom8_GFPminus_01-1_ch04.tif]

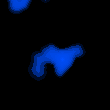

Supplement: Supplementary file 4 — Figure Source Data Appendix [file 44318_2026_747_MOESM4_ESM.zip › Appendix_Figure_S4/B/RGB/e1165_exp01_p343_DE_3_W0002_P0001_T0001_mTurq-acq1_Z_3-1.tif]

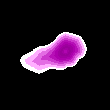

Supplement: Supplementary file 4 — Figure Source Data Appendix [file 44318_2026_747_MOESM4_ESM.zip › Appendix_Figure_S4/B/RGB/e1165_exp01_onlypei_DE_3_W0002_P0001_T0001_TM-acq1_Z_4-1.tif]

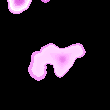

Supplement: Supplementary file 4 — Figure Source Data Appendix [file 44318_2026_747_MOESM4_ESM.zip › Appendix_Figure_S4/B/RGB/e1165_exp01_p343_DE_3_W0002_P0001_T0001_TM-acq1_Z_3-1.tif]

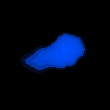

Supplement: Supplementary file 4 — Figure Source Data Appendix [file 44318_2026_747_MOESM4_ESM.zip › Appendix_Figure_S4/B/RGB/e1165_exp01_onlypei_DE_3_W0002_P0001_T0001_mTurq-acq1_Z_4-1.tif]

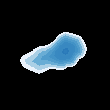

Supplement: Supplementary file 4 — Figure Source Data Appendix [file 44318_2026_747_MOESM4_ESM.zip › Appendix_Figure_S4/B/raw/e1165_exp01_onlypei_DE_3_W0002_P0001_T0001_cropped_NPM1_meanFrac.tif]

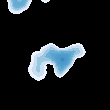

Supplement: Supplementary file 4 — Figure Source Data Appendix [file 44318_2026_747_MOESM4_ESM.zip › Appendix_Figure_S4/B/raw/e1165_exp01_p343_DE_3_W0002_P0001_T0001_cropped_NPM1_meanFrac.tif]

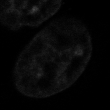

Supplement: Supplementary file 4 — Figure Source Data Appendix [file 44318_2026_747_MOESM4_ESM.zip › Appendix_Figure_S4/B/raw/e1165_exp01_p343_DE_3_W0002_P0001_T0001_cropped.tif]

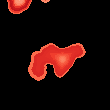

Supplement: Supplementary file 4 — Figure Source Data Appendix [file 44318_2026_747_MOESM4_ESM.zip › Appendix_Figure_S4/B/raw/e1165_exp01_p343_DE_3_W0002_P0001_T0001_cropped_DNA_meanFrac.tif]

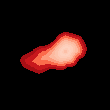

Supplement: Supplementary file 4 — Figure Source Data Appendix [file 44318_2026_747_MOESM4_ESM.zip › Appendix_Figure_S4/B/raw/e1165_exp01_onlypei_DE_3_W0002_P0001_T0001_cropped_DNA_meanFrac.tif]

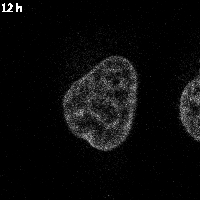

Supplement: Supplementary file 4 — Figure Source Data Appendix [file 44318_2026_747_MOESM4_ESM.zip › Appendix_Figure_S5/A/RGB/cell_23_1_e-0905_W0023_all_channels_stack_Greyscale_hyperstack.tifCh-6_SiR-DNA.tif_frame_13.tif]

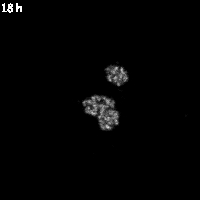

Supplement: Supplementary file 4 — Figure Source Data Appendix [file 44318_2026_747_MOESM4_ESM.zip › Appendix_Figure_S5/A/RGB/cell_23_1_e-0905_W0023_all_channels_stack_Greyscale_hyperstack.tifCh-8_FBL-TagRFP.tif_frame_19.tif]

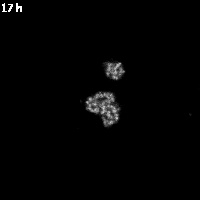

Supplement: Supplementary file 4 — Figure Source Data Appendix [file 44318_2026_747_MOESM4_ESM.zip › Appendix_Figure_S5/A/RGB/cell_23_1_e-0905_W0023_all_channels_stack_Greyscale_hyperstack.tifCh-8_FBL-TagRFP.tif_frame_18.tif]

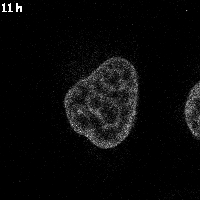

Supplement: Supplementary file 4 — Figure Source Data Appendix [file 44318_2026_747_MOESM4_ESM.zip › Appendix_Figure_S5/A/RGB/cell_23_1_e-0905_W0023_all_channels_stack_Greyscale_hyperstack.tifCh-6_SiR-DNA.tif_frame_12.tif]

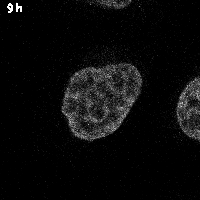

Supplement: Supplementary file 4 — Figure Source Data Appendix [file 44318_2026_747_MOESM4_ESM.zip › Appendix_Figure_S5/A/RGB/cell_23_1_e-0905_W0023_all_channels_stack_Greyscale_hyperstack.tifCh-6_SiR-DNA.tif_frame_10.tif]

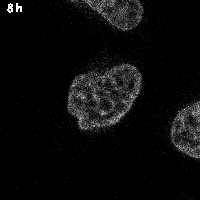

Supplement: Supplementary file 4 — Figure Source Data Appendix [file 44318_2026_747_MOESM4_ESM.zip › Appendix_Figure_S5/A/RGB/cell_23_1_e-0905_W0023_all_channels_stack_Greyscale_hyperstack.tifCh-6_SiR-DNA.tif_frame_9.tif]

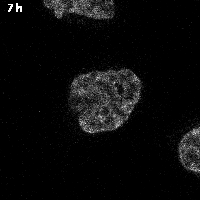

Supplement: Supplementary file 4 — Figure Source Data Appendix [file 44318_2026_747_MOESM4_ESM.zip › Appendix_Figure_S5/A/RGB/cell_23_1_e-0905_W0023_all_channels_stack_Greyscale_hyperstack.tifCh-6_SiR-DNA.tif_frame_8.tif]

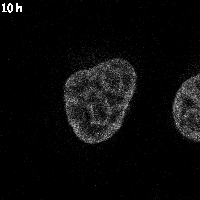

Supplement: Supplementary file 4 — Figure Source Data Appendix [file 44318_2026_747_MOESM4_ESM.zip › Appendix_Figure_S5/A/RGB/cell_23_1_e-0905_W0023_all_channels_stack_Greyscale_hyperstack.tifCh-6_SiR-DNA.tif_frame_11.tif]

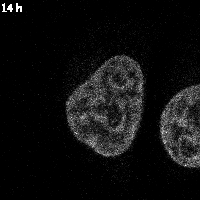

Supplement: Supplementary file 4 — Figure Source Data Appendix [file 44318_2026_747_MOESM4_ESM.zip › Appendix_Figure_S5/A/RGB/cell_23_1_e-0905_W0023_all_channels_stack_Greyscale_hyperstack.tifCh-6_SiR-DNA.tif_frame_15.tif]

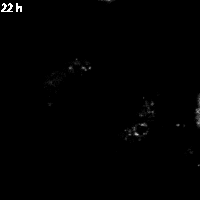

Supplement: Supplementary file 4 — Figure Source Data Appendix [file 44318_2026_747_MOESM4_ESM.zip › Appendix_Figure_S5/A/RGB/cell_23_1_e-0905_W0023_all_channels_stack_Greyscale_hyperstack.tifCh-8_FBL-TagRFP.tif_frame_23.tif]

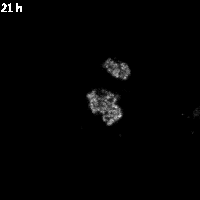

Supplement: Supplementary file 4 — Figure Source Data Appendix [file 44318_2026_747_MOESM4_ESM.zip › Appendix_Figure_S5/A/RGB/cell_23_1_e-0905_W0023_all_channels_stack_Greyscale_hyperstack.tifCh-8_FBL-TagRFP.tif_frame_22.tif]

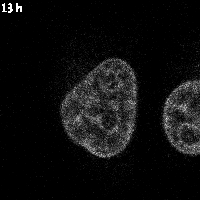

Supplement: Supplementary file 4 — Figure Source Data Appendix [file 44318_2026_747_MOESM4_ESM.zip › Appendix_Figure_S5/A/RGB/cell_23_1_e-0905_W0023_all_channels_stack_Greyscale_hyperstack.tifCh-6_SiR-DNA.tif_frame_14.tif]

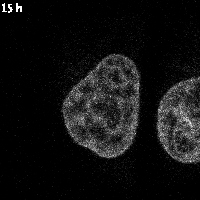

Supplement: Supplementary file 4 — Figure Source Data Appendix [file 44318_2026_747_MOESM4_ESM.zip › Appendix_Figure_S5/A/RGB/cell_23_1_e-0905_W0023_all_channels_stack_Greyscale_hyperstack.tifCh-6_SiR-DNA.tif_frame_16.tif]

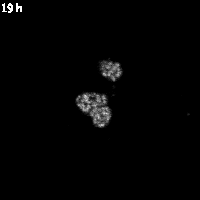

Supplement: Supplementary file 4 — Figure Source Data Appendix [file 44318_2026_747_MOESM4_ESM.zip › Appendix_Figure_S5/A/RGB/cell_23_1_e-0905_W0023_all_channels_stack_Greyscale_hyperstack.tifCh-8_FBL-TagRFP.tif_frame_20.tif]

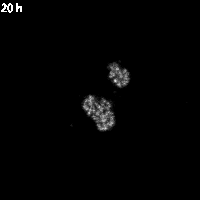

Supplement: Supplementary file 4 — Figure Source Data Appendix [file 44318_2026_747_MOESM4_ESM.zip › Appendix_Figure_S5/A/RGB/cell_23_1_e-0905_W0023_all_channels_stack_Greyscale_hyperstack.tifCh-8_FBL-TagRFP.tif_frame_21.tif]

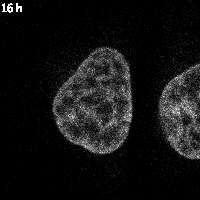

Supplement: Supplementary file 4 — Figure Source Data Appendix [file 44318_2026_747_MOESM4_ESM.zip › Appendix_Figure_S5/A/RGB/cell_23_1_e-0905_W0023_all_channels_stack_Greyscale_hyperstack.tifCh-6_SiR-DNA.tif_frame_17.tif]

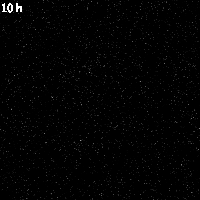

Supplement: Supplementary file 4 — Figure Source Data Appendix [file 44318_2026_747_MOESM4_ESM.zip › Appendix_Figure_S5/A/RGB/cell_23_1_e-0905_W0023_all_channels_stack_Greyscale_hyperstack.tifCh-8_EGFP-AID-Ki-67.tif_frame_11.tif]

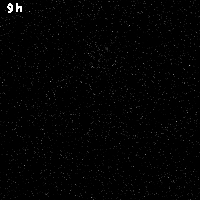

Supplement: Supplementary file 4 — Figure Source Data Appendix [file 44318_2026_747_MOESM4_ESM.zip › Appendix_Figure_S5/A/RGB/cell_23_1_e-0905_W0023_all_channels_stack_Greyscale_hyperstack.tifCh-8_EGFP-AID-Ki-67.tif_frame_10.tif]

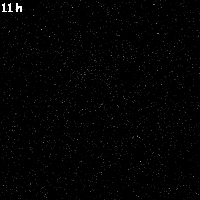

Supplement: Supplementary file 4 — Figure Source Data Appendix [file 44318_2026_747_MOESM4_ESM.zip › Appendix_Figure_S5/A/RGB/cell_23_1_e-0905_W0023_all_channels_stack_Greyscale_hyperstack.tifCh-8_EGFP-AID-Ki-67.tif_frame_12.tif]

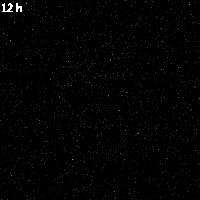

Supplement: Supplementary file 4 — Figure Source Data Appendix [file 44318_2026_747_MOESM4_ESM.zip › Appendix_Figure_S5/A/RGB/cell_23_1_e-0905_W0023_all_channels_stack_Greyscale_hyperstack.tifCh-8_EGFP-AID-Ki-67.tif_frame_13.tif]

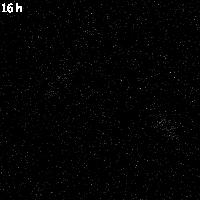

Supplement: Supplementary file 4 — Figure Source Data Appendix [file 44318_2026_747_MOESM4_ESM.zip › Appendix_Figure_S5/A/RGB/cell_23_1_e-0905_W0023_all_channels_stack_Greyscale_hyperstack.tifCh-8_EGFP-AID-Ki-67.tif_frame_17.tif]

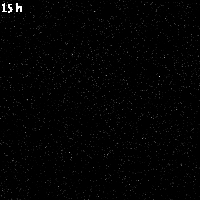

Supplement: Supplementary file 4 — Figure Source Data Appendix [file 44318_2026_747_MOESM4_ESM.zip › Appendix_Figure_S5/A/RGB/cell_23_1_e-0905_W0023_all_channels_stack_Greyscale_hyperstack.tifCh-8_EGFP-AID-Ki-67.tif_frame_16.tif]

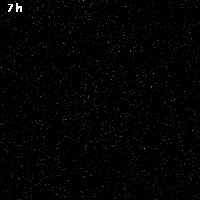

Supplement: Supplementary file 4 — Figure Source Data Appendix [file 44318_2026_747_MOESM4_ESM.zip › Appendix_Figure_S5/A/RGB/cell_23_1_e-0905_W0023_all_channels_stack_Greyscale_hyperstack.tifCh-8_EGFP-AID-Ki-67.tif_frame_8.tif]

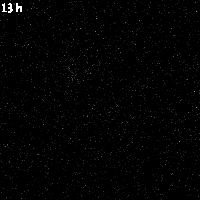

Supplement: Supplementary file 4 — Figure Source Data Appendix [file 44318_2026_747_MOESM4_ESM.zip › Appendix_Figure_S5/A/RGB/cell_23_1_e-0905_W0023_all_channels_stack_Greyscale_hyperstack.tifCh-8_EGFP-AID-Ki-67.tif_frame_14.tif]

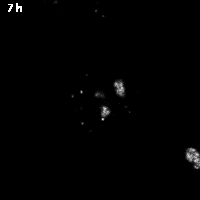

Supplement: Supplementary file 4 — Figure Source Data Appendix [file 44318_2026_747_MOESM4_ESM.zip › Appendix_Figure_S5/A/RGB/cell_23_1_e-0905_W0023_all_channels_stack_Greyscale_hyperstack.tifCh-8_FBL-TagRFP.tif_frame_8.tif]

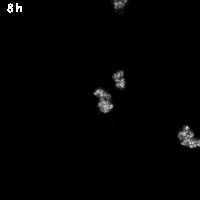

Supplement: Supplementary file 4 — Figure Source Data Appendix [file 44318_2026_747_MOESM4_ESM.zip › Appendix_Figure_S5/A/RGB/cell_23_1_e-0905_W0023_all_channels_stack_Greyscale_hyperstack.tifCh-8_FBL-TagRFP.tif_frame_9.tif]

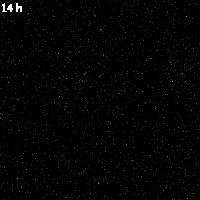

Supplement: Supplementary file 4 — Figure Source Data Appendix [file 44318_2026_747_MOESM4_ESM.zip › Appendix_Figure_S5/A/RGB/cell_23_1_e-0905_W0023_all_channels_stack_Greyscale_hyperstack.tifCh-8_EGFP-AID-Ki-67.tif_frame_15.tif]

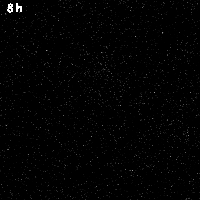

Supplement: Supplementary file 4 — Figure Source Data Appendix [file 44318_2026_747_MOESM4_ESM.zip › Appendix_Figure_S5/A/RGB/cell_23_1_e-0905_W0023_all_channels_stack_Greyscale_hyperstack.tifCh-8_EGFP-AID-Ki-67.tif_frame_9.tif]

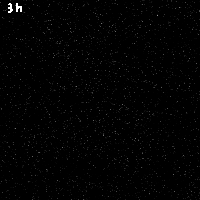

Supplement: Supplementary file 4 — Figure Source Data Appendix [file 44318_2026_747_MOESM4_ESM.zip › Appendix_Figure_S5/A/RGB/cell_23_1_e-0905_W0023_all_channels_stack_Greyscale_hyperstack.tifCh-8_EGFP-AID-Ki-67.tif_frame_4.tif]

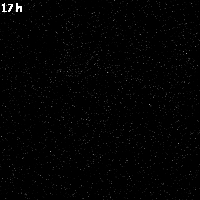

Supplement: Supplementary file 4 — Figure Source Data Appendix [file 44318_2026_747_MOESM4_ESM.zip › Appendix_Figure_S5/A/RGB/cell_23_1_e-0905_W0023_all_channels_stack_Greyscale_hyperstack.tifCh-8_EGFP-AID-Ki-67.tif_frame_18.tif]

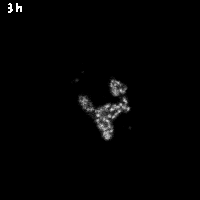

Supplement: Supplementary file 4 — Figure Source Data Appendix [file 44318_2026_747_MOESM4_ESM.zip › Appendix_Figure_S5/A/RGB/cell_23_1_e-0905_W0023_all_channels_stack_Greyscale_hyperstack.tifCh-8_FBL-TagRFP.tif_frame_4.tif]
