# Supplementary figures and images for: Ki-67 shapes the nucleolus by anchoring chromatin via its amphiphilic properties (part 3 of 5)
Source: EMBO J. 2026 Mar 24;45(9):3156–91. doi: 10.1038/s44318-026-00747-7 (PMC13144362; doi:10.1038/s44318-026-00747-7)

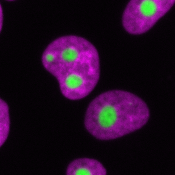

Supplement: Supplementary file 5 — Source data Fig. 1 [file 44318_2026_747_MOESM5_ESM.zip › Figure 1/C/RGB_images/e-0470_W0159--s19879--MPHOSPH10_P002--s19879--MPHOSPH10_cropped.tif]

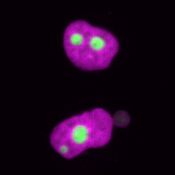

Supplement: Supplementary file 5 — Source data Fig. 1 [file 44318_2026_747_MOESM5_ESM.zip › Figure 1/C/RGB_images/e-0470_W0203--s22772--PDCD11_P004--s22772--PDCD11_cropped.tif]

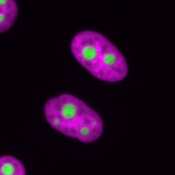

Supplement: Supplementary file 5 — Source data Fig. 1 [file 44318_2026_747_MOESM5_ESM.zip › Figure 1/C/RGB_images/e-0470_W0168--s4821--FBL_P001--s4821--FBL_cropped.tif]

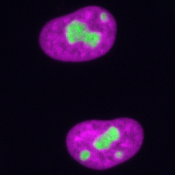

Supplement: Supplementary file 5 — Source data Fig. 1 [file 44318_2026_747_MOESM5_ESM.zip › Figure 1/C/RGB_images/e-0470_W0019--s444246--XWNeg9_P002--s444246--XWNeg9_cropped.tif (RGB).tif]

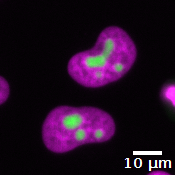

Supplement: Supplementary file 5 — Source data Fig. 1 [file 44318_2026_747_MOESM5_ESM.zip › Figure 1/C/RGB_images/e-0470_W0019--s444246--XWNeg9_P003--s444246--XWNeg9_cropped_add_scale.tif]

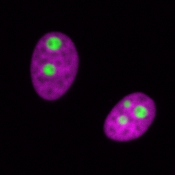

Supplement: Supplementary file 5 — Source data Fig. 1 [file 44318_2026_747_MOESM5_ESM.zip › Figure 1/C/RGB_images/e-0470_W0041--s26948--DNTTIP2_P001--s26948--DNTTIP2_cropped.tif]

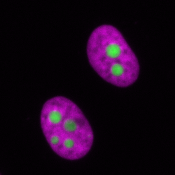

Supplement: Supplementary file 5 — Source data Fig. 1 [file 44318_2026_747_MOESM5_ESM.zip › Figure 1/C/RGB_images/e-0470_W0204--s11610--PWP2_P004--s11610--PWP2_cropped.tif]

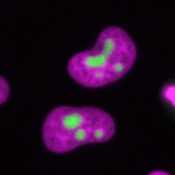

Supplement: Supplementary file 5 — Source data Fig. 1 [file 44318_2026_747_MOESM5_ESM.zip › Figure 1/C/RGB_images/e-0470_W0019--s444246--XWNeg9_P003--s444246--XWNeg9_cropped.tif]

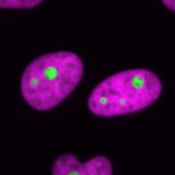

Supplement: Supplementary file 5 — Source data Fig. 1 [file 44318_2026_747_MOESM5_ESM.zip › Figure 1/C/RGB_images/e-0274_W0062--s38530--WDR75_P004--s38530--WDR75_cropped.tif]

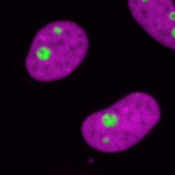

Supplement: Supplementary file 5 — Source data Fig. 1 [file 44318_2026_747_MOESM5_ESM.zip › Figure 1/C/RGB_images/e-0470_W0370--s24764--NOL11_P002--s24764--NOL11_cropped.tif]

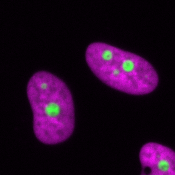

Supplement: Supplementary file 5 — Source data Fig. 1 [file 44318_2026_747_MOESM5_ESM.zip › Figure 1/C/RGB_images/e-0470_W0356--s38549--UTP15_P002--s38549--UTP15_cropped.tif]

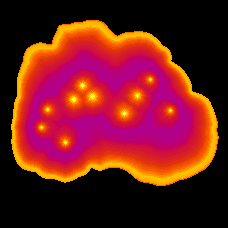

Supplement: Supplementary file 6 — Source data Fig. 2 [file 44318_2026_747_MOESM6_ESM.zip › Figure 2/G/e1170_exp09_c391_Airyscan_processed_e1170_exp09_c391_Airyscan_processed_S0_0_batch-1_DNA_menFrac_RGB_Fire.tif]

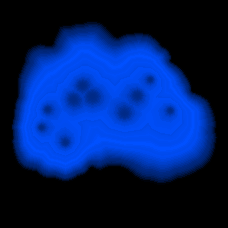

Supplement: Supplementary file 6 — Source data Fig. 2 [file 44318_2026_747_MOESM6_ESM.zip › Figure 2/G/e1170_exp09_c391_Airyscan_processed_e1170_exp09_c391_Airyscan_processed_S0_0_batch-1_NPM1_menFrac_RGB.tif]

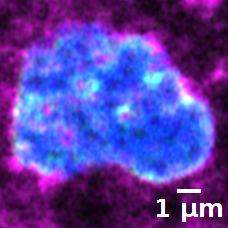

Supplement: Supplementary file 6 — Source data Fig. 2 [file 44318_2026_747_MOESM6_ESM.zip › Figure 2/G/scale.tif]

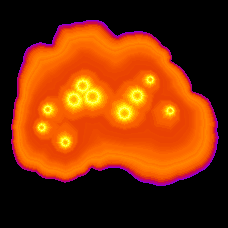

Supplement: Supplementary file 6 — Source data Fig. 2 [file 44318_2026_747_MOESM6_ESM.zip › Figure 2/G/e1170_exp09_c391_Airyscan_processed_e1170_exp09_c391_Airyscan_processed_S0_0_batch-1_Ki67_menFrac_RGB_Fire.tif]

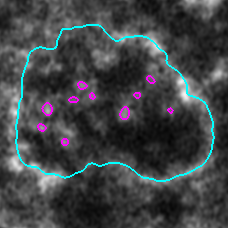

Supplement: Supplementary file 6 — Source data Fig. 2 [file 44318_2026_747_MOESM6_ESM.zip › Figure 2/G/e1170_exp09_c391_Airyscan_processed_e1170_exp09_c391_Airyscan_processed_S0_0_batch_chromatin_foci.tif]

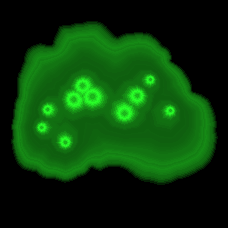

Supplement: Supplementary file 6 — Source data Fig. 2 [file 44318_2026_747_MOESM6_ESM.zip › Figure 2/G/e1170_exp09_c391_Airyscan_processed_e1170_exp09_c391_Airyscan_processed_S0_0_batch-1_Ki67_menFrac_RGB.tif]

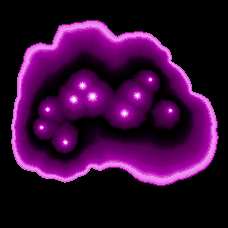

Supplement: Supplementary file 6 — Source data Fig. 2 [file 44318_2026_747_MOESM6_ESM.zip › Figure 2/G/e1170_exp09_c391_Airyscan_processed_e1170_exp09_c391_Airyscan_processed_S0_0_batch-1_DNA_menFrac_RGB.tif]

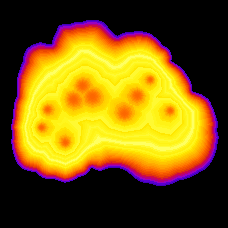

Supplement: Supplementary file 6 — Source data Fig. 2 [file 44318_2026_747_MOESM6_ESM.zip › Figure 2/G/e1170_exp09_c391_Airyscan_processed_e1170_exp09_c391_Airyscan_processed_S0_0_batch-1_NPM1_menFrac_RGB_Fire.tif]

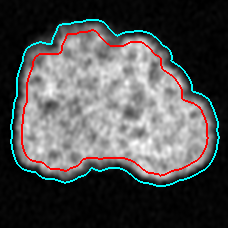

Supplement: Supplementary file 6 — Source data Fig. 2 [file 44318_2026_747_MOESM6_ESM.zip › Figure 2/B/e1170_exp09_c391_Airyscan_processed_e1170_exp09_c391_Airyscan_processed_S0_0_batch-2-1_expand_shrunk_segs.tif]

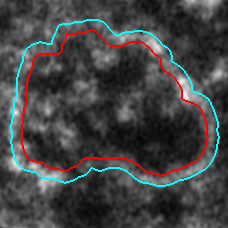

Supplement: Supplementary file 6 — Source data Fig. 2 [file 44318_2026_747_MOESM6_ESM.zip › Figure 2/B/e1170_exp09_c391_Airyscan_processed_e1170_exp09_c391_Airyscan_processed_S0_0_batch-3_expand_shrunk_segs.tif]

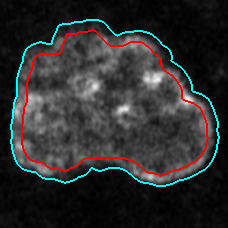

Supplement: Supplementary file 6 — Source data Fig. 2 [file 44318_2026_747_MOESM6_ESM.zip › Figure 2/B/e1170_exp09_c391_Airyscan_processed_e1170_exp09_c391_Airyscan_processed_S0_0_batch-1-1_expand_shrunk_segs.tif]

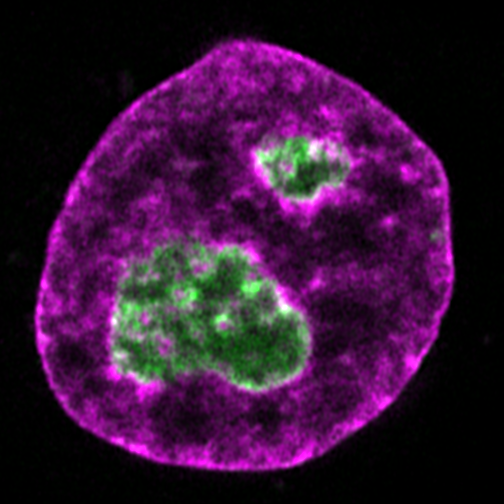

Supplement: Supplementary file 6 — Source data Fig. 2 [file 44318_2026_747_MOESM6_ESM.zip › Figure 2/A/RGB_images/e1170_exp09_c391_Airyscan_processed_e1170_exp09_c391_Airyscan_processed_S0_0_batch_Ki67_DNA.tif]

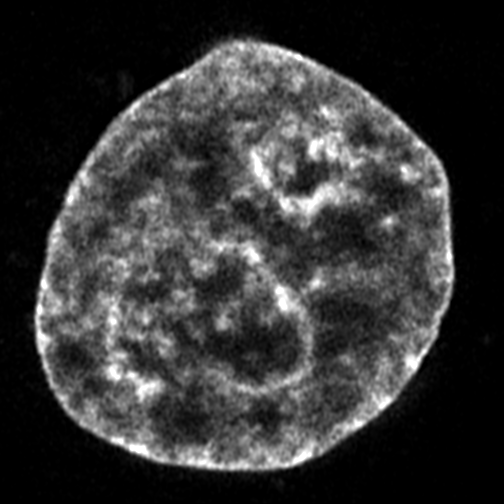

Supplement: Supplementary file 6 — Source data Fig. 2 [file 44318_2026_747_MOESM6_ESM.zip › Figure 2/A/RGB_images/e1170_exp09_c391_Airyscan_processed_e1170_exp09_c391_Airyscan_processed_S0_0_batch_ch02.tif]

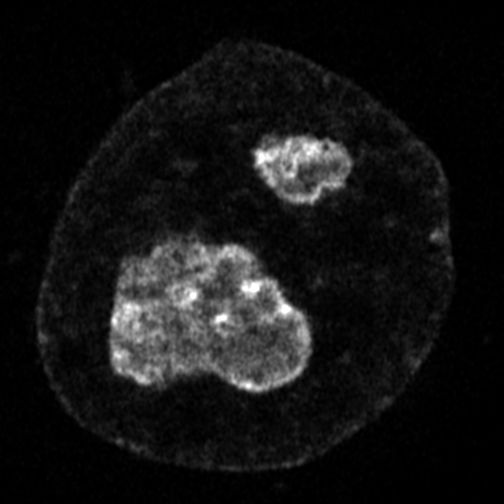

Supplement: Supplementary file 6 — Source data Fig. 2 [file 44318_2026_747_MOESM6_ESM.zip › Figure 2/A/RGB_images/e1170_exp09_c391_Airyscan_processed_e1170_exp09_c391_Airyscan_processed_S0_0_batch_ch03.tif]

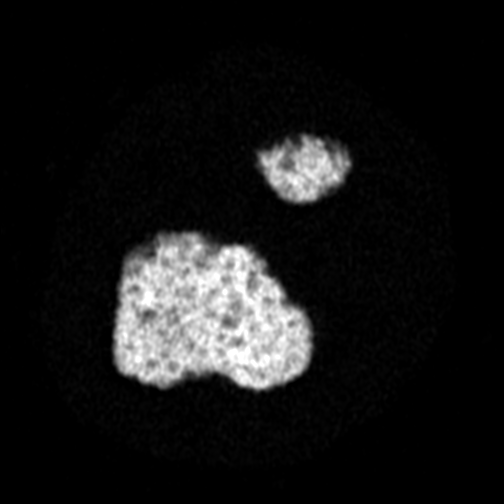

Supplement: Supplementary file 6 — Source data Fig. 2 [file 44318_2026_747_MOESM6_ESM.zip › Figure 2/A/RGB_images/e1170_exp09_c391_Airyscan_processed_e1170_exp09_c391_Airyscan_processed_S0_0_batch_ch01.tif]

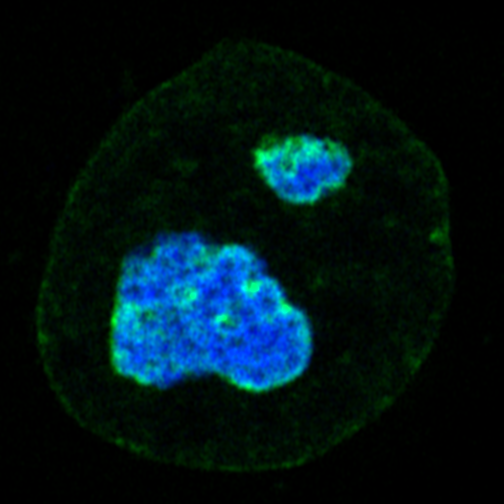

Supplement: Supplementary file 6 — Source data Fig. 2 [file 44318_2026_747_MOESM6_ESM.zip › Figure 2/A/RGB_images/e1170_exp09_c391_Airyscan_processed_e1170_exp09_c391_Airyscan_processed_S0_0_batch_Ki67_NPM1.tif]

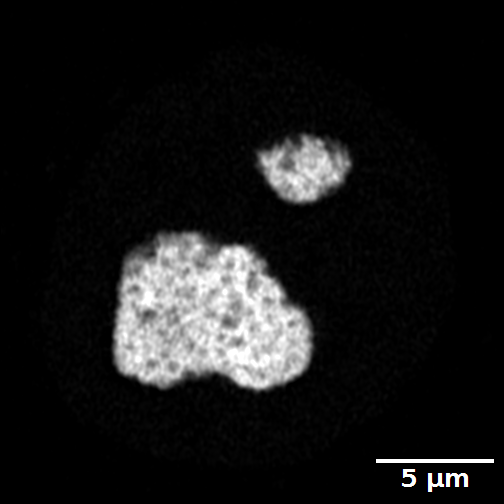

Supplement: Supplementary file 6 — Source data Fig. 2 [file 44318_2026_747_MOESM6_ESM.zip › Figure 2/A/RGB_images/e1170_exp09_c391_Airyscan_processed_e1170_exp09_c391_Airyscan_processed_S0_0_batch_ch01_scale.tif]

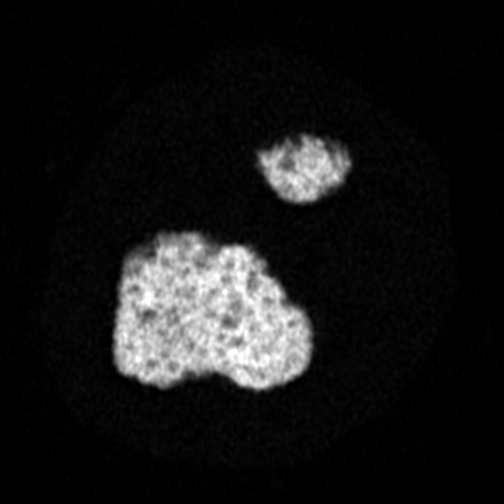

Supplement: Supplementary file 6 — Source data Fig. 2 [file 44318_2026_747_MOESM6_ESM.zip › Figure 2/A/raw_image/e1170_exp09_c391_Airyscan_processed_e1170_exp09_c391_Airyscan_processed_S0_0_batch.tif]

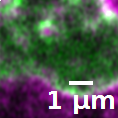

Supplement: Supplementary file 6 — Source data Fig. 2 [file 44318_2026_747_MOESM6_ESM.zip › Figure 2/F/images/e1170_exp09_c391_Airyscan_processed_e1170_exp09_c391_Airyscan_processed_S0_0_batch_zoom_Ki67_DNA_scale.tif]

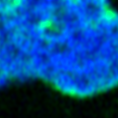

Supplement: Supplementary file 6 — Source data Fig. 2 [file 44318_2026_747_MOESM6_ESM.zip › Figure 2/F/images/e1170_exp09_c391_Airyscan_processed_e1170_exp09_c391_Airyscan_processed_S0_0_batch_zoom_Ki67_NPM1.tif]

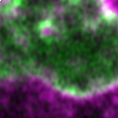

Supplement: Supplementary file 6 — Source data Fig. 2 [file 44318_2026_747_MOESM6_ESM.zip › Figure 2/F/images/e1170_exp09_c391_Airyscan_processed_e1170_exp09_c391_Airyscan_processed_S0_0_batch_zoom_Ki67_DNA.tif]

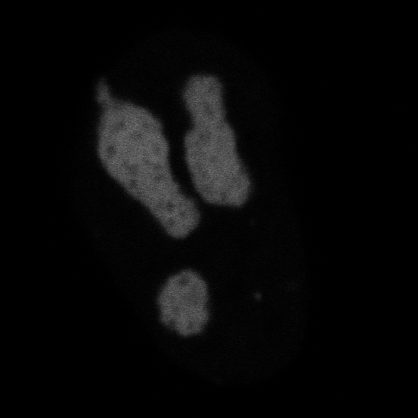

Supplement: Supplementary file 7 — Source data Fig. 3 [file 44318_2026_747_MOESM7_ESM.zip › Figure 3/A/raw_images/e1214_exp04_Control_TR1_1_W0001_P0052_T0001-1.tif]

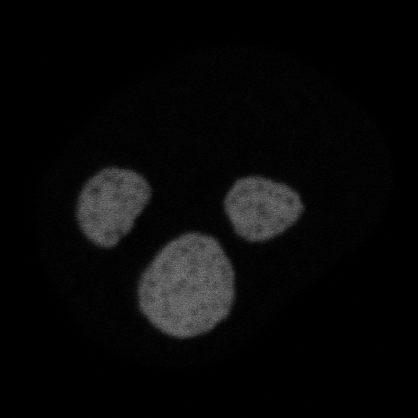

Supplement: Supplementary file 7 — Source data Fig. 3 [file 44318_2026_747_MOESM7_ESM.zip › Figure 3/A/raw_images/e1214_exp04_siKi67_1_TR1_1_W0001_P0128_T0001-1.tif]

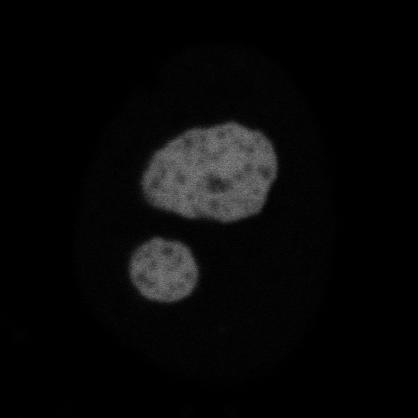

Supplement: Supplementary file 7 — Source data Fig. 3 [file 44318_2026_747_MOESM7_ESM.zip › Figure 3/A/raw_images/e1214_exp04_siKi67_2_TR1_1_W0001_P0019_T0001-1.tif]

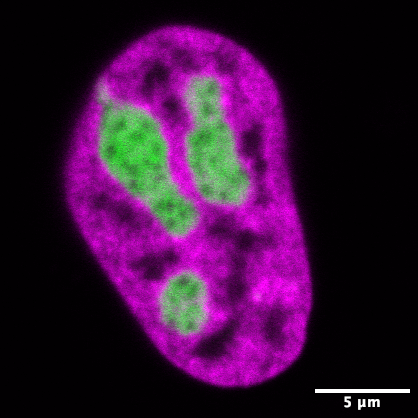

Supplement: Supplementary file 7 — Source data Fig. 3 [file 44318_2026_747_MOESM7_ESM.zip › Figure 3/A/RGB_images/scale.tif]

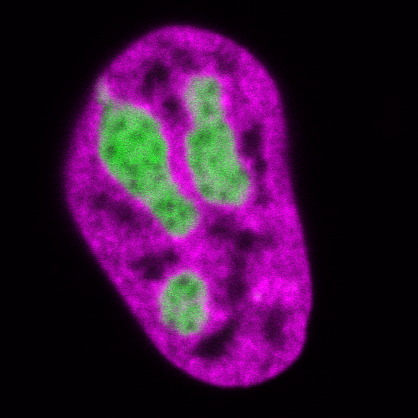

Supplement: Supplementary file 7 — Source data Fig. 3 [file 44318_2026_747_MOESM7_ESM.zip › Figure 3/A/RGB_images/e1214_exp04_Control_TR1_1_W0001_P0052_T0001-1.tif (RGB).tif]

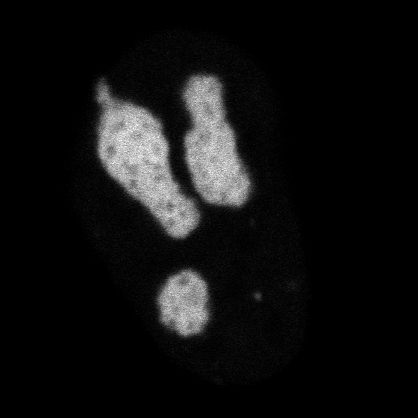

Supplement: Supplementary file 7 — Source data Fig. 3 [file 44318_2026_747_MOESM7_ESM.zip › Figure 3/A/RGB_images/e1214_exp04_Control_TR1_1_W0001_P0052_T0001-1_ch01.tif]

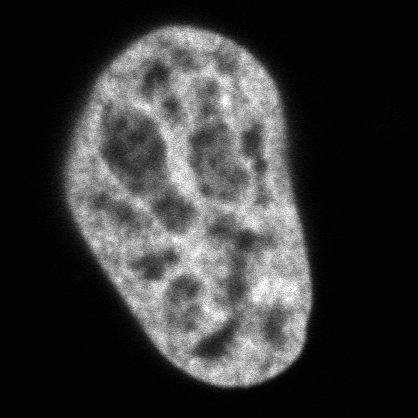

Supplement: Supplementary file 7 — Source data Fig. 3 [file 44318_2026_747_MOESM7_ESM.zip › Figure 3/A/RGB_images/e1214_exp04_Control_TR1_1_W0001_P0052_T0001-1_ch02.tif]

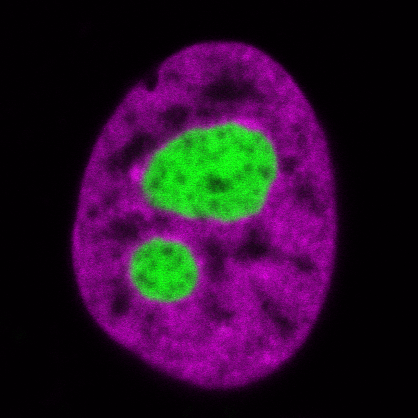

Supplement: Supplementary file 7 — Source data Fig. 3 [file 44318_2026_747_MOESM7_ESM.zip › Figure 3/A/RGB_images/e1214_exp04_siKi67_2_TR1_1_W0001_P0019_T0001-1.tif (RGB).tif]

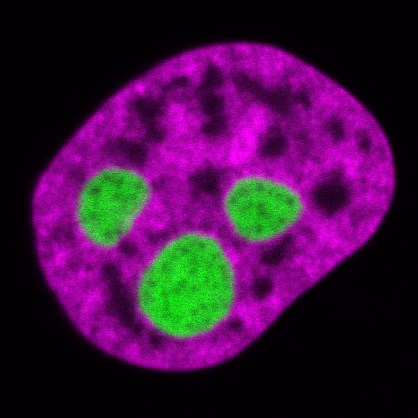

Supplement: Supplementary file 7 — Source data Fig. 3 [file 44318_2026_747_MOESM7_ESM.zip › Figure 3/A/RGB_images/e1214_exp04_siKi67_1_TR1_1_W0001_P0128_T0001-1.tif (RGB).tif]

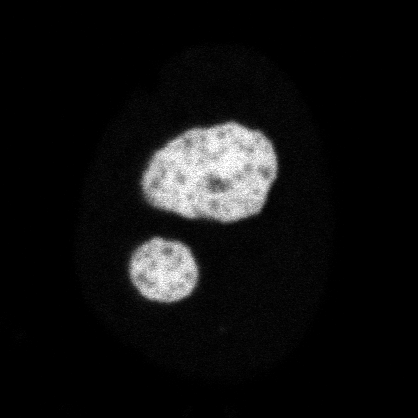

Supplement: Supplementary file 7 — Source data Fig. 3 [file 44318_2026_747_MOESM7_ESM.zip › Figure 3/A/RGB_images/e1214_exp04_siKi67_2_TR1_1_W0001_P0019_T0001-1_ch01.tif]

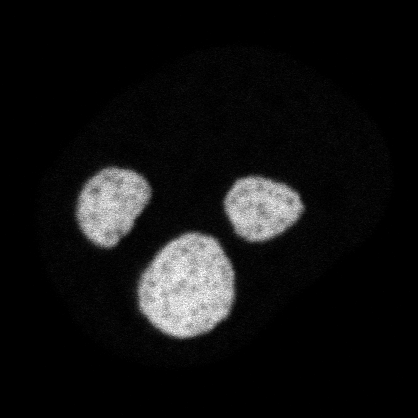

Supplement: Supplementary file 7 — Source data Fig. 3 [file 44318_2026_747_MOESM7_ESM.zip › Figure 3/A/RGB_images/e1214_exp04_siKi67_1_TR1_1_W0001_P0128_T0001-1_ch01.tif]

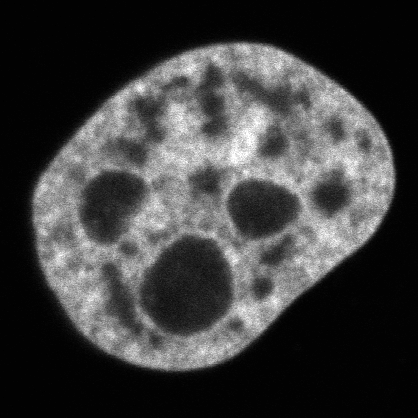

Supplement: Supplementary file 7 — Source data Fig. 3 [file 44318_2026_747_MOESM7_ESM.zip › Figure 3/A/RGB_images/e1214_exp04_siKi67_1_TR1_1_W0001_P0128_T0001-1_ch02.tif]

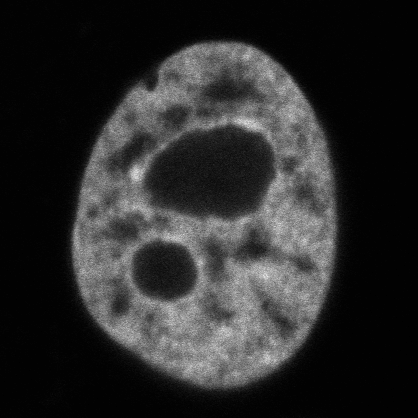

Supplement: Supplementary file 7 — Source data Fig. 3 [file 44318_2026_747_MOESM7_ESM.zip › Figure 3/A/RGB_images/e1214_exp04_siKi67_2_TR1_1_W0001_P0019_T0001-1_ch02.tif]

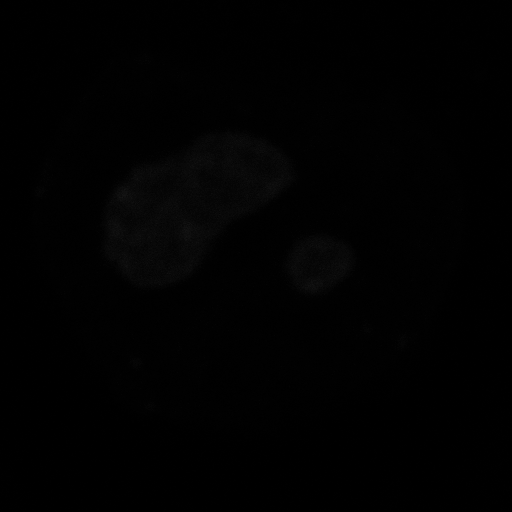

Supplement: Supplementary file 8 — Source data Fig. 4 [file 44318_2026_747_MOESM8_ESM.zip › Figure 4/A/RGB_contrast_adjusted_by_high_expression/mock_trasnfection_03_ch01.tif]

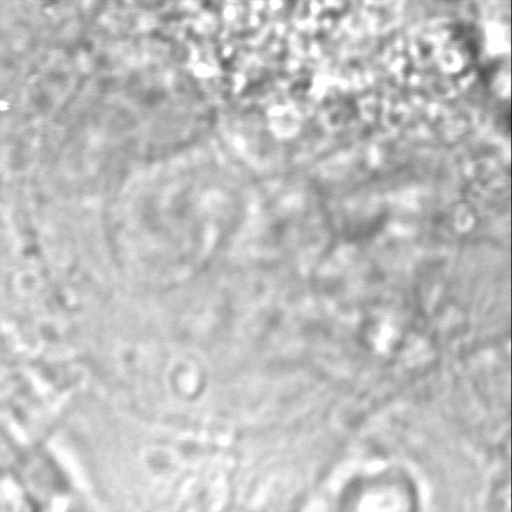

Supplement: Supplementary file 8 — Source data Fig. 4 [file 44318_2026_747_MOESM8_ESM.zip › Figure 4/A/RGB_contrast_adjusted_by_high_expression/mock_trasnfection_03_ch03.tif]

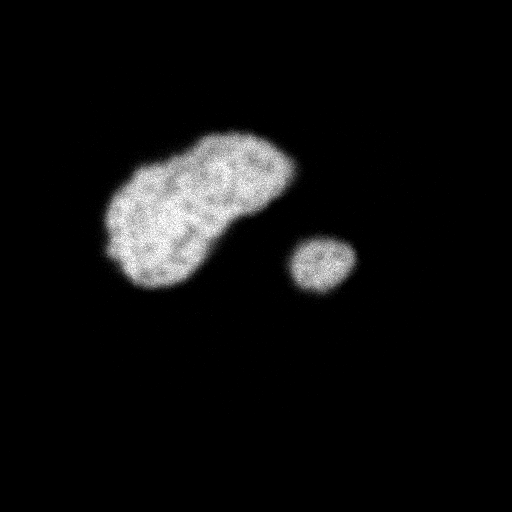

Supplement: Supplementary file 8 — Source data Fig. 4 [file 44318_2026_747_MOESM8_ESM.zip › Figure 4/A/RGB_contrast_adjusted_by_high_expression/mock_trasnfection_03_ch02.tif]

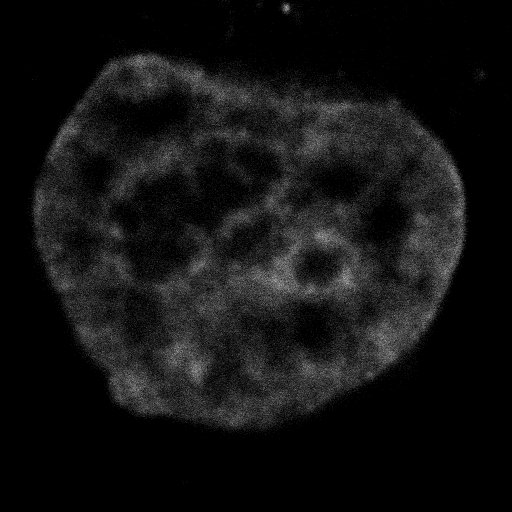

Supplement: Supplementary file 8 — Source data Fig. 4 [file 44318_2026_747_MOESM8_ESM.zip › Figure 4/A/RGB_contrast_adjusted_by_high_expression/mock_trasnfection_03_ch04.tif]

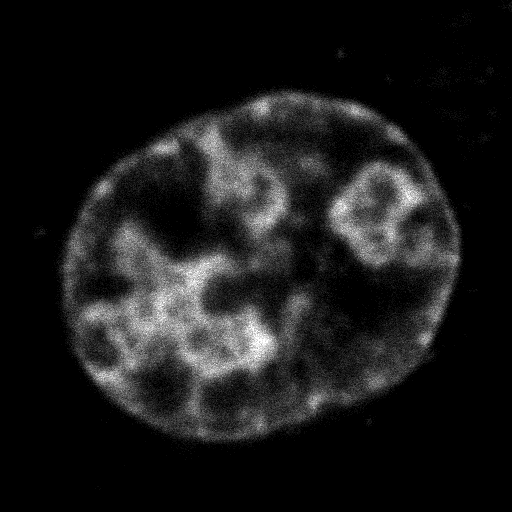

Supplement: Supplementary file 8 — Source data Fig. 4 [file 44318_2026_747_MOESM8_ESM.zip › Figure 4/A/RGB_contrast_adjusted_by_high_expression/High_overexp_06_ch04.tif]

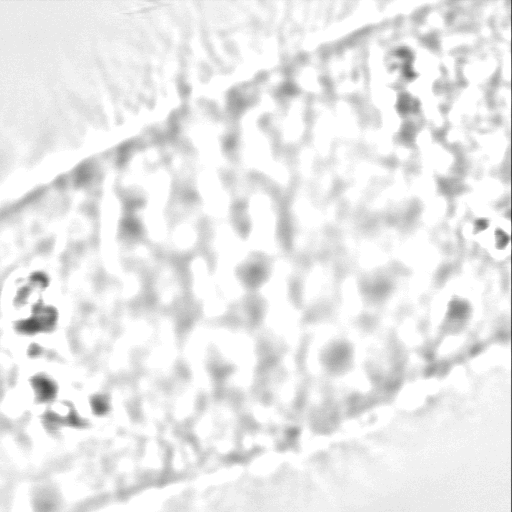

Supplement: Supplementary file 8 — Source data Fig. 4 [file 44318_2026_747_MOESM8_ESM.zip › Figure 4/A/RGB_contrast_adjusted_by_high_expression/High_overexp_06_ch03.tif]

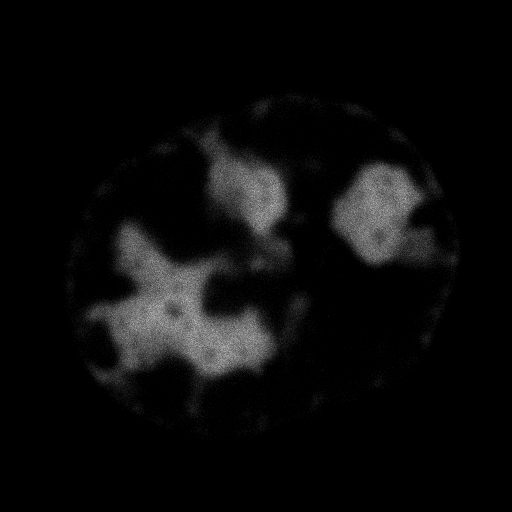

Supplement: Supplementary file 8 — Source data Fig. 4 [file 44318_2026_747_MOESM8_ESM.zip › Figure 4/A/RGB_contrast_adjusted_by_high_expression/High_overexp_06_ch02.tif]

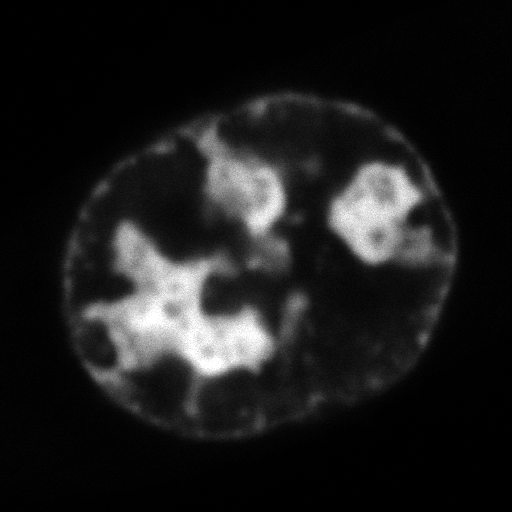

Supplement: Supplementary file 8 — Source data Fig. 4 [file 44318_2026_747_MOESM8_ESM.zip › Figure 4/A/RGB_contrast_adjusted_by_high_expression/High_overexp_06_ch01.tif]

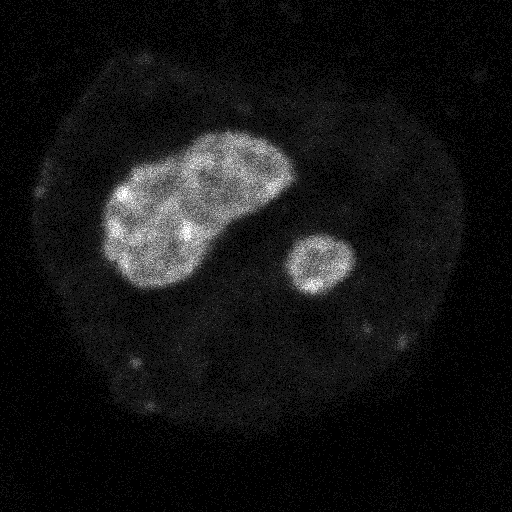

Supplement: Supplementary file 8 — Source data Fig. 4 [file 44318_2026_747_MOESM8_ESM.zip › Figure 4/A/RGB_contrast_adjusted_by_mock_expression/mock_trasnfection_03_ch01.tif]

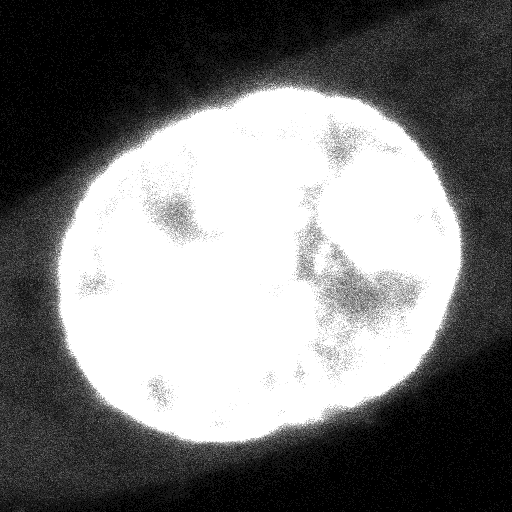

Supplement: Supplementary file 8 — Source data Fig. 4 [file 44318_2026_747_MOESM8_ESM.zip › Figure 4/A/RGB_contrast_adjusted_by_mock_expression/High_overexp_06_ch01.tif]

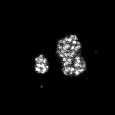

Supplement: Supplementary file 9 — Source data Fig. 5 [file 44318_2026_747_MOESM9_ESM.zip › Figure 5/A/RGB/e-0905_W0019_all_channels_stack_Greyscalecrop_cell19_1_FBL_frame12.tif]

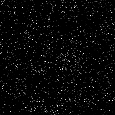

Supplement: Supplementary file 9 — Source data Fig. 5 [file 44318_2026_747_MOESM9_ESM.zip › Figure 5/A/RGB/e-0905_W0019_all_channels_stack_Greyscalecrop_cell19_1_Ki67_frame12.tif]

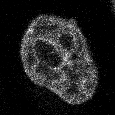

Supplement: Supplementary file 9 — Source data Fig. 5 [file 44318_2026_747_MOESM9_ESM.zip › Figure 5/A/RGB/cell_38_1_hyperstack_RGB_chromatin-9.tif]

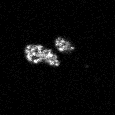

Supplement: Supplementary file 9 — Source data Fig. 5 [file 44318_2026_747_MOESM9_ESM.zip › Figure 5/A/RGB/cell_38_1_hyperstack_RGB_FBL-15.tif]

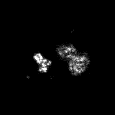

Supplement: Supplementary file 9 — Source data Fig. 5 [file 44318_2026_747_MOESM9_ESM.zip › Figure 5/A/RGB/e-0905_W0019_all_channels_stack_Greyscalecrop_cell19_1_FBL_frame1.tif]

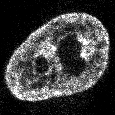

Supplement: Supplementary file 9 — Source data Fig. 5 [file 44318_2026_747_MOESM9_ESM.zip › Figure 5/A/RGB/e-0905_W0019_all_channels_stack_Greyscalecrop_cell19_1_DNA_frame12.tif]

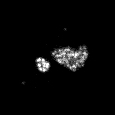

Supplement: Supplementary file 9 — Source data Fig. 5 [file 44318_2026_747_MOESM9_ESM.zip › Figure 5/A/RGB/e-0905_W0019_all_channels_stack_Greyscalecrop_cell19_1_FBL_frame3.tif]

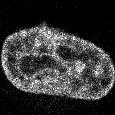

Supplement: Supplementary file 9 — Source data Fig. 5 [file 44318_2026_747_MOESM9_ESM.zip › Figure 5/A/RGB/cell_38_1_hyperstack_RGB_chromatin-18.tif]

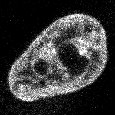

Supplement: Supplementary file 9 — Source data Fig. 5 [file 44318_2026_747_MOESM9_ESM.zip › Figure 5/A/RGB/e-0905_W0019_all_channels_stack_Greyscalecrop_cell19_1_DNA_frame9.tif]

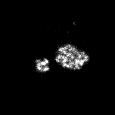

Supplement: Supplementary file 9 — Source data Fig. 5 [file 44318_2026_747_MOESM9_ESM.zip › Figure 5/A/RGB/e-0905_W0019_all_channels_stack_Greyscalecrop_cell19_1_FBL_frame6.tif]

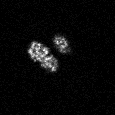

Supplement: Supplementary file 9 — Source data Fig. 5 [file 44318_2026_747_MOESM9_ESM.zip › Figure 5/A/RGB/cell_38_1_hyperstack_RGB_FBL-12.tif]

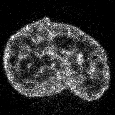

Supplement: Supplementary file 9 — Source data Fig. 5 [file 44318_2026_747_MOESM9_ESM.zip › Figure 5/A/RGB/cell_38_1_hyperstack_RGB_chromatin-23.tif]

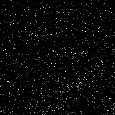

Supplement: Supplementary file 9 — Source data Fig. 5 [file 44318_2026_747_MOESM9_ESM.zip › Figure 5/A/RGB/e-0905_W0019_all_channels_stack_Greyscalecrop_cell19_1_Ki-67_frame3.tif]

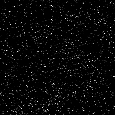

Supplement: Supplementary file 9 — Source data Fig. 5 [file 44318_2026_747_MOESM9_ESM.zip › Figure 5/A/RGB/e-0905_W0019_all_channels_stack_Greyscalecrop_cell19_1_Ki67_frame9.tif]

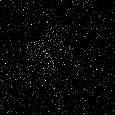

Supplement: Supplementary file 9 — Source data Fig. 5 [file 44318_2026_747_MOESM9_ESM.zip › Figure 5/A/RGB/cell_38_1_hyperstack_RGB_Ki67-12.tif]

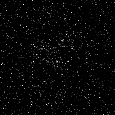

Supplement: Supplementary file 9 — Source data Fig. 5 [file 44318_2026_747_MOESM9_ESM.zip › Figure 5/A/RGB/cell_38_1_hyperstack_RGB_Ki67-9.tif]

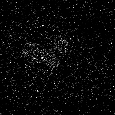

Supplement: Supplementary file 9 — Source data Fig. 5 [file 44318_2026_747_MOESM9_ESM.zip › Figure 5/A/RGB/cell_38_1_hyperstack_RGB_Ki67-15.tif]

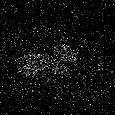

Supplement: Supplementary file 9 — Source data Fig. 5 [file 44318_2026_747_MOESM9_ESM.zip › Figure 5/A/RGB/cell_38_1_hyperstack_RGB_Ki67-18.tif]

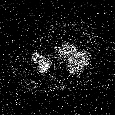

Supplement: Supplementary file 9 — Source data Fig. 5 [file 44318_2026_747_MOESM9_ESM.zip › Figure 5/A/RGB/e-0905_W0019_all_channels_stack_Greyscalecrop_cell19_1_Ki67_frame1.tif]

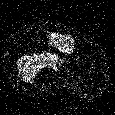

Supplement: Supplementary file 9 — Source data Fig. 5 [file 44318_2026_747_MOESM9_ESM.zip › Figure 5/A/RGB/cell_38_1_hyperstack_RGB_Ki67-23.tif]

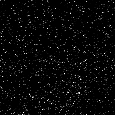

Supplement: Supplementary file 9 — Source data Fig. 5 [file 44318_2026_747_MOESM9_ESM.zip › Figure 5/A/RGB/e-0905_W0019_all_channels_stack_Greyscalecrop_cell19_1_Ki67_frame6.tif]

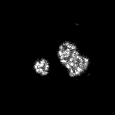

Supplement: Supplementary file 9 — Source data Fig. 5 [file 44318_2026_747_MOESM9_ESM.zip › Figure 5/A/RGB/e-0905_W0019_all_channels_stack_Greyscalecrop_cell19_1_FBL_frame9.tif]

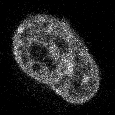

Supplement: Supplementary file 9 — Source data Fig. 5 [file 44318_2026_747_MOESM9_ESM.zip › Figure 5/A/RGB/cell_38_1_hyperstack_RGB_chromatin-12.tif]

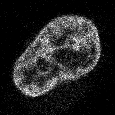

Supplement: Supplementary file 9 — Source data Fig. 5 [file 44318_2026_747_MOESM9_ESM.zip › Figure 5/A/RGB/e-0905_W0019_all_channels_stack_Greyscalecrop_cell19_1_DNA_frame3.tif]

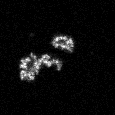

Supplement: Supplementary file 9 — Source data Fig. 5 [file 44318_2026_747_MOESM9_ESM.zip › Figure 5/A/RGB/cell_38_1_hyperstack_RGB_FBL-23.tif]

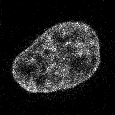

Supplement: Supplementary file 9 — Source data Fig. 5 [file 44318_2026_747_MOESM9_ESM.zip › Figure 5/A/RGB/e-0905_W0019_all_channels_stack_Greyscalecrop_cell19_1_DNA_frame1.tif]

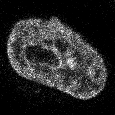

Supplement: Supplementary file 9 — Source data Fig. 5 [file 44318_2026_747_MOESM9_ESM.zip › Figure 5/A/RGB/cell_38_1_hyperstack_RGB_chromatin-15.tif]

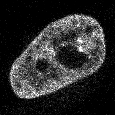

Supplement: Supplementary file 9 — Source data Fig. 5 [file 44318_2026_747_MOESM9_ESM.zip › Figure 5/A/RGB/e-0905_W0019_all_channels_stack_Greyscalecrop_cell19_1_DNA_frame6.tif]

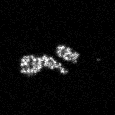

Supplement: Supplementary file 9 — Source data Fig. 5 [file 44318_2026_747_MOESM9_ESM.zip › Figure 5/A/RGB/cell_38_1_hyperstack_RGB_FBL-18.tif]

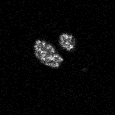

Supplement: Supplementary file 9 — Source data Fig. 5 [file 44318_2026_747_MOESM9_ESM.zip › Figure 5/A/RGB/cell_38_1_hyperstack_RGB_FBL-9.tif]

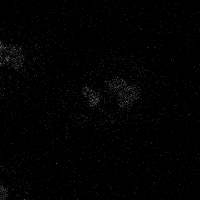

Supplement: Supplementary file 9 — Source data Fig. 5 [file 44318_2026_747_MOESM9_ESM.zip › Figure 5/A/raw_image/e-0905_W0019_Ki67.tif]

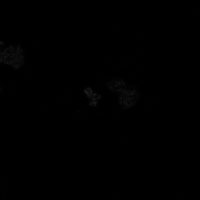

Supplement: Supplementary file 9 — Source data Fig. 5 [file 44318_2026_747_MOESM9_ESM.zip › Figure 5/A/raw_image/e-0905_W0019_FBL.tif]

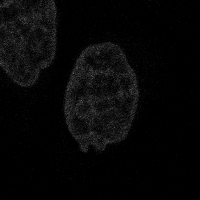

Supplement: Supplementary file 9 — Source data Fig. 5 [file 44318_2026_747_MOESM9_ESM.zip › Figure 5/A/raw_image/cell_38_1_chromatin.tif]

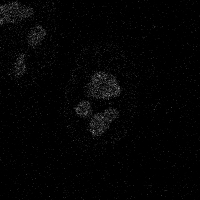

Supplement: Supplementary file 9 — Source data Fig. 5 [file 44318_2026_747_MOESM9_ESM.zip › Figure 5/A/raw_image/cell_38_1_Ki67.tif]

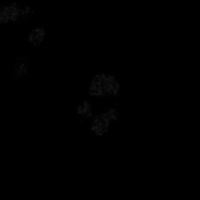

Supplement: Supplementary file 9 — Source data Fig. 5 [file 44318_2026_747_MOESM9_ESM.zip › Figure 5/A/raw_image/cell_38_1_FBL.tif]

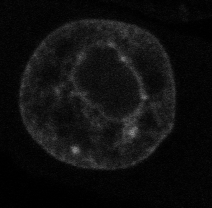

Supplement: Supplementary file 10 — Source data Fig. 6 [file 44318_2026_747_MOESM10_ESM.zip › Figure 6/B/raw_images/e-1098_c-349_p-483_DE_3_W0006_02.tif]

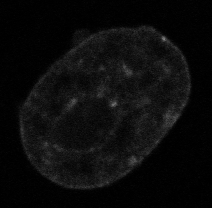

Supplement: Supplementary file 10 — Source data Fig. 6 [file 44318_2026_747_MOESM10_ESM.zip › Figure 6/B/raw_images/e-1098-exp06_c-349_p-665_DE_3_W0005_P0001_T0001.tif]

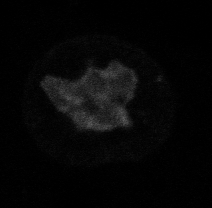

Supplement: Supplementary file 10 — Source data Fig. 6 [file 44318_2026_747_MOESM10_ESM.zip › Figure 6/B/raw_images/e-1098_c-349_p-343_DE_3_W0007_03.tif]

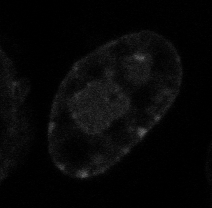

Supplement: Supplementary file 10 — Source data Fig. 6 [file 44318_2026_747_MOESM10_ESM.zip › Figure 6/B/raw_images/e-1098_c-349_p-819_DE_3_W0004_02.tif]

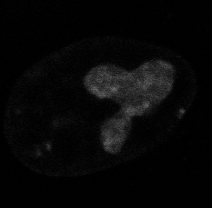

Supplement: Supplementary file 10 — Source data Fig. 6 [file 44318_2026_747_MOESM10_ESM.zip › Figure 6/B/raw_images/e-1098_c-349_p-818_DE_3_W0006_P0001_T0001.tif]

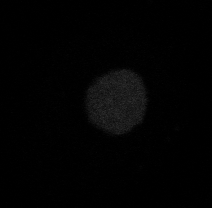

Supplement: Supplementary file 10 — Source data Fig. 6 [file 44318_2026_747_MOESM10_ESM.zip › Figure 6/B/raw_images/e-1098_c-349_p-428_DE_3_W0003_01.tif]

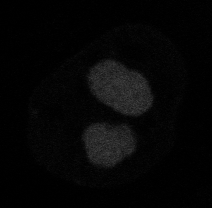

Supplement: Supplementary file 10 — Source data Fig. 6 [file 44318_2026_747_MOESM10_ESM.zip › Figure 6/B/raw_images/e-1098_c-349_p-607_DE_3_W0005_03.tif]

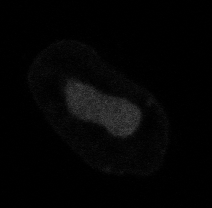

Supplement: Supplementary file 10 — Source data Fig. 6 [file 44318_2026_747_MOESM10_ESM.zip › Figure 6/B/raw_images/e-1098_c-349_p-757_DE_3_W0003_03.tif]

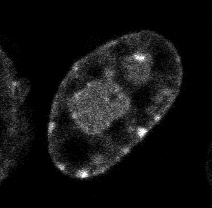

Supplement: Supplementary file 10 — Source data Fig. 6 [file 44318_2026_747_MOESM10_ESM.zip › Figure 6/B/RGB/e-1098_c-349_p-819_DE_3_W0004_0201.tif]

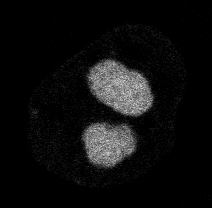

Supplement: Supplementary file 10 — Source data Fig. 6 [file 44318_2026_747_MOESM10_ESM.zip › Figure 6/B/RGB/e-1098_c-349_p-607_DE_3_W0005_0301.tif]
