# Supplementary figures and images for: Ki-67 shapes the nucleolus by anchoring chromatin via its amphiphilic properties (part 4 of 5)
Source: EMBO J. 2026 Mar 24;45(9):3156–91. doi: 10.1038/s44318-026-00747-7 (PMC13144362; doi:10.1038/s44318-026-00747-7)

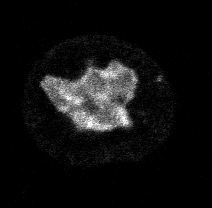

Supplement: Supplementary file 10 — Source data Fig. 6 [file 44318_2026_747_MOESM10_ESM.zip › Figure 6/B/RGB/e-1098_c-349_p-343_DE_3_W0007_0301.tif]

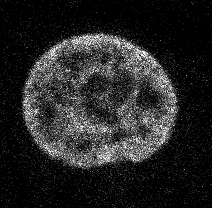

Supplement: Supplementary file 10 — Source data Fig. 6 [file 44318_2026_747_MOESM10_ESM.zip › Figure 6/B/RGB/e-1098_c-349_p-343_DE_3_W0007_0303.tif]

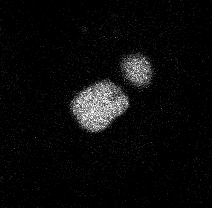

Supplement: Supplementary file 10 — Source data Fig. 6 [file 44318_2026_747_MOESM10_ESM.zip › Figure 6/B/RGB/e-1098_c-349_p-819_DE_3_W0004_0202.tif]

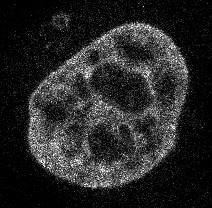

Supplement: Supplementary file 10 — Source data Fig. 6 [file 44318_2026_747_MOESM10_ESM.zip › Figure 6/B/RGB/e-1098_c-349_p-607_DE_3_W0005_0303.tif]

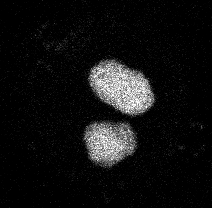

Supplement: Supplementary file 10 — Source data Fig. 6 [file 44318_2026_747_MOESM10_ESM.zip › Figure 6/B/RGB/e-1098_c-349_p-607_DE_3_W0005_0302.tif]

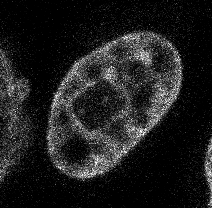

Supplement: Supplementary file 10 — Source data Fig. 6 [file 44318_2026_747_MOESM10_ESM.zip › Figure 6/B/RGB/e-1098_c-349_p-819_DE_3_W0004_0203.tif]

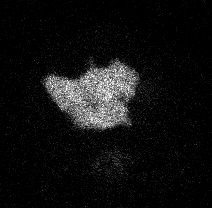

Supplement: Supplementary file 10 — Source data Fig. 6 [file 44318_2026_747_MOESM10_ESM.zip › Figure 6/B/RGB/e-1098_c-349_p-343_DE_3_W0007_0302.tif]

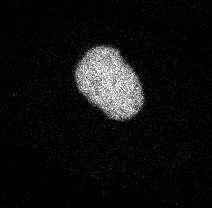

Supplement: Supplementary file 10 — Source data Fig. 6 [file 44318_2026_747_MOESM10_ESM.zip › Figure 6/B/RGB/e-1098_c-349_p-483_DE_3_W0006_0202.tif]

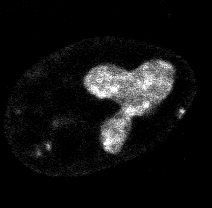

Supplement: Supplementary file 10 — Source data Fig. 6 [file 44318_2026_747_MOESM10_ESM.zip › Figure 6/B/RGB/e-1098_c-349_p-818_DE_3_W0006_P0001_T000101.tif]

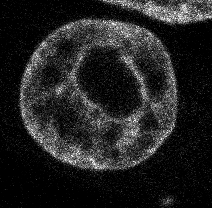

Supplement: Supplementary file 10 — Source data Fig. 6 [file 44318_2026_747_MOESM10_ESM.zip › Figure 6/B/RGB/e-1098_c-349_p-483_DE_3_W0006_0203.tif]

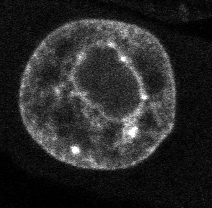

Supplement: Supplementary file 10 — Source data Fig. 6 [file 44318_2026_747_MOESM10_ESM.zip › Figure 6/B/RGB/e-1098_c-349_p-483_DE_3_W0006_0201.tif]

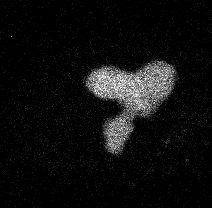

Supplement: Supplementary file 10 — Source data Fig. 6 [file 44318_2026_747_MOESM10_ESM.zip › Figure 6/B/RGB/e-1098_c-349_p-818_DE_3_W0006_P0001_T000102.tif]

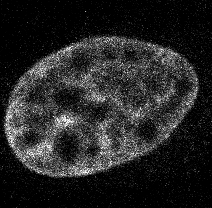

Supplement: Supplementary file 10 — Source data Fig. 6 [file 44318_2026_747_MOESM10_ESM.zip › Figure 6/B/RGB/e-1098_c-349_p-818_DE_3_W0006_P0001_T000103.tif]

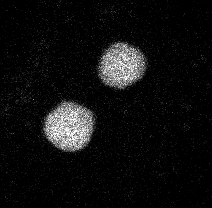

Supplement: Supplementary file 10 — Source data Fig. 6 [file 44318_2026_747_MOESM10_ESM.zip › Figure 6/B/RGB/e-1098_c-349_onlypei_DE_3_W0002_P0001_T000102.tif]

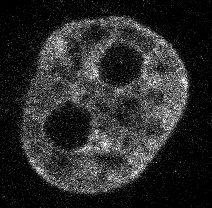

Supplement: Supplementary file 10 — Source data Fig. 6 [file 44318_2026_747_MOESM10_ESM.zip › Figure 6/B/RGB/e-1098_c-349_onlypei_DE_3_W0002_P0001_T000103.tif]

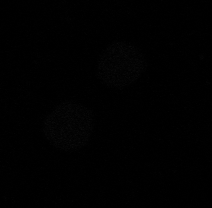

Supplement: Supplementary file 10 — Source data Fig. 6 [file 44318_2026_747_MOESM10_ESM.zip › Figure 6/B/RGB/e-1098_c-349_onlypei_DE_3_W0002_P0001_T000101.tif]

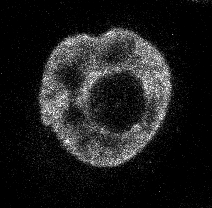

Supplement: Supplementary file 10 — Source data Fig. 6 [file 44318_2026_747_MOESM10_ESM.zip › Figure 6/B/RGB/e-1098_c-349_p-428_DE_3_W0003_0103.tif]

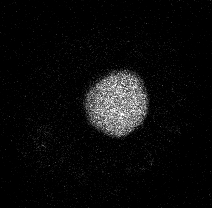

Supplement: Supplementary file 10 — Source data Fig. 6 [file 44318_2026_747_MOESM10_ESM.zip › Figure 6/B/RGB/e-1098_c-349_p-428_DE_3_W0003_0102.tif]

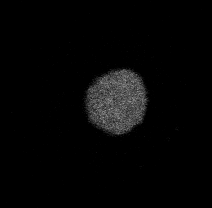

Supplement: Supplementary file 10 — Source data Fig. 6 [file 44318_2026_747_MOESM10_ESM.zip › Figure 6/B/RGB/e-1098_c-349_p-428_DE_3_W0003_0101.tif]

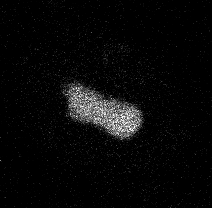

Supplement: Supplementary file 10 — Source data Fig. 6 [file 44318_2026_747_MOESM10_ESM.zip › Figure 6/B/RGB/e-1098_c-349_p-757_DE_3_W0003_0302.tif]

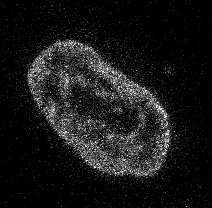

Supplement: Supplementary file 10 — Source data Fig. 6 [file 44318_2026_747_MOESM10_ESM.zip › Figure 6/B/RGB/e-1098_c-349_p-757_DE_3_W0003_0303.tif]

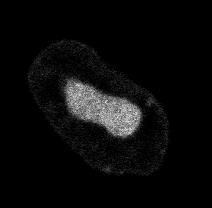

Supplement: Supplementary file 10 — Source data Fig. 6 [file 44318_2026_747_MOESM10_ESM.zip › Figure 6/B/RGB/e-1098_c-349_p-757_DE_3_W0003_0301.tif]

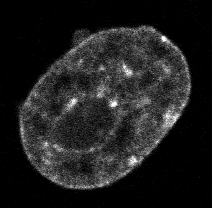

Supplement: Supplementary file 10 — Source data Fig. 6 [file 44318_2026_747_MOESM10_ESM.zip › Figure 6/B/RGB/e-1098-exp06_c-349_p-665_DE_3_W0005_P0001_T000101.tif]

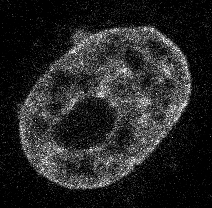

Supplement: Supplementary file 10 — Source data Fig. 6 [file 44318_2026_747_MOESM10_ESM.zip › Figure 6/B/RGB/e-1098-exp06_c-349_p-665_DE_3_W0005_P0001_T000103.tif]

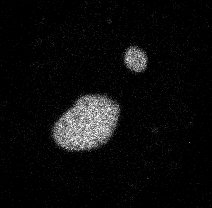

Supplement: Supplementary file 10 — Source data Fig. 6 [file 44318_2026_747_MOESM10_ESM.zip › Figure 6/B/RGB/e-1098-exp06_c-349_p-665_DE_3_W0005_P0001_T000102.tif]

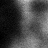

Supplement: Supplementary file 11 — Source data Fig. 7 [file 44318_2026_747_MOESM11_ESM.zip › Figure 7/A/RGB/e-0842_well-A3_p-219_cell-14_zoom-35_registered_line_1-1-1_ch01.tif]

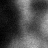

Supplement: Supplementary file 11 — Source data Fig. 7 [file 44318_2026_747_MOESM11_ESM.zip › Figure 7/A/RGB/e-0842_well-A3_p-219_cell-14_zoom-35_registered_line_1-1-1_ch02.tif]

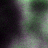

Supplement: Supplementary file 11 — Source data Fig. 7 [file 44318_2026_747_MOESM11_ESM.zip › Figure 7/A/RGB/e-0842_well-A3_p-219_cell-14_zoom-35_registered_line_1-1-1.tif (RGB).tif]

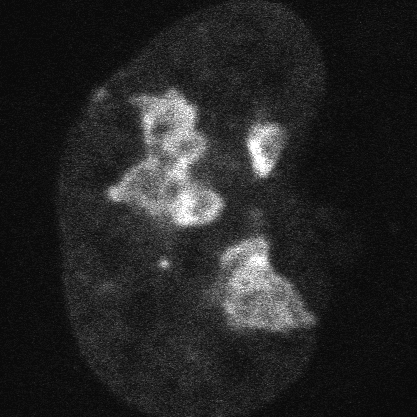

Supplement: Supplementary file 11 — Source data Fig. 7 [file 44318_2026_747_MOESM11_ESM.zip › Figure 7/A/RGB/e-0842_well-A3_p-219_cell-14_zoom-8-1_crop.tif]

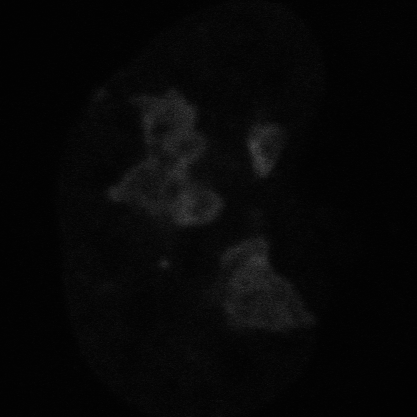

Supplement: Supplementary file 11 — Source data Fig. 7 [file 44318_2026_747_MOESM11_ESM.zip › Figure 7/A/raw/e-0842_well-A3_p-219_cell-14_zoom-8_slice4.tif]

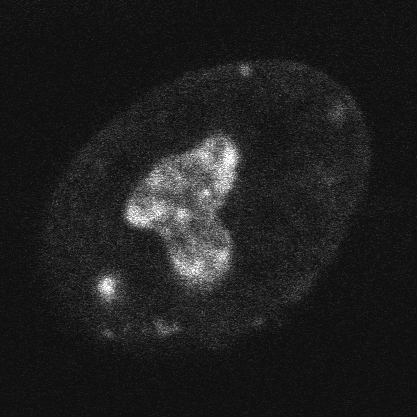

Supplement: Supplementary file 11 — Source data Fig. 7 [file 44318_2026_747_MOESM11_ESM.zip › Figure 7/C/RGB/e-0842_well-B3_p-249_cell-16_zoom-8-1_crop.tif]

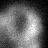

Supplement: Supplementary file 11 — Source data Fig. 7 [file 44318_2026_747_MOESM11_ESM.zip › Figure 7/C/RGB/e-0842_well-B3_p-249_cell-16_zoom-35_registered_line_2-1-1_ch01.tif]

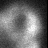

Supplement: Supplementary file 11 — Source data Fig. 7 [file 44318_2026_747_MOESM11_ESM.zip › Figure 7/C/RGB/e-0842_well-B3_p-249_cell-16_zoom-35_registered_line_2-1-1_ch02.tif]

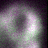

Supplement: Supplementary file 11 — Source data Fig. 7 [file 44318_2026_747_MOESM11_ESM.zip › Figure 7/C/RGB/e-0842_well-B3_p-249_cell-16_zoom-35_registered_line_2-1-1.tif (RGB).tif]

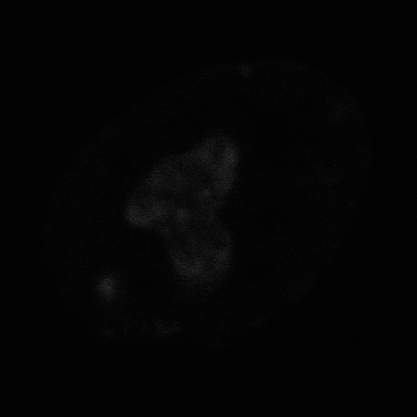

Supplement: Supplementary file 11 — Source data Fig. 7 [file 44318_2026_747_MOESM11_ESM.zip › Figure 7/C/raw/e-0842_well-B3_p-249_cell-16_zoom-8-1_slice6.tif]

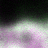

Supplement: Supplementary file 11 — Source data Fig. 7 [file 44318_2026_747_MOESM11_ESM.zip › Figure 7/B/RGB/e-0842_well-B2_p-216_cell-18_zoom-35_registered_line_2-1-1.tif (RGB).tif]

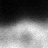

Supplement: Supplementary file 11 — Source data Fig. 7 [file 44318_2026_747_MOESM11_ESM.zip › Figure 7/B/RGB/e-0842_well-B2_p-216_cell-18_zoom-35_registered_line_2-1-1_ch02.tif]

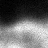

Supplement: Supplementary file 11 — Source data Fig. 7 [file 44318_2026_747_MOESM11_ESM.zip › Figure 7/B/RGB/e-0842_well-B2_p-216_cell-18_zoom-35_registered_line_2-1-1_ch01.tif]

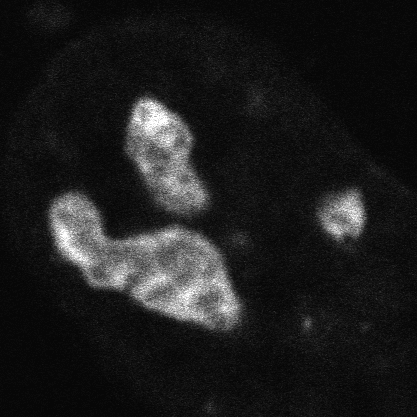

Supplement: Supplementary file 11 — Source data Fig. 7 [file 44318_2026_747_MOESM11_ESM.zip › Figure 7/B/RGB/e-0842_well-B2_p-216_cell-18_zoom-8-1_crop.tif]

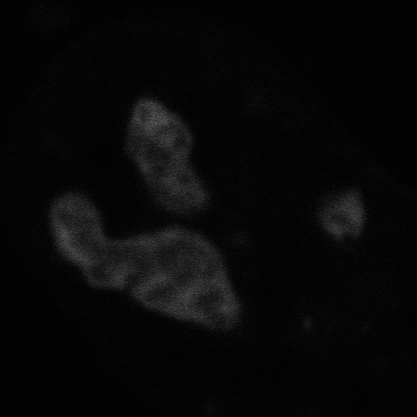

Supplement: Supplementary file 11 — Source data Fig. 7 [file 44318_2026_747_MOESM11_ESM.zip › Figure 7/B/raw/e-0842_well-B2_p-216_cell-18_zoom-8-1_slice5.tif]

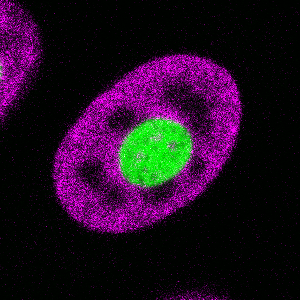

Supplement: Supplementary file 12 — Source data Fig. 8 [file 44318_2026_747_MOESM12_ESM.zip › Figure 8/D/RGB/e0962_exp08_AluI_DE_W0002_P0001_celle0962_exp08_AluI_DE_W0002_P0001_max_cell_01_hyperstack_resliced-1_f02.tif]

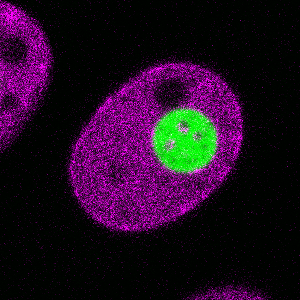

Supplement: Supplementary file 12 — Source data Fig. 8 [file 44318_2026_747_MOESM12_ESM.zip › Figure 8/D/RGB/e0962_exp08_AluI_DE_W0002_P0001_celle0962_exp08_AluI_DE_W0002_P0001_max_cell_01_hyperstack_resliced-1_f03.tif]

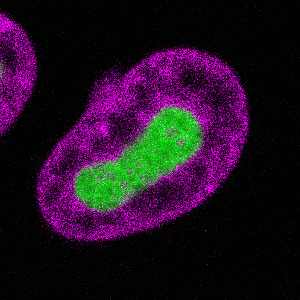

Supplement: Supplementary file 12 — Source data Fig. 8 [file 44318_2026_747_MOESM12_ESM.zip › Figure 8/D/RGB/e0962_exp08_AluI_DE_W0002_P0001_celle0962_exp08_AluI_DE_W0002_P0001_max_cell_01_hyperstack_resliced-1_f01.tif]

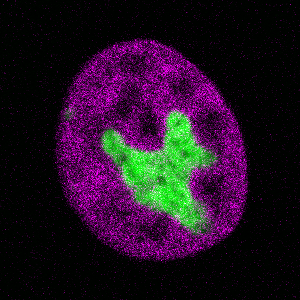

Supplement: Supplementary file 12 — Source data Fig. 8 [file 44318_2026_747_MOESM12_ESM.zip › Figure 8/D/RGB/e0962_exp08_control_DE_W0001_P0001_celle0962_exp08_control_DE_W0001_P0001_max_cell_02_hyperstack_resliced-1_f01.tif]

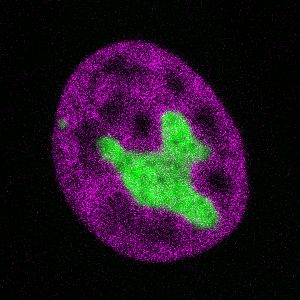

Supplement: Supplementary file 12 — Source data Fig. 8 [file 44318_2026_747_MOESM12_ESM.zip › Figure 8/D/RGB/e0962_exp08_control_DE_W0001_P0001_celle0962_exp08_control_DE_W0001_P0001_max_cell_02_hyperstack_resliced-1_f03.tif]

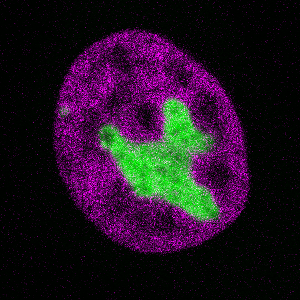

Supplement: Supplementary file 12 — Source data Fig. 8 [file 44318_2026_747_MOESM12_ESM.zip › Figure 8/D/RGB/e0962_exp08_control_DE_W0001_P0001_celle0962_exp08_control_DE_W0001_P0001_max_cell_02_hyperstack_resliced-1_f02.tif]

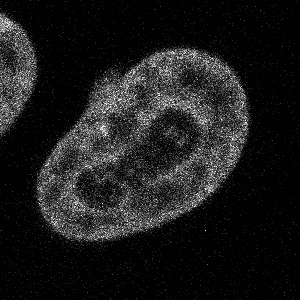

Supplement: Supplementary file 12 — Source data Fig. 8 [file 44318_2026_747_MOESM12_ESM.zip › Figure 8/D/raw/e0962_exp08_AluI.tif]

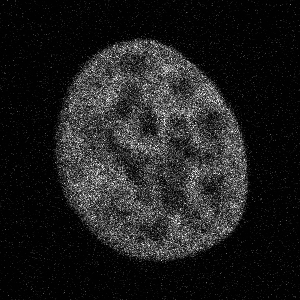

Supplement: Supplementary file 12 — Source data Fig. 8 [file 44318_2026_747_MOESM12_ESM.zip › Figure 8/D/raw/e0962_exp08_control.tif]

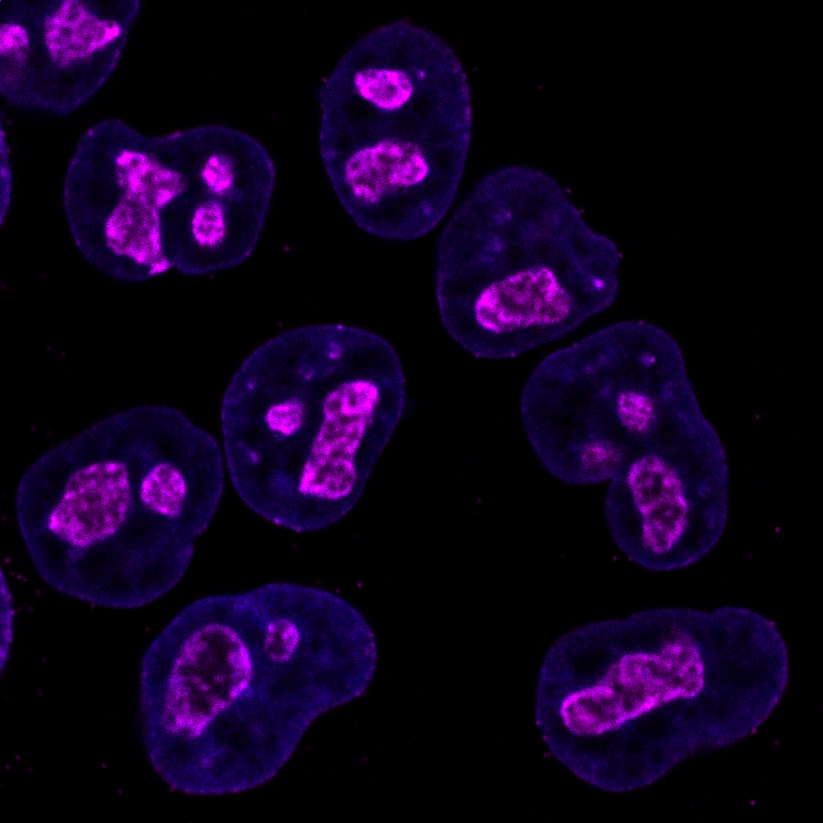

Supplement: Supplementary file 13 — Expanded View figures Source Data [file 44318_2026_747_MOESM13_ESM.zip › Figure_EV1/A/RGB/e1189_exp01_c77_zoom2_03_wider_cropping_DAPI_Ab.tif]

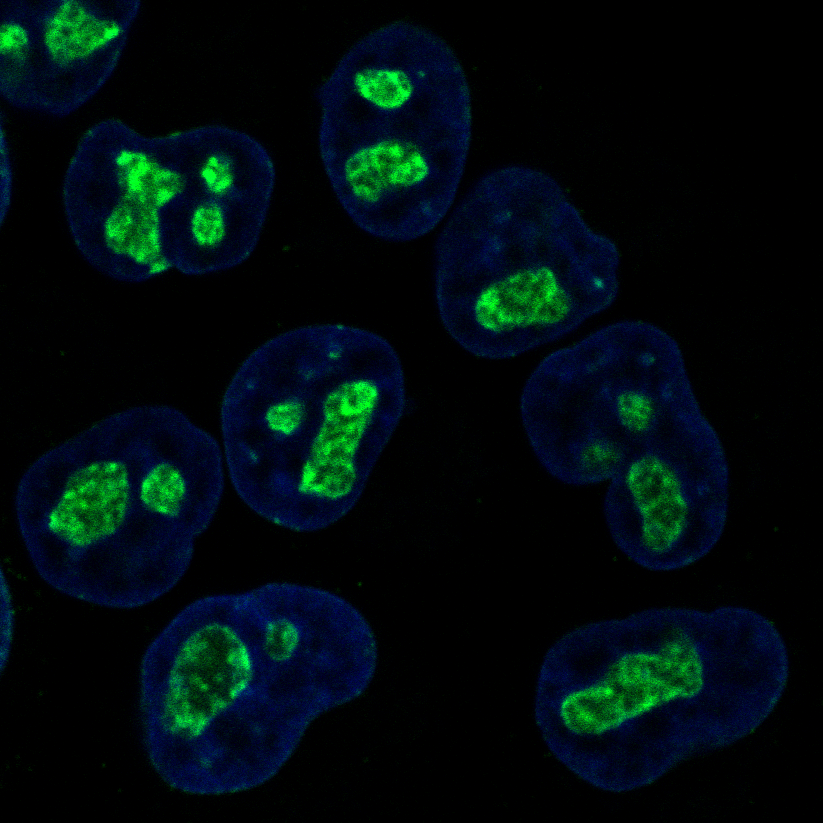

Supplement: Supplementary file 13 — Expanded View figures Source Data [file 44318_2026_747_MOESM13_ESM.zip › Figure_EV1/A/RGB/e1189_exp01_c77_zoom2_03_wider_cropping_DAPI_GFP.tif]

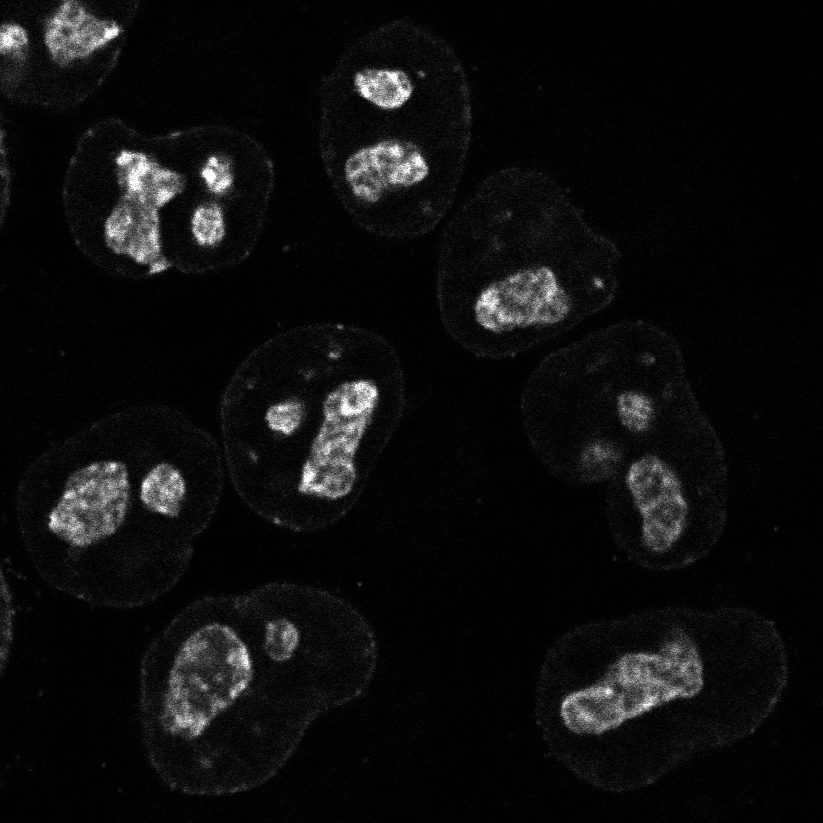

Supplement: Supplementary file 13 — Expanded View figures Source Data [file 44318_2026_747_MOESM13_ESM.zip › Figure_EV1/A/raw/e1189_exp01_c77_zoom2_03_wider_cropping.tif]

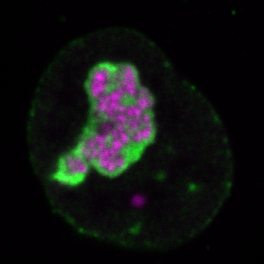

Supplement: Supplementary file 13 — Expanded View figures Source Data [file 44318_2026_747_MOESM13_ESM.zip › Figure_EV1/D/RGB/e1189_exp01_c318_zoom2_03_single_cell_Ki67_FBL.tif]

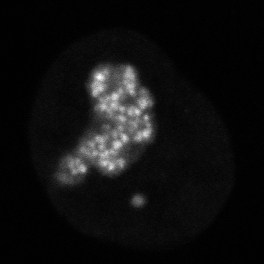

Supplement: Supplementary file 13 — Expanded View figures Source Data [file 44318_2026_747_MOESM13_ESM.zip › Figure_EV1/D/RGB/e1189_exp01_c318_zoom2_03_single_cell_ch04.tif]

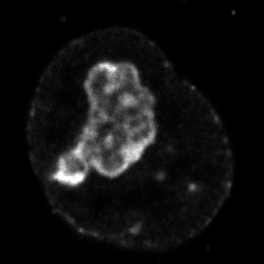

Supplement: Supplementary file 13 — Expanded View figures Source Data [file 44318_2026_747_MOESM13_ESM.zip › Figure_EV1/D/RGB/e1189_exp01_c318_zoom2_03_single_cell_ch01.tif]

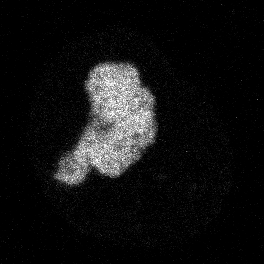

Supplement: Supplementary file 13 — Expanded View figures Source Data [file 44318_2026_747_MOESM13_ESM.zip › Figure_EV1/D/RGB/e1189_exp01_c318_zoom2_03_single_cell_ch02.tif]

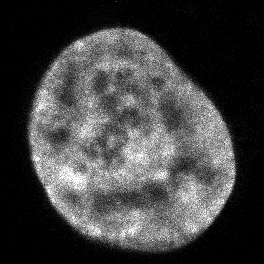

Supplement: Supplementary file 13 — Expanded View figures Source Data [file 44318_2026_747_MOESM13_ESM.zip › Figure_EV1/D/RGB/e1189_exp01_c318_zoom2_03_single_cell_ch03.tif]

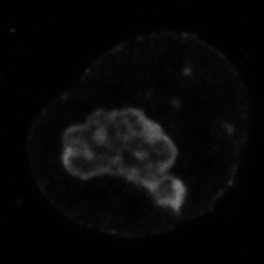

Supplement: Supplementary file 13 — Expanded View figures Source Data [file 44318_2026_747_MOESM13_ESM.zip › Figure_EV1/D/raw/e1189_exp01_c318_zoom2_03_single_cell.tif]

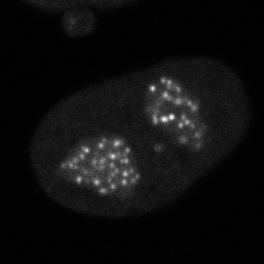

Supplement: Supplementary file 13 — Expanded View figures Source Data [file 44318_2026_747_MOESM13_ESM.zip › Figure_EV1/B/RGB/e1189_exp01_c567_zoom2_04_single_cell_crop_ch04.tif]

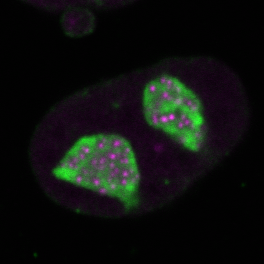

Supplement: Supplementary file 13 — Expanded View figures Source Data [file 44318_2026_747_MOESM13_ESM.zip › Figure_EV1/B/RGB/e1189_exp01_c567_zoom2_04_single_cell_crop_Ki67_UBF.tif]

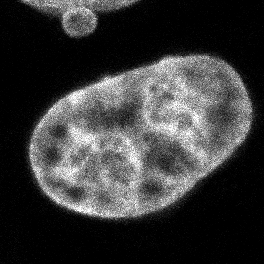

Supplement: Supplementary file 13 — Expanded View figures Source Data [file 44318_2026_747_MOESM13_ESM.zip › Figure_EV1/B/RGB/e1189_exp01_c567_zoom2_04_single_cell_crop_ch03.tif]

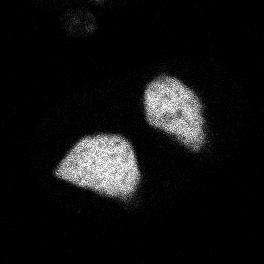

Supplement: Supplementary file 13 — Expanded View figures Source Data [file 44318_2026_747_MOESM13_ESM.zip › Figure_EV1/B/RGB/e1189_exp01_c567_zoom2_04_single_cell_crop_ch02.tif]

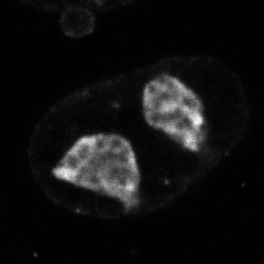

Supplement: Supplementary file 13 — Expanded View figures Source Data [file 44318_2026_747_MOESM13_ESM.zip › Figure_EV1/B/RGB/e1189_exp01_c567_zoom2_04_single_cell_crop_ch01.tif]

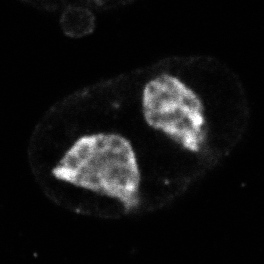

Supplement: Supplementary file 13 — Expanded View figures Source Data [file 44318_2026_747_MOESM13_ESM.zip › Figure_EV1/B/raw/e1189_exp01_c567_zoom2_04_single_cell_crop.tif]

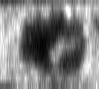

Supplement: Supplementary file 13 — Expanded View figures Source Data [file 44318_2026_747_MOESM13_ESM.zip › Figure_EV2/A/RGB/e-0550_well-A3_cell-1_cropped_zoom/e-0550_well-A3_cell-1_cropped_zoom_XZ_RGB.tif]

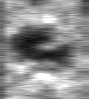

Supplement: Supplementary file 13 — Expanded View figures Source Data [file 44318_2026_747_MOESM13_ESM.zip › Figure_EV2/A/RGB/e-0550_well-A3_cell-1_cropped_zoom/e-0550_well-A3_cell-1_cropped_zoom_YZ_RGB.tif]

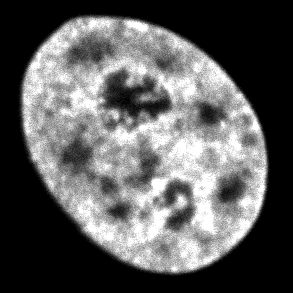

Supplement: Supplementary file 13 — Expanded View figures Source Data [file 44318_2026_747_MOESM13_ESM.zip › Figure_EV2/A/RGB/e-0550_well-A3_cell-1_cropped_zoom/e-0550_well-A3_cell-1_cropped_zoom_slice8_XY_RGB.tif]

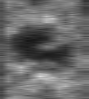

Supplement: Supplementary file 13 — Expanded View figures Source Data [file 44318_2026_747_MOESM13_ESM.zip › Figure_EV2/A/RGB/e-0550_well-A3_cell-1_cropped_zoom/e-0550_well-A3_cell-1_cropped_zoom_YZ.tif]

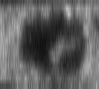

Supplement: Supplementary file 13 — Expanded View figures Source Data [file 44318_2026_747_MOESM13_ESM.zip › Figure_EV2/A/RGB/e-0550_well-A3_cell-1_cropped_zoom/e-0550_well-A3_cell-1_cropped_zoom_XZ.tif]

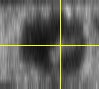

Supplement: Supplementary file 13 — Expanded View figures Source Data [file 44318_2026_747_MOESM13_ESM.zip › Figure_EV2/A/RGB/e-0550_well-A3_cell-1_cropped_zoom/XZ.jpg]

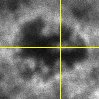

Supplement: Supplementary file 13 — Expanded View figures Source Data [file 44318_2026_747_MOESM13_ESM.zip › Figure_EV2/A/RGB/e-0550_well-A3_cell-1_cropped_zoom/XY.jpg]

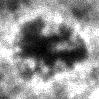

Supplement: Supplementary file 13 — Expanded View figures Source Data [file 44318_2026_747_MOESM13_ESM.zip › Figure_EV2/A/RGB/e-0550_well-A3_cell-1_cropped_zoom/e-0550_well-A3_cell-1_cropped_zoom_slice8_zoom_XY_RGB.tif]

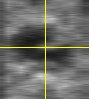

Supplement: Supplementary file 13 — Expanded View figures Source Data [file 44318_2026_747_MOESM13_ESM.zip › Figure_EV2/A/RGB/e-0550_well-A3_cell-1_cropped_zoom/YZ.jpg]

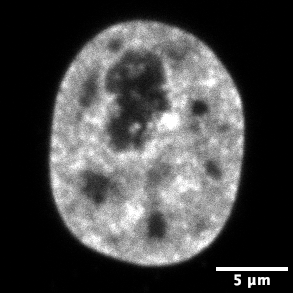

Supplement: Supplementary file 13 — Expanded View figures Source Data [file 44318_2026_747_MOESM13_ESM.zip › Figure_EV2/A/RGB/e-0550_well-A3_cell-7_cropped_zoom/e-0550_well-A3_cell-7_cropped_RGB_scale.tif]

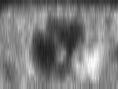

Supplement: Supplementary file 13 — Expanded View figures Source Data [file 44318_2026_747_MOESM13_ESM.zip › Figure_EV2/A/RGB/e-0550_well-A3_cell-7_cropped_zoom/e-0550_well-A3_cell-7_cropped_zoom_XZ.tif]

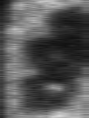

Supplement: Supplementary file 13 — Expanded View figures Source Data [file 44318_2026_747_MOESM13_ESM.zip › Figure_EV2/A/RGB/e-0550_well-A3_cell-7_cropped_zoom/e-0550_well-A3_cell-7_cropped_zoom_YZ.tif]

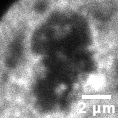

Supplement: Supplementary file 13 — Expanded View figures Source Data [file 44318_2026_747_MOESM13_ESM.zip › Figure_EV2/A/RGB/e-0550_well-A3_cell-7_cropped_zoom/e-0550_well-A3_cell-7_cropped_zoom_slice9_XY_RGB_scale.tif]

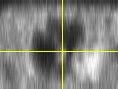

Supplement: Supplementary file 13 — Expanded View figures Source Data [file 44318_2026_747_MOESM13_ESM.zip › Figure_EV2/A/RGB/e-0550_well-A3_cell-7_cropped_zoom/XZ.jpg]

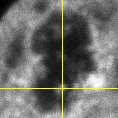

Supplement: Supplementary file 13 — Expanded View figures Source Data [file 44318_2026_747_MOESM13_ESM.zip › Figure_EV2/A/RGB/e-0550_well-A3_cell-7_cropped_zoom/XY.jpg]

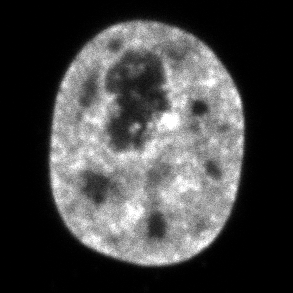

Supplement: Supplementary file 13 — Expanded View figures Source Data [file 44318_2026_747_MOESM13_ESM.zip › Figure_EV2/A/RGB/e-0550_well-A3_cell-7_cropped_zoom/e-0550_well-A3_cell-7_cropped_RGB.tif]

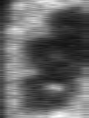

Supplement: Supplementary file 13 — Expanded View figures Source Data [file 44318_2026_747_MOESM13_ESM.zip › Figure_EV2/A/RGB/e-0550_well-A3_cell-7_cropped_zoom/e-0550_well-A3_cell-7_cropped_zoom_YZ_RGB.tif]

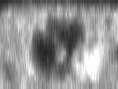

Supplement: Supplementary file 13 — Expanded View figures Source Data [file 44318_2026_747_MOESM13_ESM.zip › Figure_EV2/A/RGB/e-0550_well-A3_cell-7_cropped_zoom/e-0550_well-A3_cell-7_cropped_zoom_XZ_RGB.tif]

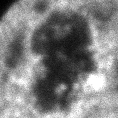

Supplement: Supplementary file 13 — Expanded View figures Source Data [file 44318_2026_747_MOESM13_ESM.zip › Figure_EV2/A/RGB/e-0550_well-A3_cell-7_cropped_zoom/e-0550_well-A3_cell-7_cropped_zoom_slice9_XY_RGB.tif]

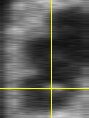

Supplement: Supplementary file 13 — Expanded View figures Source Data [file 44318_2026_747_MOESM13_ESM.zip › Figure_EV2/A/RGB/e-0550_well-A3_cell-7_cropped_zoom/YZ.jpg]

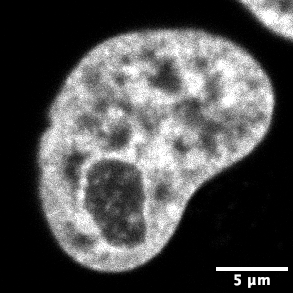

Supplement: Supplementary file 13 — Expanded View figures Source Data [file 44318_2026_747_MOESM13_ESM.zip › Figure_EV2/A/RGB/e-0550_well-A3_cell-5-and-cell-6_cropped_zoom/e-0550_well-A3_cell-5-and-cell-6_cropped_RGB_scale_5┬╡m.tif]

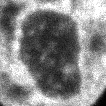

Supplement: Supplementary file 13 — Expanded View figures Source Data [file 44318_2026_747_MOESM13_ESM.zip › Figure_EV2/A/RGB/e-0550_well-A3_cell-5-and-cell-6_cropped_zoom/e-0550_well-A3_cell-5-and-cell-6_cropped_zoom_XY_RGB.tif]

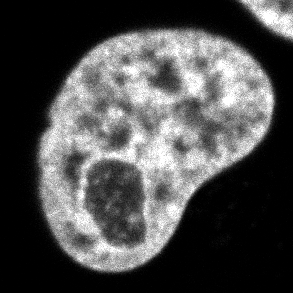

Supplement: Supplementary file 13 — Expanded View figures Source Data [file 44318_2026_747_MOESM13_ESM.zip › Figure_EV2/A/RGB/e-0550_well-A3_cell-5-and-cell-6_cropped_zoom/e-0550_well-A3_cell-5-and-cell-6_cropped_RGB.tif]

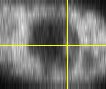

Supplement: Supplementary file 13 — Expanded View figures Source Data [file 44318_2026_747_MOESM13_ESM.zip › Figure_EV2/A/RGB/e-0550_well-A3_cell-5-and-cell-6_cropped_zoom/XZ.jpg]

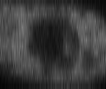

Supplement: Supplementary file 13 — Expanded View figures Source Data [file 44318_2026_747_MOESM13_ESM.zip › Figure_EV2/A/RGB/e-0550_well-A3_cell-5-and-cell-6_cropped_zoom/e-0550_well-A3_cell-5-and-cell-6_cropped_zoom_XZ.tif]

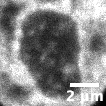

Supplement: Supplementary file 13 — Expanded View figures Source Data [file 44318_2026_747_MOESM13_ESM.zip › Figure_EV2/A/RGB/e-0550_well-A3_cell-5-and-cell-6_cropped_zoom/e-0550_well-A3_cell-5-and-cell-6_cropped_zoom_XY_RGB_scale.tif]

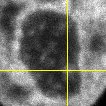

Supplement: Supplementary file 13 — Expanded View figures Source Data [file 44318_2026_747_MOESM13_ESM.zip › Figure_EV2/A/RGB/e-0550_well-A3_cell-5-and-cell-6_cropped_zoom/XY.jpg]

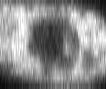

Supplement: Supplementary file 13 — Expanded View figures Source Data [file 44318_2026_747_MOESM13_ESM.zip › Figure_EV2/A/RGB/e-0550_well-A3_cell-5-and-cell-6_cropped_zoom/e-0550_well-A3_cell-5-and-cell-6_cropped_zoom_XZ_RGB.tif]

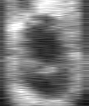

Supplement: Supplementary file 13 — Expanded View figures Source Data [file 44318_2026_747_MOESM13_ESM.zip › Figure_EV2/A/RGB/e-0550_well-A3_cell-5-and-cell-6_cropped_zoom/e-0550_well-A3_cell-5-and-cell-6_cropped_zoom_YZ_RGB.tif]

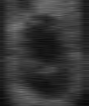

Supplement: Supplementary file 13 — Expanded View figures Source Data [file 44318_2026_747_MOESM13_ESM.zip › Figure_EV2/A/RGB/e-0550_well-A3_cell-5-and-cell-6_cropped_zoom/e-0550_well-A3_cell-5-and-cell-6_cropped_zoom_YZ.tif]

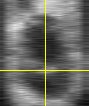

Supplement: Supplementary file 13 — Expanded View figures Source Data [file 44318_2026_747_MOESM13_ESM.zip › Figure_EV2/A/RGB/e-0550_well-A3_cell-5-and-cell-6_cropped_zoom/YZ.jpg]

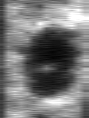

Supplement: Supplementary file 13 — Expanded View figures Source Data [file 44318_2026_747_MOESM13_ESM.zip › Figure_EV2/A/RGB/e-0550_well-A3_cell-12_round-nucleoli-with-retreated-H2B_cropped_zoom/e-0550_well-A3_cell-12_round-nucleoli-with-retreated-H2B_cropped_zoom_YZ_RGB.tif]

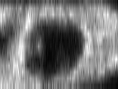

Supplement: Supplementary file 13 — Expanded View figures Source Data [file 44318_2026_747_MOESM13_ESM.zip › Figure_EV2/A/RGB/e-0550_well-A3_cell-12_round-nucleoli-with-retreated-H2B_cropped_zoom/e-0550_well-A3_cell-12_round-nucleoli-with-retreated-H2B_cropped_zoom_XZ_RGB.tif]

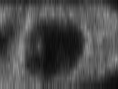

Supplement: Supplementary file 13 — Expanded View figures Source Data [file 44318_2026_747_MOESM13_ESM.zip › Figure_EV2/A/RGB/e-0550_well-A3_cell-12_round-nucleoli-with-retreated-H2B_cropped_zoom/e-0550_well-A3_cell-12_round-nucleoli-with-retreated-H2B_cropped_zoom_XZ.tif]

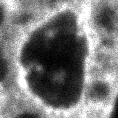

Supplement: Supplementary file 13 — Expanded View figures Source Data [file 44318_2026_747_MOESM13_ESM.zip › Figure_EV2/A/RGB/e-0550_well-A3_cell-12_round-nucleoli-with-retreated-H2B_cropped_zoom/e-0550_well-A3_cell-12_round-nucleoli-with-retreated-H2B_cropped_zoom_slice8_XY_RGB.tif]

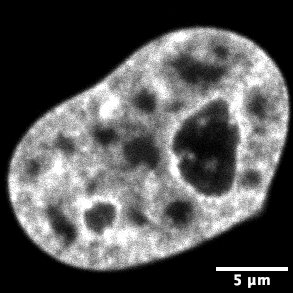

Supplement: Supplementary file 13 — Expanded View figures Source Data [file 44318_2026_747_MOESM13_ESM.zip › Figure_EV2/A/RGB/e-0550_well-A3_cell-12_round-nucleoli-with-retreated-H2B_cropped_zoom/e-0550_well-A3_cell-12_round-nucleoli-with-retreated-H2B_cropped_RGB_scale.tif]

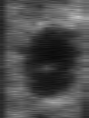

Supplement: Supplementary file 13 — Expanded View figures Source Data [file 44318_2026_747_MOESM13_ESM.zip › Figure_EV2/A/RGB/e-0550_well-A3_cell-12_round-nucleoli-with-retreated-H2B_cropped_zoom/e-0550_well-A3_cell-12_round-nucleoli-with-retreated-H2B_cropped_zoom_YZ.tif]
