# Supplementary material for: Ki-67 shapes the nucleolus by anchoring chromatin via its amphiphilic properties
Source: EMBO J. 2026 Mar 24;45(9):3156–91. doi: 10.1038/s44318-026-00747-7 (PMC13144362; doi:10.1038/s44318-026-00747-7)
Supplement: Supplementary file 13 — Expanded View figures Source Data [file 44318_2026_747_MOESM13_ESM.zip › Figure_EV7/A/Full_FACS_results.pdf]

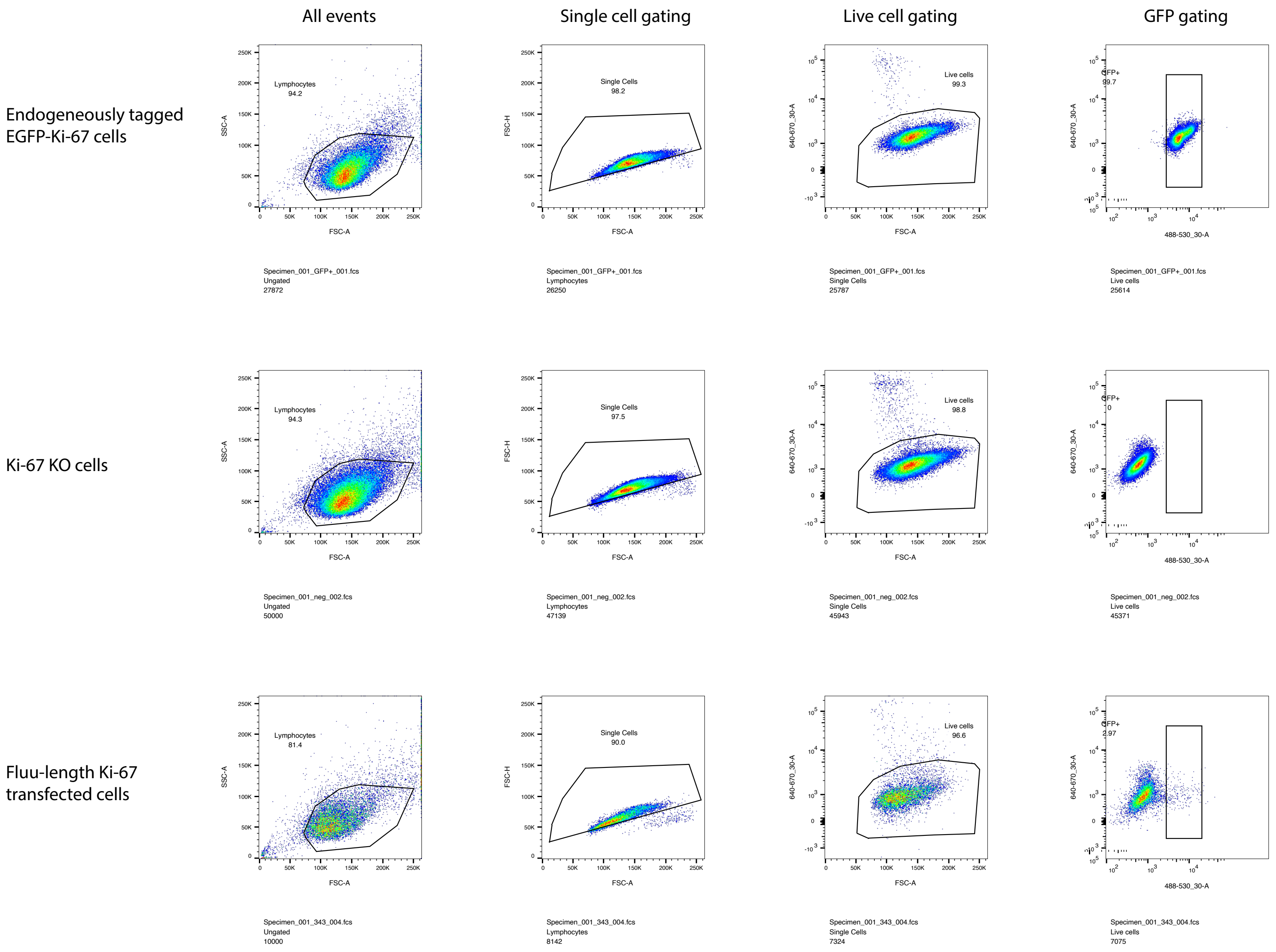
All events
Single cell gating
Live cell gating
GFP gating
Endogeneously tagged EGFP-Ki-67 cells
Ki-67 KO cells
Fluu-length Ki-67 transfected cells
Lymphocytes 94.2
Single Cells 98.2
Live cells 99.3
GFP+ 99.7
Specimen_001_GFP+_001.fcs
Ungated
27872
Lymphocytes
26250
Single Cells
25787
Live cells
25614
Lymphocytes 94.3
Single Cells 97.5
Live cells 98.8
GFP+ 0
Specimen_001_neg_002.fcs
Ungated
50000
Lymphocytes
47139
Single Cells
45943
Live cells
45371
Lymphocytes 81.4
Single Cells 90.0
Live cells 96.6
GFP+ 2.97
Specimen_001_343_004.fcs
Ungated
10000
Lymphocytes
8142
Single Cells
7324
Live cells
7075
FSC-A
SSC-A
FSC-H
640-670_30-A
488-530_30-A
