# Supplementary figures and images for: Ki-67 shapes the nucleolus by anchoring chromatin via its amphiphilic properties (part 5 of 5)
Source: EMBO J. 2026 Mar 24;45(9):3156–91. doi: 10.1038/s44318-026-00747-7 (PMC13144362; doi:10.1038/s44318-026-00747-7)

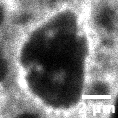

Supplement: Supplementary file 13 — Expanded View figures Source Data [file 44318_2026_747_MOESM13_ESM.zip › Figure_EV2/A/RGB/e-0550_well-A3_cell-12_round-nucleoli-with-retreated-H2B_cropped_zoom/e-0550_well-A3_cell-12_round-nucleoli-with-retreated-H2B_cropped_zoom_slice8_XY_RGB_scale.tif]

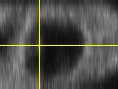

Supplement: Supplementary file 13 — Expanded View figures Source Data [file 44318_2026_747_MOESM13_ESM.zip › Figure_EV2/A/RGB/e-0550_well-A3_cell-12_round-nucleoli-with-retreated-H2B_cropped_zoom/XZ.jpg]

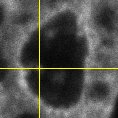

Supplement: Supplementary file 13 — Expanded View figures Source Data [file 44318_2026_747_MOESM13_ESM.zip › Figure_EV2/A/RGB/e-0550_well-A3_cell-12_round-nucleoli-with-retreated-H2B_cropped_zoom/XY.jpg]

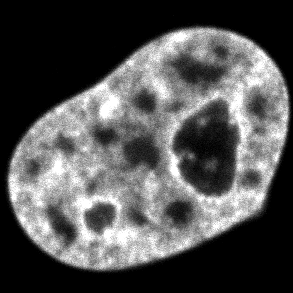

Supplement: Supplementary file 13 — Expanded View figures Source Data [file 44318_2026_747_MOESM13_ESM.zip › Figure_EV2/A/RGB/e-0550_well-A3_cell-12_round-nucleoli-with-retreated-H2B_cropped_zoom/e-0550_well-A3_cell-12_round-nucleoli-with-retreated-H2B_cropped_RGB.tif]

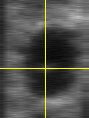

Supplement: Supplementary file 13 — Expanded View figures Source Data [file 44318_2026_747_MOESM13_ESM.zip › Figure_EV2/A/RGB/e-0550_well-A3_cell-12_round-nucleoli-with-retreated-H2B_cropped_zoom/YZ.jpg]

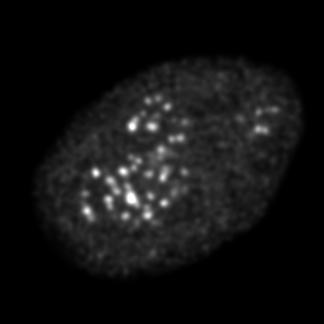

Supplement: Supplementary file 13 — Expanded View figures Source Data [file 44318_2026_747_MOESM13_ESM.zip › Figure_EV3/A/RGB/e1290_exp02_0min02_cell01_decon_slice7_ch02.tif]

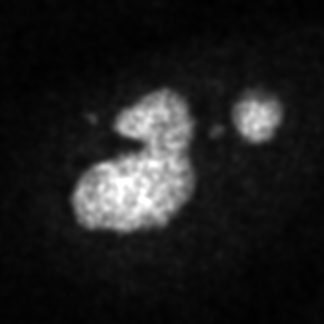

Supplement: Supplementary file 13 — Expanded View figures Source Data [file 44318_2026_747_MOESM13_ESM.zip › Figure_EV3/A/RGB/e1290_exp02_0min02_cell01_decon_slice7_ch03.tif]

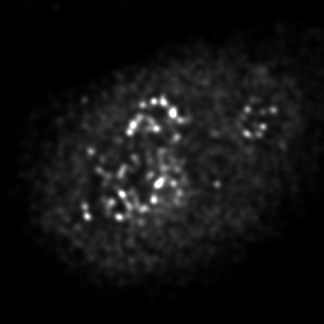

Supplement: Supplementary file 13 — Expanded View figures Source Data [file 44318_2026_747_MOESM13_ESM.zip › Figure_EV3/A/RGB/e1290_exp02_0min02_cell01_decon_slice7_ch01.tif]

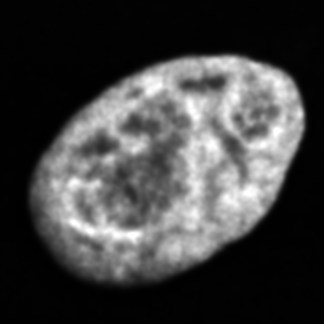

Supplement: Supplementary file 13 — Expanded View figures Source Data [file 44318_2026_747_MOESM13_ESM.zip › Figure_EV3/A/RGB/e1290_exp02_0min02_cell01_decon_slice7_ch04.tif]

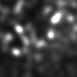

Supplement: Supplementary file 13 — Expanded View figures Source Data [file 44318_2026_747_MOESM13_ESM.zip › Figure_EV3/A/RGB/e1290_exp02_0min02_cell01_decon_slice7_ch01-1.tif]

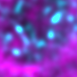

Supplement: Supplementary file 13 — Expanded View figures Source Data [file 44318_2026_747_MOESM13_ESM.zip › Figure_EV3/A/RGB/e1290_exp02_0min02_cell01_decon_slice7_rDNA_DNA-1.tif]

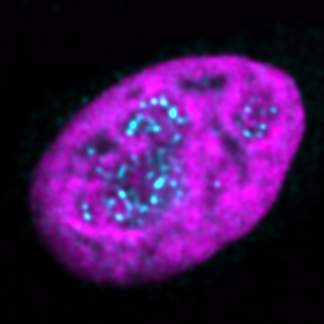

Supplement: Supplementary file 13 — Expanded View figures Source Data [file 44318_2026_747_MOESM13_ESM.zip › Figure_EV3/A/RGB/e1290_exp02_0min02_cell01_decon_slice7_rDNA_DNA.tif]

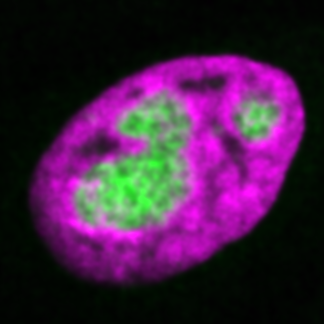

Supplement: Supplementary file 13 — Expanded View figures Source Data [file 44318_2026_747_MOESM13_ESM.zip › Figure_EV3/A/RGB/e1290_exp02_0min02_cell01_decon_slice7_NPM1_DNA_scale.tif]

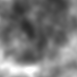

Supplement: Supplementary file 13 — Expanded View figures Source Data [file 44318_2026_747_MOESM13_ESM.zip › Figure_EV3/A/RGB/e1290_exp02_0min02_cell01_decon_slice7_ch04-1.tif]

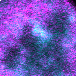

Supplement: Supplementary file 13 — Expanded View figures Source Data [file 44318_2026_747_MOESM13_ESM.zip › Figure_EV3/C/RGB/e1587_exp02_c391_ab48-HP1b_02-2_slice4_zoom.tif (RGB).tif]

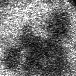

Supplement: Supplementary file 13 — Expanded View figures Source Data [file 44318_2026_747_MOESM13_ESM.zip › Figure_EV3/C/RGB/e1587_exp02_c391_ab48-HP1b_02-2_slice4_zoom_ch04.tif]

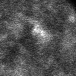

Supplement: Supplementary file 13 — Expanded View figures Source Data [file 44318_2026_747_MOESM13_ESM.zip › Figure_EV3/C/RGB/e1587_exp02_c391_ab48-HP1b_02-2_slice4_zoom_ch03.tif]

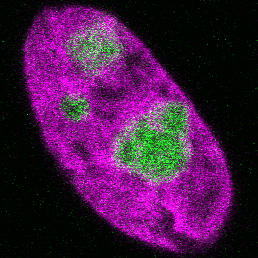

Supplement: Supplementary file 13 — Expanded View figures Source Data [file 44318_2026_747_MOESM13_ESM.zip › Figure_EV3/C/RGB/e1587_exp02_c391_ab48-HP1b_02-2_slice4_NPM1_DNA (RGB).tif]

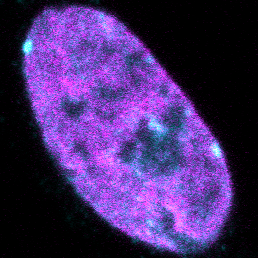

Supplement: Supplementary file 13 — Expanded View figures Source Data [file 44318_2026_747_MOESM13_ESM.zip › Figure_EV3/C/RGB/e1587_exp02_c391_ab48-HP1b_02-2_slice4.tif (RGB).tif]

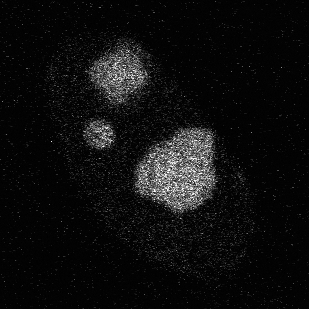

Supplement: Supplementary file 13 — Expanded View figures Source Data [file 44318_2026_747_MOESM13_ESM.zip › Figure_EV3/C/raw/e1587_exp02_c391_ab48-HP1b_02-2_slice4.tif]

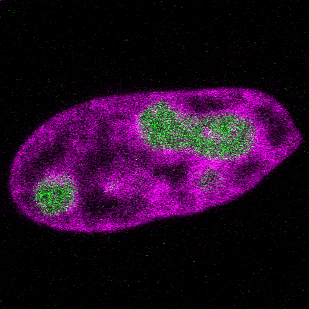

Supplement: Supplementary file 13 — Expanded View figures Source Data [file 44318_2026_747_MOESM13_ESM.zip › Figure_EV3/D/RGB/e1587_exp02_c391_ab45-H3K9me3_01-1_sclice5_NPM1_DNA (RGB).tif]

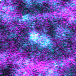

Supplement: Supplementary file 13 — Expanded View figures Source Data [file 44318_2026_747_MOESM13_ESM.zip › Figure_EV3/D/RGB/e1587_exp02_c391_ab45-H3K9me3_01-1_sclice5_zoom.tif (RGB).tif]

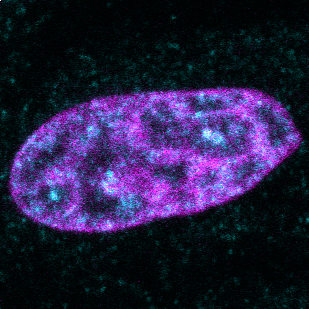

Supplement: Supplementary file 13 — Expanded View figures Source Data [file 44318_2026_747_MOESM13_ESM.zip › Figure_EV3/D/RGB/e1587_exp02_c391_ab45-H3K9me3_01-1_sclice5.tif (RGB).tif]

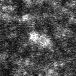

Supplement: Supplementary file 13 — Expanded View figures Source Data [file 44318_2026_747_MOESM13_ESM.zip › Figure_EV3/D/RGB/e1587_exp02_c391_ab45-H3K9me3_01-1_sclice5_zoom_ch03.tif]

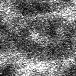

Supplement: Supplementary file 13 — Expanded View figures Source Data [file 44318_2026_747_MOESM13_ESM.zip › Figure_EV3/D/RGB/e1587_exp02_c391_ab45-H3K9me3_01-1_sclice5_zoom_ch04.tif]

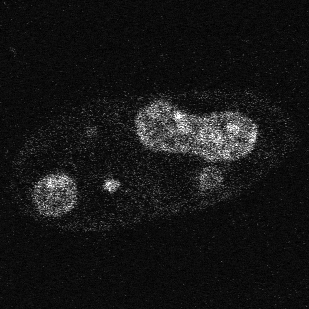

Supplement: Supplementary file 13 — Expanded View figures Source Data [file 44318_2026_747_MOESM13_ESM.zip › Figure_EV3/D/raw/e1587_exp02_c391_ab45-H3K9me3_01-1_sclice5.tif]

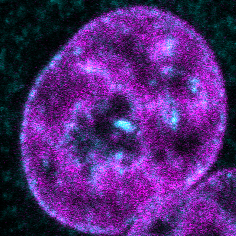

Supplement: Supplementary file 13 — Expanded View figures Source Data [file 44318_2026_747_MOESM13_ESM.zip › Figure_EV3/E/RGB/e1587_exp02_c391_ab47-H4K20me3_04-1_slice3.tif (RGB).tif]

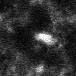

Supplement: Supplementary file 13 — Expanded View figures Source Data [file 44318_2026_747_MOESM13_ESM.zip › Figure_EV3/E/RGB/e1587_exp02_c391_ab47-H4K20me3_04-1_slice3_zooom_ch03.tif]

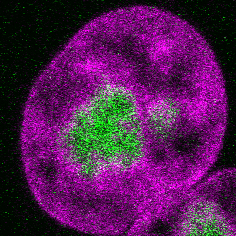

Supplement: Supplementary file 13 — Expanded View figures Source Data [file 44318_2026_747_MOESM13_ESM.zip › Figure_EV3/E/RGB/e1587_exp02_c391_ab47-H4K20me3_04-1_slice3_NPM1_DNA(RGB).tif]

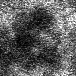

Supplement: Supplementary file 13 — Expanded View figures Source Data [file 44318_2026_747_MOESM13_ESM.zip › Figure_EV3/E/RGB/e1587_exp02_c391_ab47-H4K20me3_04-1_slice3_zooom_ch04.tif]

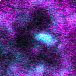

Supplement: Supplementary file 13 — Expanded View figures Source Data [file 44318_2026_747_MOESM13_ESM.zip › Figure_EV3/E/RGB/e1587_exp02_c391_ab47-H4K20me3_04-1_slice3_zooom.tif (RGB).tif]

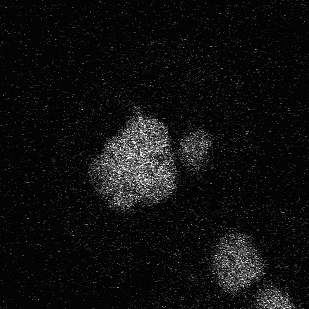

Supplement: Supplementary file 13 — Expanded View figures Source Data [file 44318_2026_747_MOESM13_ESM.zip › Figure_EV3/E/raw/e1587_exp02_c391_ab47-H4K20me3_04-1_slice3.tif]

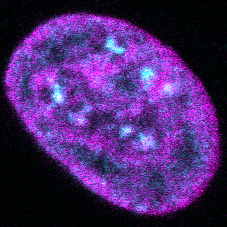

Supplement: Supplementary file 13 — Expanded View figures Source Data [file 44318_2026_747_MOESM13_ESM.zip › Figure_EV3/B/RGB/e1587_exp02_c391_ab46-HP1a_05-1_slice3.tif (RGB).tif]

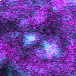

Supplement: Supplementary file 13 — Expanded View figures Source Data [file 44318_2026_747_MOESM13_ESM.zip › Figure_EV3/B/RGB/e1587_exp02_c391_ab46-HP1a_05-1_slice3_zoom.tif (RGB).tif]

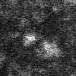

Supplement: Supplementary file 13 — Expanded View figures Source Data [file 44318_2026_747_MOESM13_ESM.zip › Figure_EV3/B/RGB/e1587_exp02_c391_ab46-HP1a_05-1_slice3_zoom_ch03.tif]

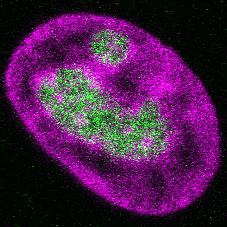

Supplement: Supplementary file 13 — Expanded View figures Source Data [file 44318_2026_747_MOESM13_ESM.zip › Figure_EV3/B/RGB/e1587_exp02_c391_ab46-HP1a_05-1_slice3_NPM1_DNA(RGB).tif]

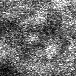

Supplement: Supplementary file 13 — Expanded View figures Source Data [file 44318_2026_747_MOESM13_ESM.zip › Figure_EV3/B/RGB/e1587_exp02_c391_ab46-HP1a_05-1_slice3_zoom_ch04.tif]

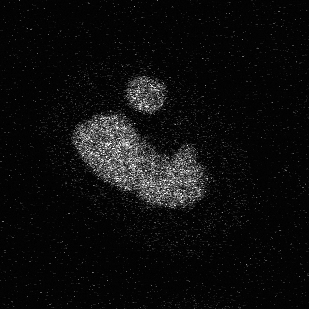

Supplement: Supplementary file 13 — Expanded View figures Source Data [file 44318_2026_747_MOESM13_ESM.zip › Figure_EV3/B/raw/e1587_exp02_c391_ab46-HP1a_05-1_slice3.tif]

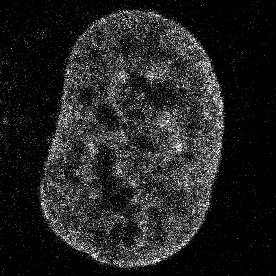

Supplement: Supplementary file 13 — Expanded View figures Source Data [file 44318_2026_747_MOESM13_ESM.zip › Figure_EV4/A/RGB/e1576_exp03_c309_siControl_DE_3_W0009_P0001_T0001-1_ch01.tif]

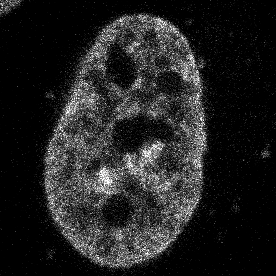

Supplement: Supplementary file 13 — Expanded View figures Source Data [file 44318_2026_747_MOESM13_ESM.zip › Figure_EV4/A/RGB/e1576_exp03_c309_siKi67_1__DE_3_W0005_P0001_T0001-1_ch01.tif]

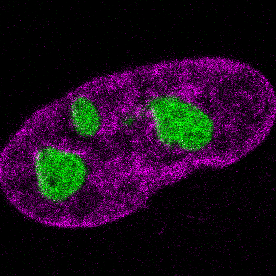

Supplement: Supplementary file 13 — Expanded View figures Source Data [file 44318_2026_747_MOESM13_ESM.zip › Figure_EV4/A/RGB/e1576_exp03_c309_siKi67_2__DE_3_W0003_P0001_T0001-1.tif (RGB).tif]

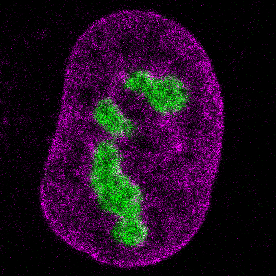

Supplement: Supplementary file 13 — Expanded View figures Source Data [file 44318_2026_747_MOESM13_ESM.zip › Figure_EV4/A/RGB/e1576_exp03_c309_siControl_DE_3_W0009_P0001_T0001-1.tif (RGB).tif]

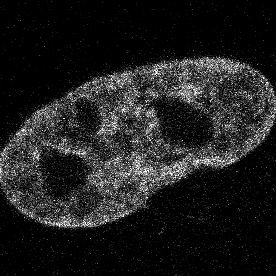

Supplement: Supplementary file 13 — Expanded View figures Source Data [file 44318_2026_747_MOESM13_ESM.zip › Figure_EV4/A/RGB/e1576_exp03_c309_siKi67_2__DE_3_W0003_P0001_T0001-1_ch01.tif]

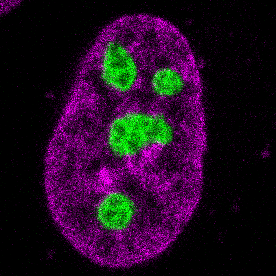

Supplement: Supplementary file 13 — Expanded View figures Source Data [file 44318_2026_747_MOESM13_ESM.zip › Figure_EV4/A/RGB/e1576_exp03_c309_siKi67_1__DE_3_W0005_P0001_T0001-1.tif (RGB).tif]

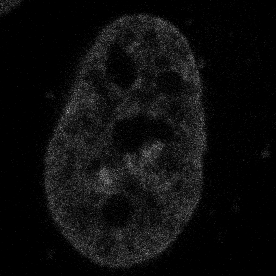

Supplement: Supplementary file 13 — Expanded View figures Source Data [file 44318_2026_747_MOESM13_ESM.zip › Figure_EV4/A/raw/e1576_exp03_c309_siKi67_1__DE_3_W0005_P0001_T0001-1.tif]

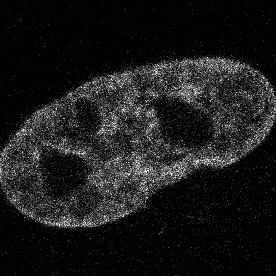

Supplement: Supplementary file 13 — Expanded View figures Source Data [file 44318_2026_747_MOESM13_ESM.zip › Figure_EV4/A/raw/e1576_exp03_c309_siKi67_2__DE_3_W0003_P0001_T0001-1.tif]

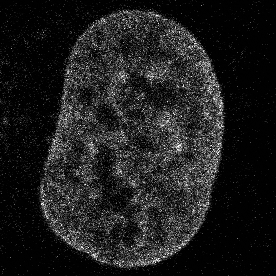

Supplement: Supplementary file 13 — Expanded View figures Source Data [file 44318_2026_747_MOESM13_ESM.zip › Figure_EV4/A/raw/e1576_exp03_c309_siControl_DE_3_W0009_P0001_T0001-1.tif]

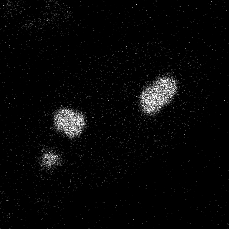

Supplement: Supplementary file 13 — Expanded View figures Source Data [file 44318_2026_747_MOESM13_ESM.zip › Figure_EV4/E/RGB/e1576_exp02_c322_siKi67_1_DE_3_W0007_P0001_T0001-3_ch01.tif]

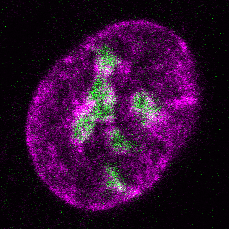

Supplement: Supplementary file 13 — Expanded View figures Source Data [file 44318_2026_747_MOESM13_ESM.zip › Figure_EV4/E/RGB/e1576_exp02_c322_siControl_DE_3_W0007_P0001_T0001-1.tif (RGB).tif]

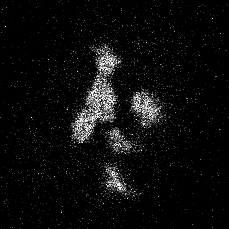

Supplement: Supplementary file 13 — Expanded View figures Source Data [file 44318_2026_747_MOESM13_ESM.zip › Figure_EV4/E/RGB/e1576_exp02_c322_siControl_DE_3_W0007_P0001_T0001-1_ch01.tif]

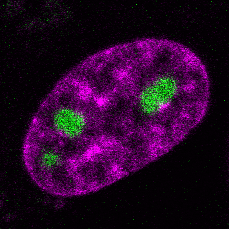

Supplement: Supplementary file 13 — Expanded View figures Source Data [file 44318_2026_747_MOESM13_ESM.zip › Figure_EV4/E/RGB/e1576_exp02_c322_siKi67_1_DE_3_W0007_P0001_T0001-3.tif (RGB).tif]

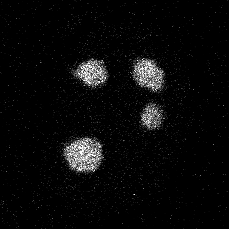

Supplement: Supplementary file 13 — Expanded View figures Source Data [file 44318_2026_747_MOESM13_ESM.zip › Figure_EV4/E/RGB/e1576_exp02_c322_siKi67_2_DE_3_W0004_P0001_T0001-1_ch01.tif]

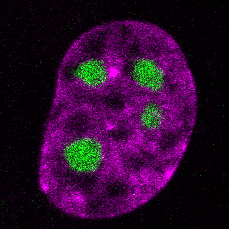

Supplement: Supplementary file 13 — Expanded View figures Source Data [file 44318_2026_747_MOESM13_ESM.zip › Figure_EV4/E/RGB/e1576_exp02_c322_siKi67_2_DE_3_W0004_P0001_T0001-1.tif (RGB).tif]

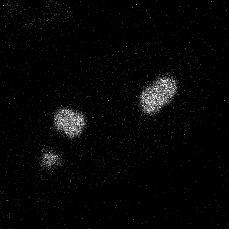

Supplement: Supplementary file 13 — Expanded View figures Source Data [file 44318_2026_747_MOESM13_ESM.zip › Figure_EV4/E/raw/e1576_exp02_c322_siKi67_1_DE_3_W0007_P0001_T0001-3.tif]

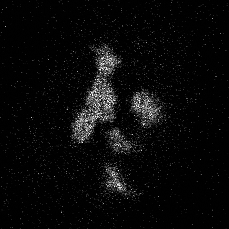

Supplement: Supplementary file 13 — Expanded View figures Source Data [file 44318_2026_747_MOESM13_ESM.zip › Figure_EV4/E/raw/e1576_exp02_c322_siControl_DE_3_W0007_P0001_T0001-1.tif]

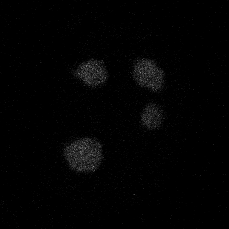

Supplement: Supplementary file 13 — Expanded View figures Source Data [file 44318_2026_747_MOESM13_ESM.zip › Figure_EV4/E/raw/e1576_exp02_c322_siKi67_2_DE_3_W0004_P0001_T0001-1.tif]

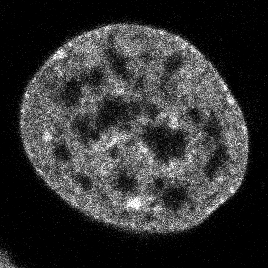

Supplement: Supplementary file 13 — Expanded View figures Source Data [file 44318_2026_747_MOESM13_ESM.zip › Figure_EV5/A/RGB/e0986_exp06_c567_XWNeg9_01_add_probability_map-1_ch02.tif]

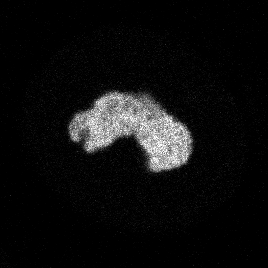

Supplement: Supplementary file 13 — Expanded View figures Source Data [file 44318_2026_747_MOESM13_ESM.zip › Figure_EV5/A/RGB/e0986_exp06_c567_XWNeg9_01_add_probability_map-1_ch03.tif]

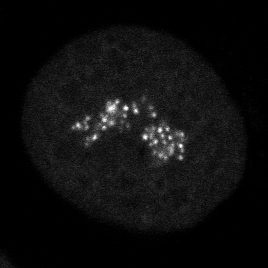

Supplement: Supplementary file 13 — Expanded View figures Source Data [file 44318_2026_747_MOESM13_ESM.zip › Figure_EV5/A/RGB/e0986_exp06_c567_XWNeg9_01_add_probability_map-1_ch01.tif]

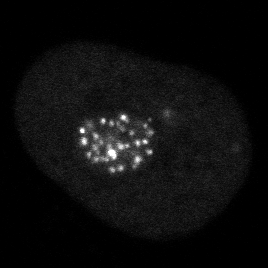

Supplement: Supplementary file 13 — Expanded View figures Source Data [file 44318_2026_747_MOESM13_ESM.zip › Figure_EV5/A/RGB/e0986_exp06_c567_siKi67_2_04_add_probability_map-1_ch01.tif]

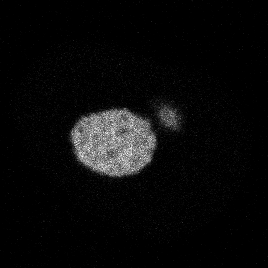

Supplement: Supplementary file 13 — Expanded View figures Source Data [file 44318_2026_747_MOESM13_ESM.zip › Figure_EV5/A/RGB/e0986_exp06_c567_siKi67_2_04_add_probability_map-1_ch03.tif]

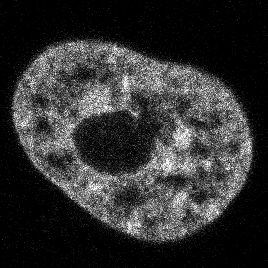

Supplement: Supplementary file 13 — Expanded View figures Source Data [file 44318_2026_747_MOESM13_ESM.zip › Figure_EV5/A/RGB/e0986_exp06_c567_siKi67_2_04_add_probability_map-1_ch02.tif]

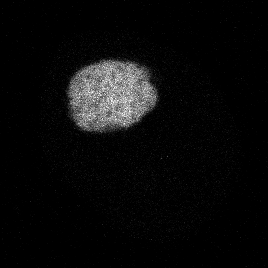

Supplement: Supplementary file 13 — Expanded View figures Source Data [file 44318_2026_747_MOESM13_ESM.zip › Figure_EV5/A/RGB/e0986_exp06_c567_siKi67_1_02_add_probability_map-1_ch03.tif]

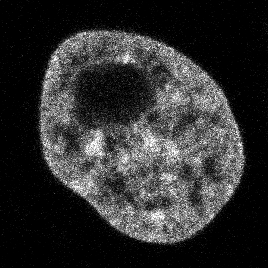

Supplement: Supplementary file 13 — Expanded View figures Source Data [file 44318_2026_747_MOESM13_ESM.zip › Figure_EV5/A/RGB/e0986_exp06_c567_siKi67_1_02_add_probability_map-1_ch02.tif]

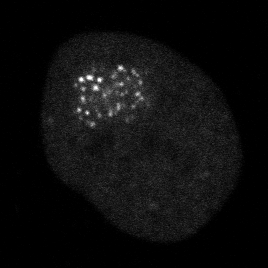

Supplement: Supplementary file 13 — Expanded View figures Source Data [file 44318_2026_747_MOESM13_ESM.zip › Figure_EV5/A/RGB/e0986_exp06_c567_siKi67_1_02_add_probability_map-1_ch01.tif]

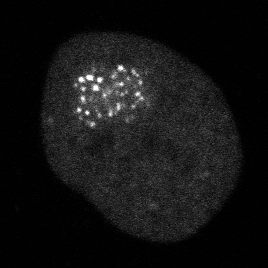

Supplement: Supplementary file 13 — Expanded View figures Source Data [file 44318_2026_747_MOESM13_ESM.zip › Figure_EV5/A/raw/e0986_exp06_c567_siKi67_1_02_add_probability_map-1.tif]

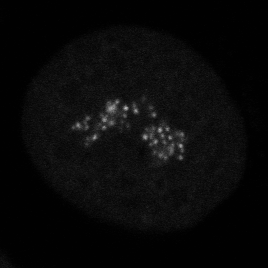

Supplement: Supplementary file 13 — Expanded View figures Source Data [file 44318_2026_747_MOESM13_ESM.zip › Figure_EV5/A/raw/e0986_exp06_c567_siControl_01_add_probability_map-1.tif]

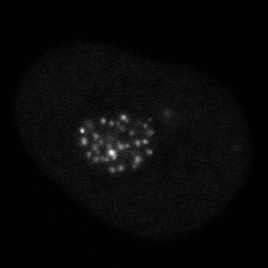

Supplement: Supplementary file 13 — Expanded View figures Source Data [file 44318_2026_747_MOESM13_ESM.zip › Figure_EV5/A/raw/e0986_exp06_c567_siKi67_2_04_add_probability_map-1.tif]

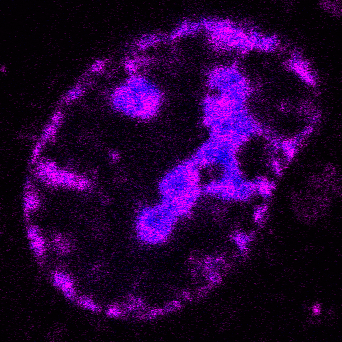

Supplement: Supplementary file 13 — Expanded View figures Source Data [file 44318_2026_747_MOESM13_ESM.zip › Figure_EV6/A/RGB/e1584_exp04_c309_p391_DE_3_W0003_P0001_T0001-1_NPM1_DNA.tif]

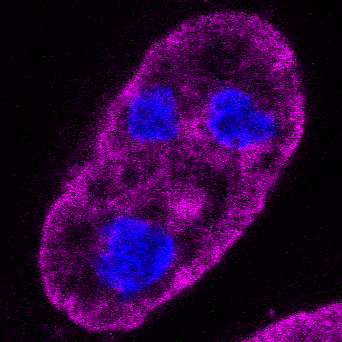

Supplement: Supplementary file 13 — Expanded View figures Source Data [file 44318_2026_747_MOESM13_ESM.zip › Figure_EV6/A/RGB/e1584_exp04_c309_mock_DE_3_W0013_P0001_T0001-1_NPM1_DNA.tif.tif]

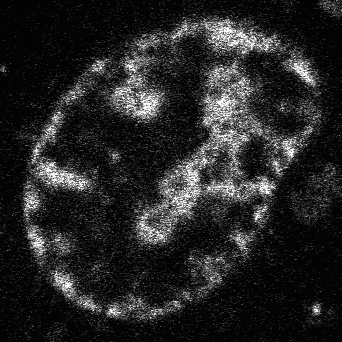

Supplement: Supplementary file 13 — Expanded View figures Source Data [file 44318_2026_747_MOESM13_ESM.zip › Figure_EV6/A/RGB/e1584_exp04_c309_p391_DE_3_W0003_P0001_T0001-1_ch01.tif]

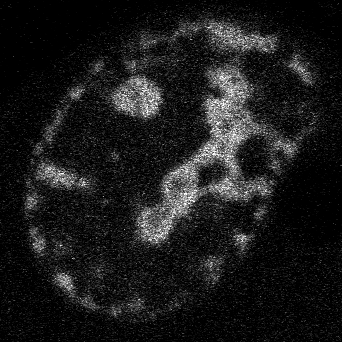

Supplement: Supplementary file 13 — Expanded View figures Source Data [file 44318_2026_747_MOESM13_ESM.zip › Figure_EV6/A/RGB/e1584_exp04_c309_p391_DE_3_W0003_P0001_T0001-1_ch03.tif]

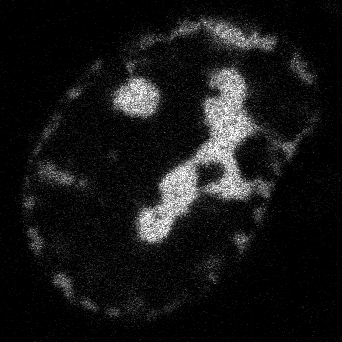

Supplement: Supplementary file 13 — Expanded View figures Source Data [file 44318_2026_747_MOESM13_ESM.zip › Figure_EV6/A/RGB/e1584_exp04_c309_p391_DE_3_W0003_P0001_T0001-1_ch02.tif]

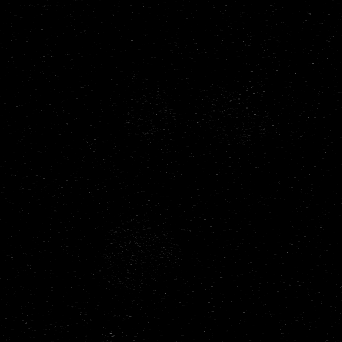

Supplement: Supplementary file 13 — Expanded View figures Source Data [file 44318_2026_747_MOESM13_ESM.zip › Figure_EV6/A/RGB/e1584_exp04_c309_mock_DE_3_W0013_P0001_T0001-1_ch03.tif]

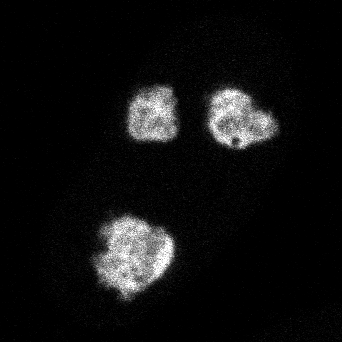

Supplement: Supplementary file 13 — Expanded View figures Source Data [file 44318_2026_747_MOESM13_ESM.zip › Figure_EV6/A/RGB/e1584_exp04_c309_mock_DE_3_W0013_P0001_T0001-1_ch02.tif]

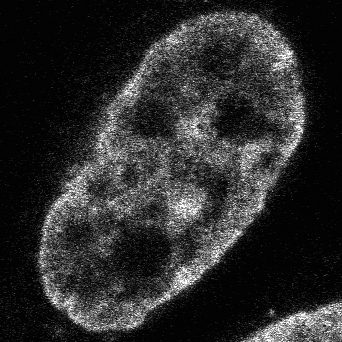

Supplement: Supplementary file 13 — Expanded View figures Source Data [file 44318_2026_747_MOESM13_ESM.zip › Figure_EV6/A/RGB/e1584_exp04_c309_mock_DE_3_W0013_P0001_T0001-1_ch01.tif]

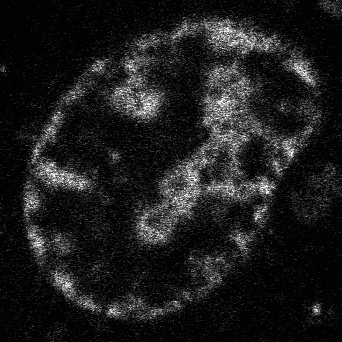

Supplement: Supplementary file 13 — Expanded View figures Source Data [file 44318_2026_747_MOESM13_ESM.zip › Figure_EV6/A/raw/e1584_exp04_c309_p391_DE_3_W0003_P0001_T0001-1.tif]

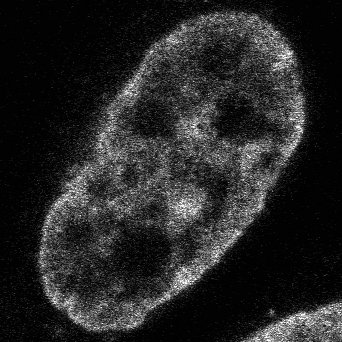

Supplement: Supplementary file 13 — Expanded View figures Source Data [file 44318_2026_747_MOESM13_ESM.zip › Figure_EV6/A/raw/e1584_exp04_c309_mock_DE_3_W0013_P0001_T0001-1.tif]

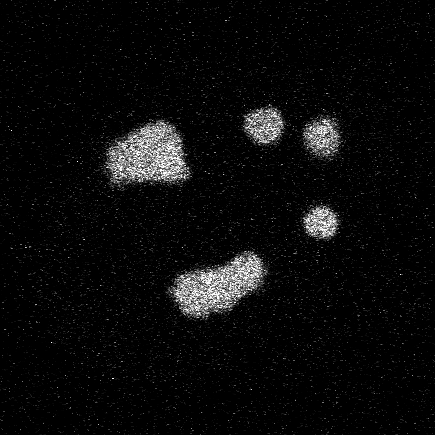

Supplement: Supplementary file 13 — Expanded View figures Source Data [file 44318_2026_747_MOESM13_ESM.zip › Figure_EV6/E/RGB/e1584_exp06_c322_mock_DE_3_W0002_P0001_T0001-1_ch03.tif]

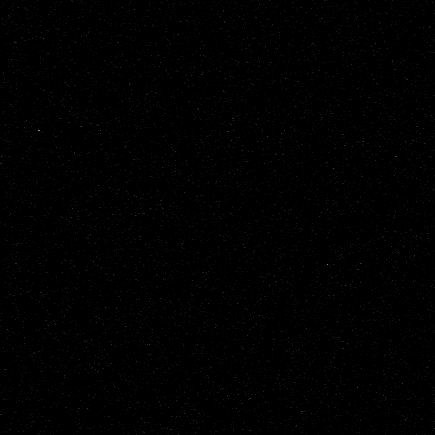

Supplement: Supplementary file 13 — Expanded View figures Source Data [file 44318_2026_747_MOESM13_ESM.zip › Figure_EV6/E/RGB/e1584_exp06_c322_mock_DE_3_W0002_P0001_T0001-1_ch02.tif]

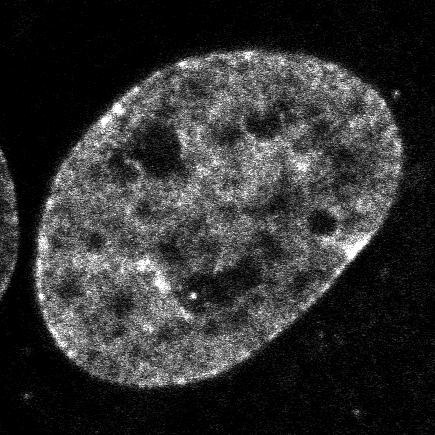

Supplement: Supplementary file 13 — Expanded View figures Source Data [file 44318_2026_747_MOESM13_ESM.zip › Figure_EV6/E/RGB/e1584_exp06_c322_mock_DE_3_W0002_P0001_T0001-1_ch01.tif]

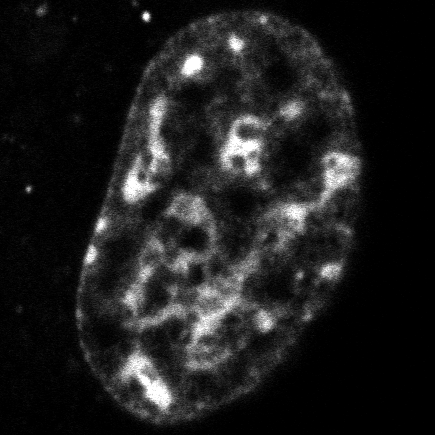

Supplement: Supplementary file 13 — Expanded View figures Source Data [file 44318_2026_747_MOESM13_ESM.zip › Figure_EV6/E/RGB/e1584_exp06_c322_p343_DE_3_W0001_P0001_T0001-1_ch01.tif]

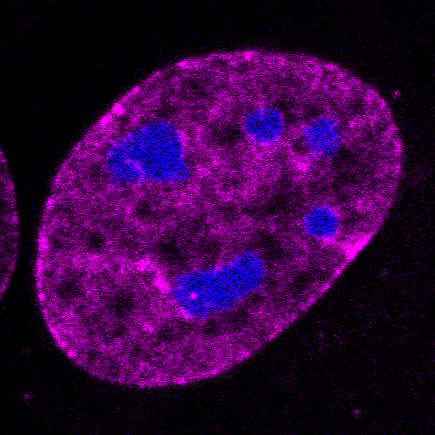

Supplement: Supplementary file 13 — Expanded View figures Source Data [file 44318_2026_747_MOESM13_ESM.zip › Figure_EV6/E/RGB/e1584_exp06_c322_mock_DE_3_W0002_P0001_T0001-1_NPM1_DNA.tif]

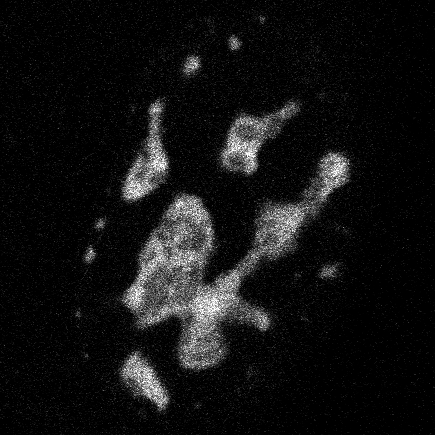

Supplement: Supplementary file 13 — Expanded View figures Source Data [file 44318_2026_747_MOESM13_ESM.zip › Figure_EV6/E/RGB/e1584_exp06_c322_p343_DE_3_W0001_P0001_T0001-1_ch02.tif]

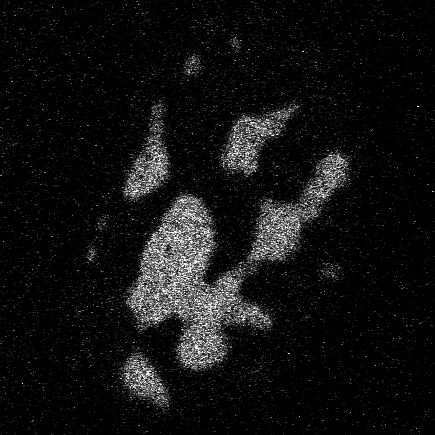

Supplement: Supplementary file 13 — Expanded View figures Source Data [file 44318_2026_747_MOESM13_ESM.zip › Figure_EV6/E/RGB/e1584_exp06_c322_p343_DE_3_W0001_P0001_T0001-1_ch03.tif]

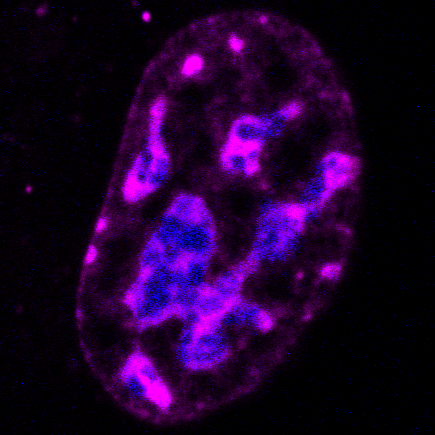

Supplement: Supplementary file 13 — Expanded View figures Source Data [file 44318_2026_747_MOESM13_ESM.zip › Figure_EV6/E/RGB/e1584_exp06_c322_p343_DE_3_W0001_P0001_T0001-1_NPM1_DNA.tif]

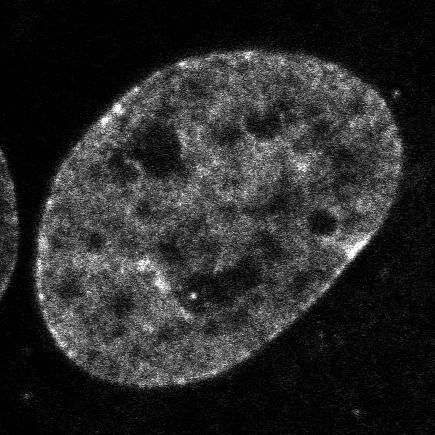

Supplement: Supplementary file 13 — Expanded View figures Source Data [file 44318_2026_747_MOESM13_ESM.zip › Figure_EV6/E/raw/e1584_exp06_c322_mock_DE_3_W0002_P0001_T0001-1.tif]

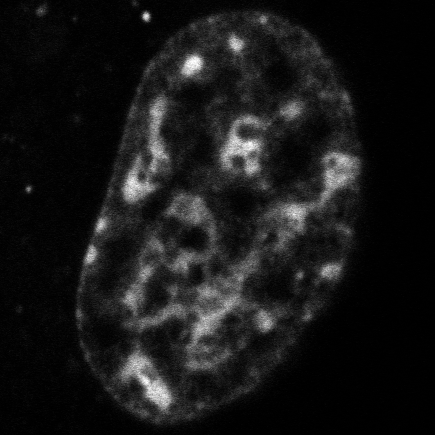

Supplement: Supplementary file 13 — Expanded View figures Source Data [file 44318_2026_747_MOESM13_ESM.zip › Figure_EV6/E/raw/e1584_exp06_c322_p343_DE_3_W0001_P0001_T0001-1.tif]

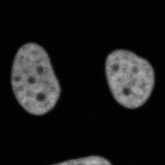

Supplement: Supplementary file 13 — Expanded View figures Source Data [file 44318_2026_747_MOESM13_ESM.zip › Figure_EV8/A/RGB/AR_W0059--s26948--DNTTIP2-1_ch02.tif]
