# Supplementary material for: Ki-67 shapes the nucleolus by anchoring chromatin via its amphiphilic properties
Source: EMBO J. 2026 Mar 24;45(9):3156–91. doi: 10.1038/s44318-026-00747-7 (PMC13144362; doi:10.1038/s44318-026-00747-7)
Supplement: Supplementary file 14 — Expanded View Figures [file 44318_2026_747_MOESM14_ESM.pdf]

Expanded View Figures

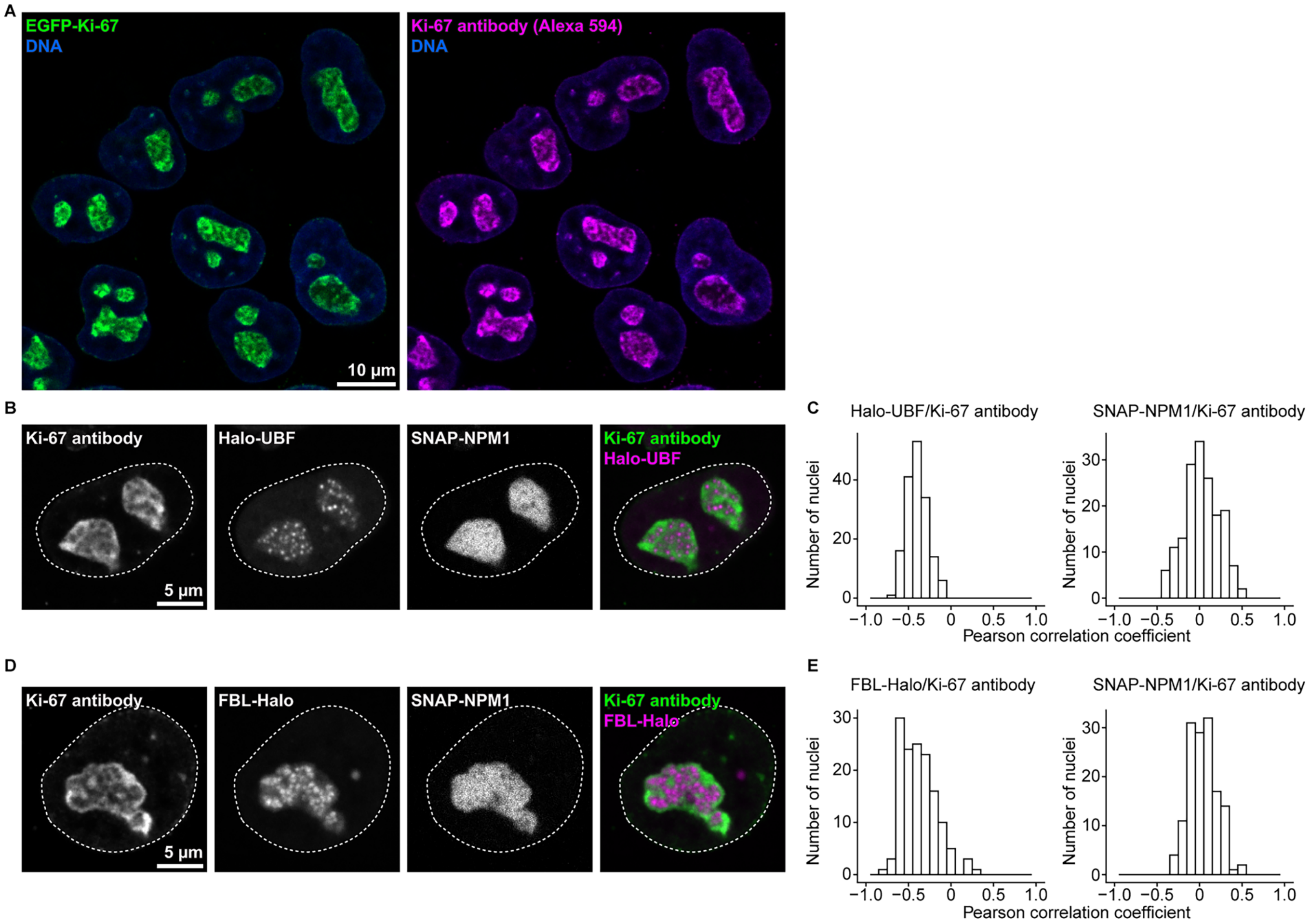

Figure EV1. Ki-67 displays a distinct localisation pattern in the nucleolus, separate from nucleolar subcompartments, related to Fig. 2.

(**A**) Immunofluorescence staining of Ki-67 in an endogenous EGFP-Ki-67 cell line. DNA was stained with DAPI. Notably, endogenous EGFP-Ki-67 signals substantially overlap with signals of anti-Ki-67 antibody. (**B**) Co-staining of Ki-67 and UBF. After labelling cells expressing SNAP-NPM1 and Halo-UBF with SNAP-SiR and Halo-TMR in live cells, immunofluorescence was performed for Ki-67 as in (**A**). Dashed lines indicate the nuclear boundary. (**C**) Colocalisation analysis between Ki-67 and UBF or NPM1 within the nucleolus. Pearson correlation coefficient between Ki-67 and UBF signals (left panel) or Ki-67 and NPM1 signals (right panel) was measured within nucleoli (segmented based on NPM1 signal). A Pearson correlation coefficient of +1.0 indicates a perfect positive linear correlation, −1.0 indicates a perfect negative correlation, and 0 indicates no correlation between the signals. (**D**) Co-staining of Ki-67 and FBL. After labelling cells expressing SNAP-NPM1 and FBL-Halo with SNAP-SiR and Halo-TMR in live cells, immunofluorescence was performed for Ki-67 as in (**A**). Dashed lines indicate the nuclear boundary. (**E**) Colocalisation analysis between Ki-67 and FBL or NPM1 within the nucleolus. Pearson correlation coefficient between Ki-67 and FBL signals (left panel) or Ki-67 and NPM1 signals (right panel) was measured within nucleoli, segmented based on NPM1 signal as in (**C**). For (**C**, **E**), $n = 144$ nucleoli and $n = 165$ nucleoli, respectively. 2 biological replicates. Source data are available online for this figure.

 © The Author(s)

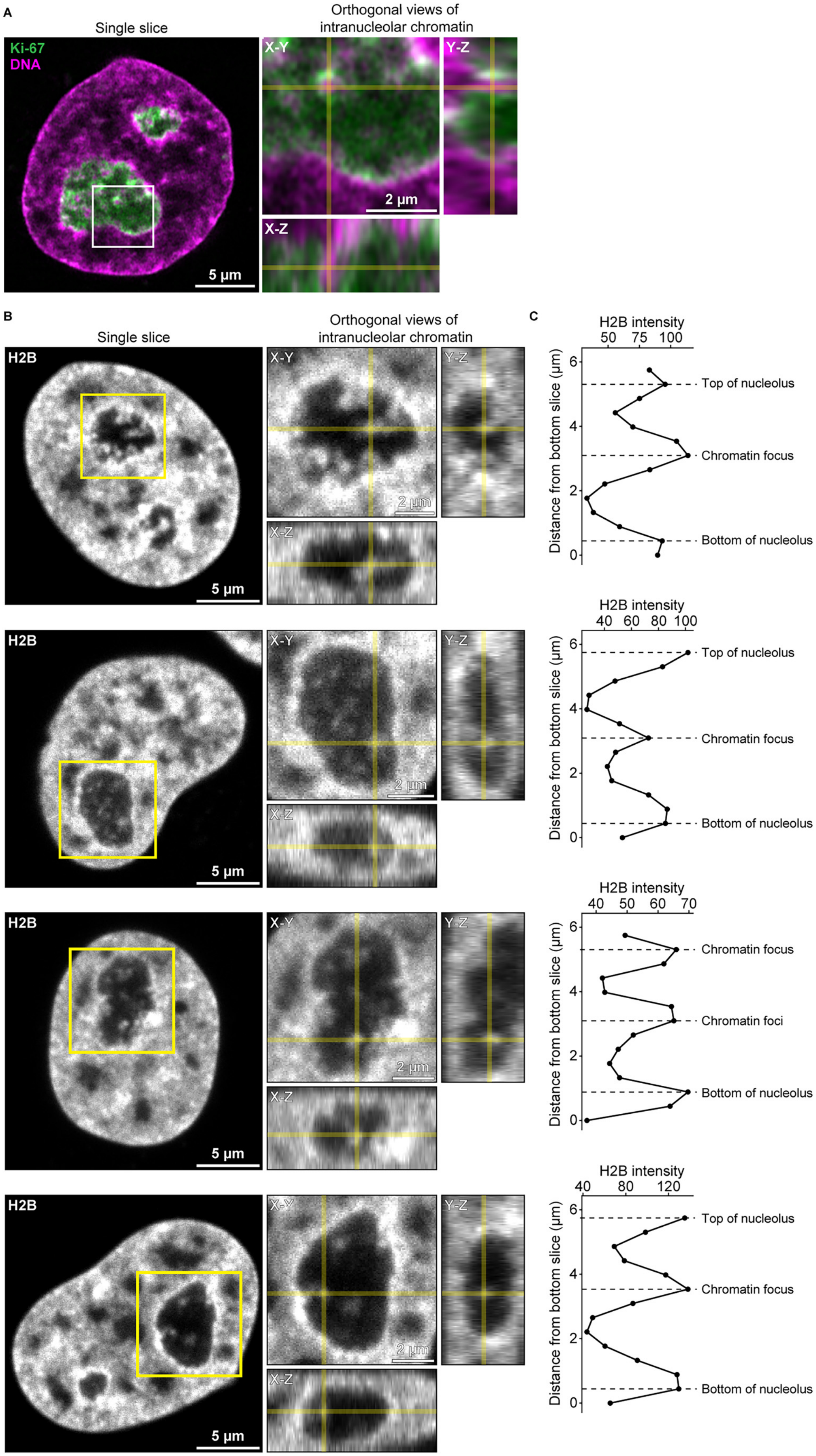
A
Single slice
Orthogonal views of intranucleolar chromatin
Ki-67
DNA
X-Y
Y-Z
X-Z
2 µm
5 µm
B
Single slice
Orthogonal views of intranucleolar chromatin
C
H2B
X-Y
Y-Z
X-Z
2 µm
5 µm
H2B intensity
Distance from bottom slice (µm)
Top of nucleolus
Chromatin focus
Bottom of nucleolus
Chromatin foci

Figure EV2. Chromatin invagination into the nucleolus, related to Fig. 2.

(**A**) 3D confocal stack of HeLa cells endogenously tagged with EGFP-Ki-67 and DNA labelled with SPY555-DNA from Fig. 2A,F. Insets show orthogonal views at the chromatin focus analysed in Fig. 2F. (**B**) 3D confocal stack of HeLa cells expressing H2B-mCherry. Insets show nucleolar chromatin foci, which orthogonal views confirm to arise from chromatin invaginations. A yellow cross marks a representative focus. (**C**) Chromatin signal along the *z* axis at the chromatin foci within the nucleolus. H2B intensity profile measured through the centre of the yellow cross in (**B**). Source data are available online for this figure.

 © The Author(s)

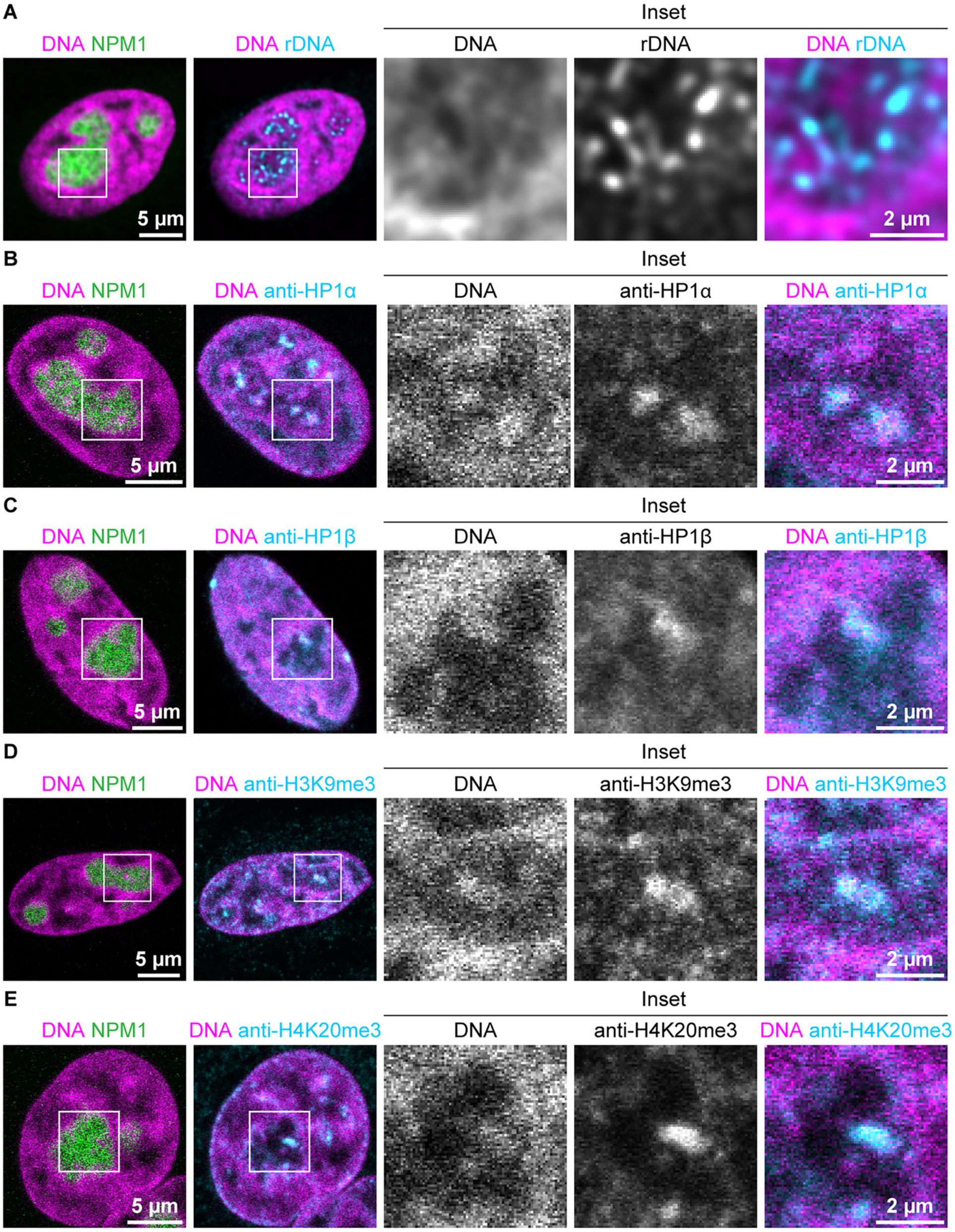

Figure EV3. Intranucleolar chromatin co-localises with heterochromatin markers, but not with rDNA, related to Fig. 2.

(**A**) Distinct localisation of rDNA and chromatin in the nucleolus. DNA-FISH against rDNA was performed in HeLa cells. DNA was stained with DAPI. (**B–E**) Colocalisation of intranucleolar chromatin with heterochromatin markers. Immunostaining for HP1α, HP1β, H3K9me3, and H4K20me3 was performed in HeLa cells. DNA was stained with DAPI. Two biological replicates were performed. Source data are available online for this figure.

© The Author(s)

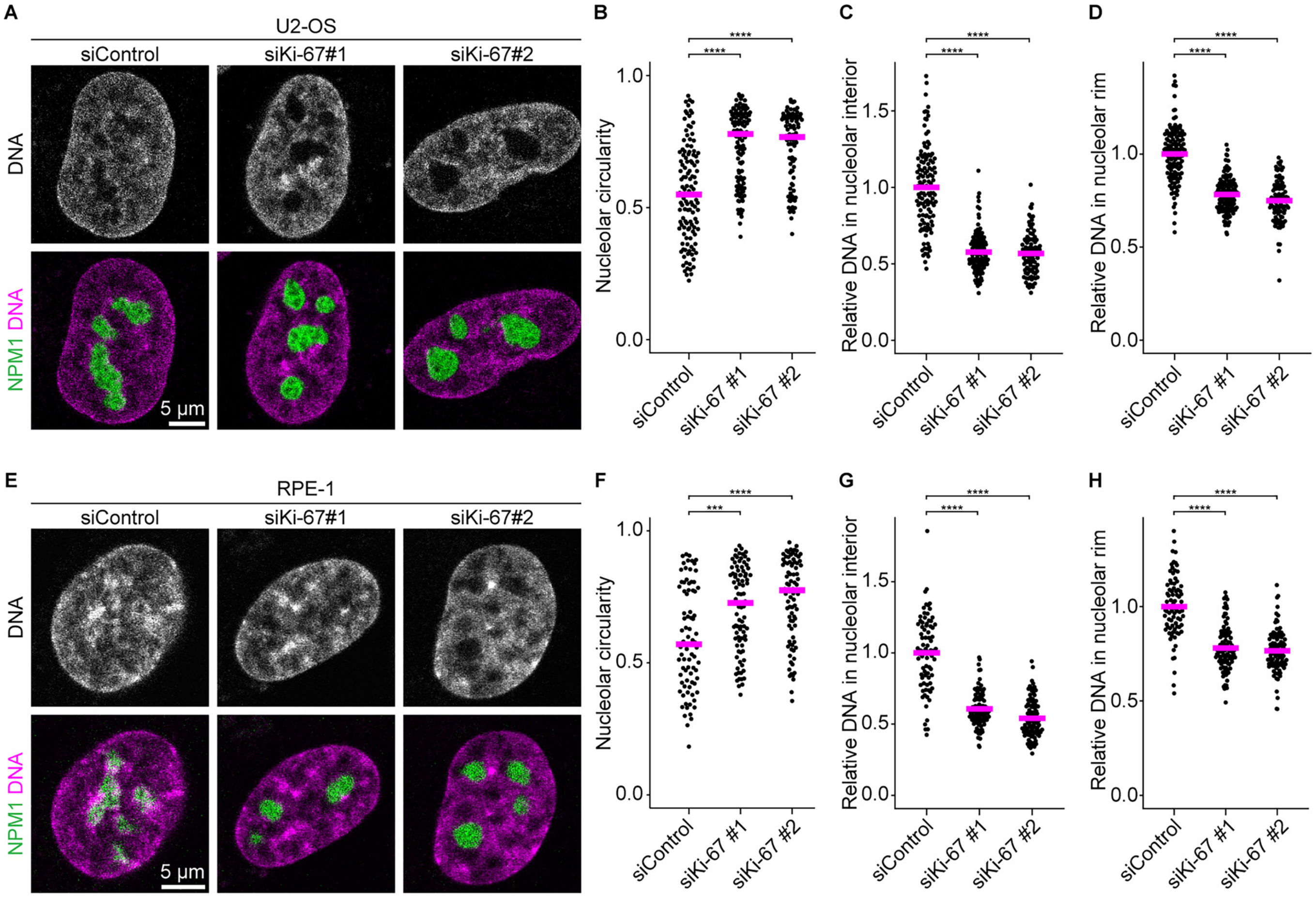

Figure EV4. Ki-67 depletion induces nucleolar rounding and chromatin removal from nucleoli in U2-OS and RPE-1 cells, related to Fig. 3.

(**A**) Live-cell imaging of U2-OS cells stably expressing EGFP-NPM1 following Ki-67 depletion. Cells were transfected with a non-targeting control siRNA (siControl) or two different Ki-67 siRNAs, followed by confocal imaging 48 h post-transfection. DNA was stained with SPY555-DNA before imaging. A single z-slice is shown. (**B**) Quantification of nucleolar roundness. Nucleoli were segmented based on NPM1 signals to measure their circularity. Circularity values of the largest nucleolus per nucleus are shown. Bars indicate median values. Statistical comparisons were performed against the siControl sample: siKi-67 #1, $P = 4.69 \times 10^{-13}$; siKi-67 #2, $P = 8.99 \times 10^{-11}$. (**C**, **D**) Quantification of chromatin enrichment in the nucleolar interior and its rim. Relative DNA signal intensities were calculated as Fig. 3C,D for the nucleolar interior (**C**) or the nucleolar rim (**D**). Bars indicate mean values. Statistical comparisons were performed against the siControl sample: For (**C**), siKi-67 #1, $P = 7.73 \times 10^{-29}$; siKi-67 #2, $P = 7.73 \times 10^{-29}$. For (**D**), siKi-67 #1, $P = 1.50 \times 10^{-23}$; siKi-67 #2, $P = 8.99 \times 10^{-30}$. (**E**) Live-cell imaging of RPE-1 cells stably expressing SNAP-NPM1 following Ki-67 depletion. Cells were transfected with a non-targeting control siRNA (siControl) or two different Ki-67 siRNAs, followed by confocal imaging 48 h post-transfection. SNAP-NPM1 and DNA were stained with SiR-SNAP and SPY555-DNA before imaging. A single z-slice is shown. (**F**) Quantification of nucleolar roundness. Nucleoli were segmented based on NPM1 signals to measure their circularity. Circularity values of the largest nucleolus per nucleus are shown. Bars indicate median values. Statistical comparisons were performed against the siControl sample: siKi-67 #1, $P = 2.32 \times 10^{-4}$; siKi-67 #2, $P = 1.08 \times 10^{-6}$. (**G**, **H**) Quantification of chromatin enrichment in the nucleolar interior and its rim. Relative DNA signal intensities were calculated as Fig. 3C,D for the nucleolar interior (**G**) or the nucleolar rim (H). Bars indicate mean values. Statistical comparisons were performed against the siControl sample: For (**G**), siKi-67 #1, $P = 7.35 \times 10^{-16}$; siKi-67 #2, $P = 8.10 \times 10^{-26}$. For (**H**), siKi-67 #1, $P = 6.11 \times 10^{-16}$; siKi-67 #2, $P = 4.30 \times 10^{-18}$. For (**B–D**), $n = 122$ nuclei (siControl), 116 nuclei (siKi-67#1), 92 nuclei (siKi-67#2), 2 biological replicates. For (**F–H**), $n = 82$ nuclei (siControl), 90 nuclei (siKi-67#1), 88 nuclei (siKi-67#2), 2 biological replicates. Statistical tests were performed with the Kruskal–Wallis test followed by Dunn's test, ns (not significant) $P > 0.05$, ***$P < 0.001$, ****$P < 0.0001$. Source data are available online for this figure.

 © The Author(s)

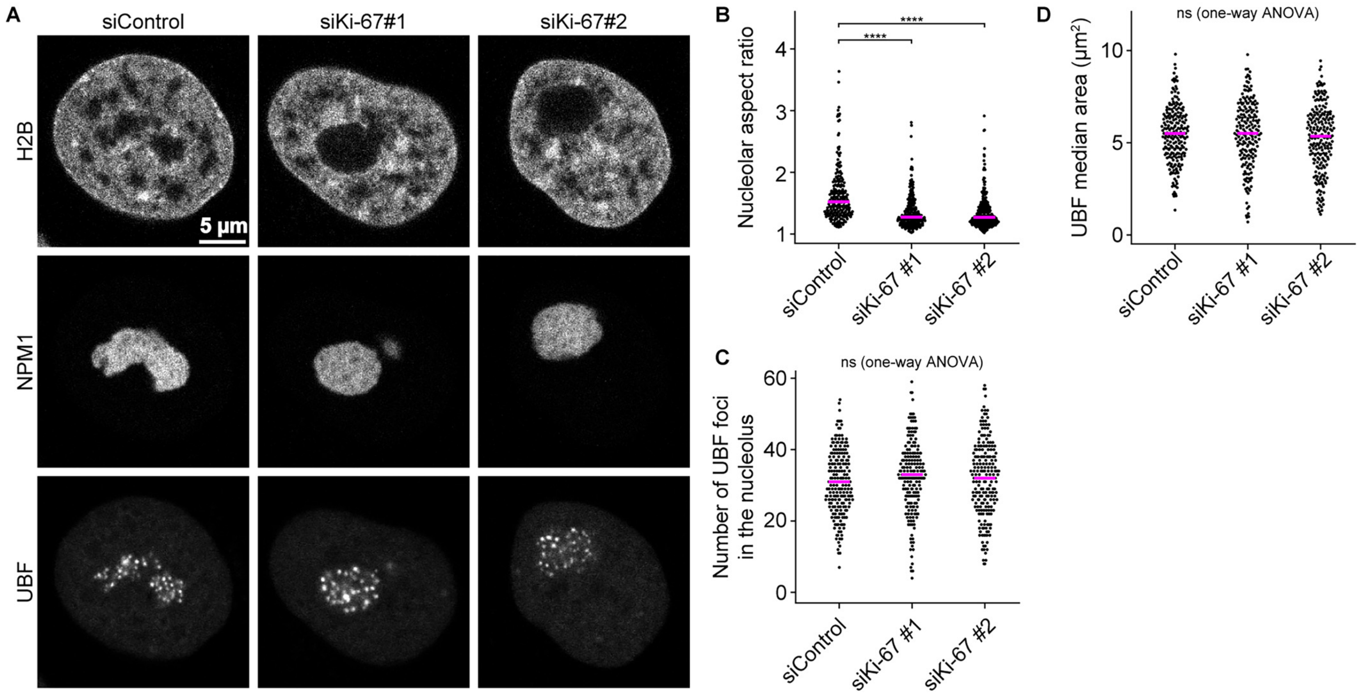

Figure EV5. Ki-67 depletion does not disrupt internal nucleolar subcompartments, related to Fig. 3.

(**A**) Effects of Ki-67 depletion on nucleolar subcompartments. HeLa cells expressing endogenously tagged Halo-UBF and stably overexpressing SNAP-NPM1 and H2B-mNeonGreen were transfected with a non-targeting control siRNA (siControl) or Ki-67 siRNAs. After 72 h of transfection, Halo-UBF and SNAP-SNAP were labelled with Halo-TMR and SNAP-SiR, respectively. Images were acquired using spinning disk microscopy. (**B**) Quantification of the nucleolar shape. Nucleoli were segmented based on NPM1 signals, and their aspect ratio was measured. Bars indicate the median. Statistical comparisons were performed against the siControl sample: siKi-67 #1, $P = 4.16 \times 10^{-16}$; siKi-67 #2, $P = 6.91 \times 10^{-17}$. Statistical tests were performed with the Kruskal–Wallis test followed by Dunn's test, $^{****}P < 0.0001$. (**C**, **D**) Quantification of the size and number of internal UBF subcompartments based on segmented UBF signals. Median area per the nucleus and the total number of subcompartments were measured. Bars indicate median values. No significant differences were detected among samples (one-way ANOVA, $P = 0.28$). For (**B–D**), $n = 207$ nuclei (siControl), 194 nuclei (siKi-67#1), 214 nuclei (siKi-67#2), 2 biological replicates. Source data are available online for this figure.

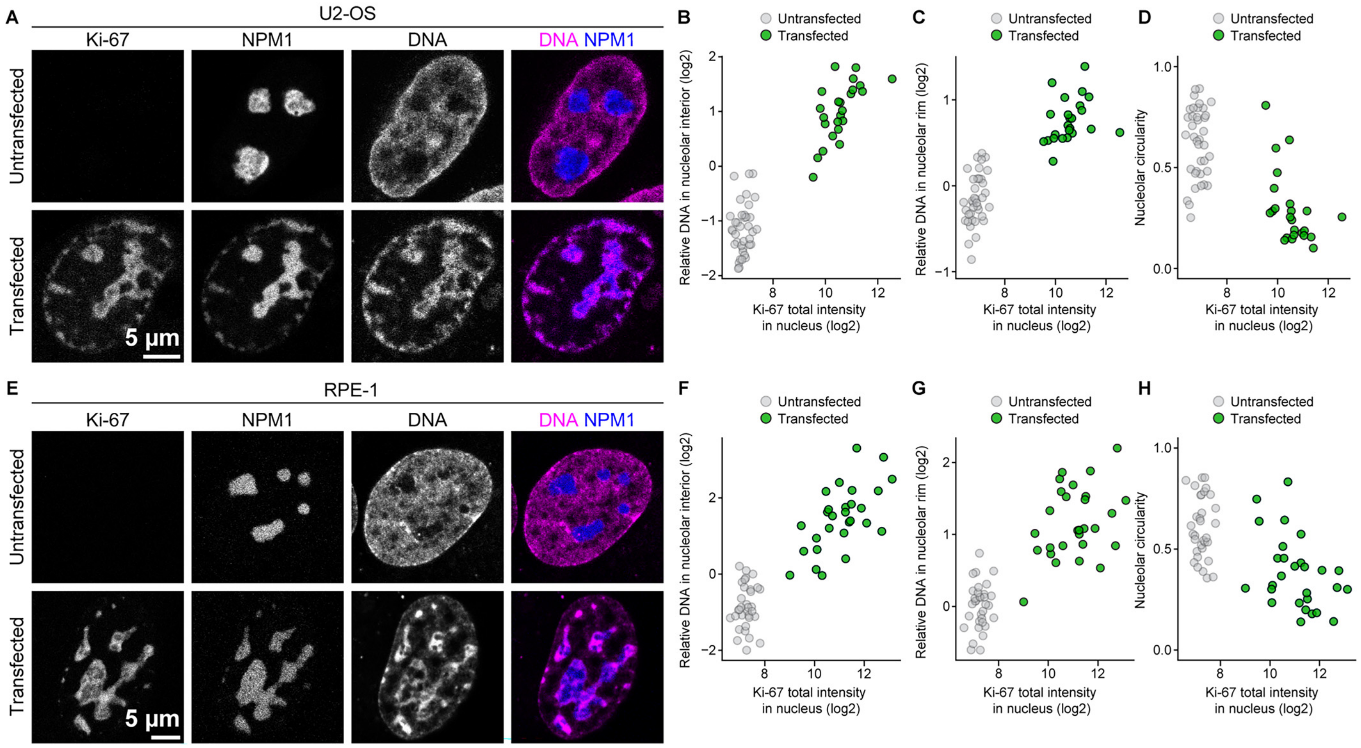

Figure EV6. Ki-67 overexpression induces irregularly shaped nucleoli and excess chromatin loading into nucleoli in U2-OS and RPE-1 cells, related to Fig. 4.

(**A**) Live-cell imaging of Ki-67-overexpressing cells in U2-OS cells. Cells stably expressing EGFP-NPM1 were transfected with a Halo-Ki-67 plasmid. Halo-Ki-67 was labelled with Halo-JF646, and DNA was stained with SPY555-DNA. (**B**, **C**) Ki-67 expression level-dependent chromatin enrichment in the nucleolar interior (**B**) and rim (**C**). Relative DNA signal intensities, calculated as described in Fig. 3D,E, are plotted against total nuclear Halo-Ki-67 intensity. (**D**) Ki-67 expression level-dependent increase in the irregularity of nucleolar shape. Median circularity of segmented nucleoli from NPM1 signals per nucleus is plotted against the total intensity of Halo-Ki-67 in the nucleus. (**E**) Live-cell imaging of Ki-67-overexpressing cells in RPE-1 cells. Cells stably expressing SNAP-NPM1 were transfected with an EGFP-Ki-67 plasmid. DNA was stained with SPY555-DNA. (**F**, **G**) Ki-67 expression level-dependent chromatin enrichment in the nucleolar interior (**F**) and its rim (**G**). Relative DNA signal intensities, calculated as described in Fig. 3D,E, are plotted against total nuclear EGFP-Ki-67 intensity. (**H**) Ki-67 expression level-dependent increase in the irregularity of nucleolar shape. Median circularity of segmented nucleoli from NPM1 signals per nucleus is plotted against the total intensity of EGFP-Ki-67 in the nucleus. For (**B**–**D**), $n = 38$ nuclei (Untransfected); $n = 24$ nuclei (Transfected), 2 biological replicates. For (**F**–**H**), $n = 33$ nuclei (Untransfected); $n = 28$ nuclei (Transfected), 3 biological replicates. Source data are available online for this figure.

 © The Author(s)

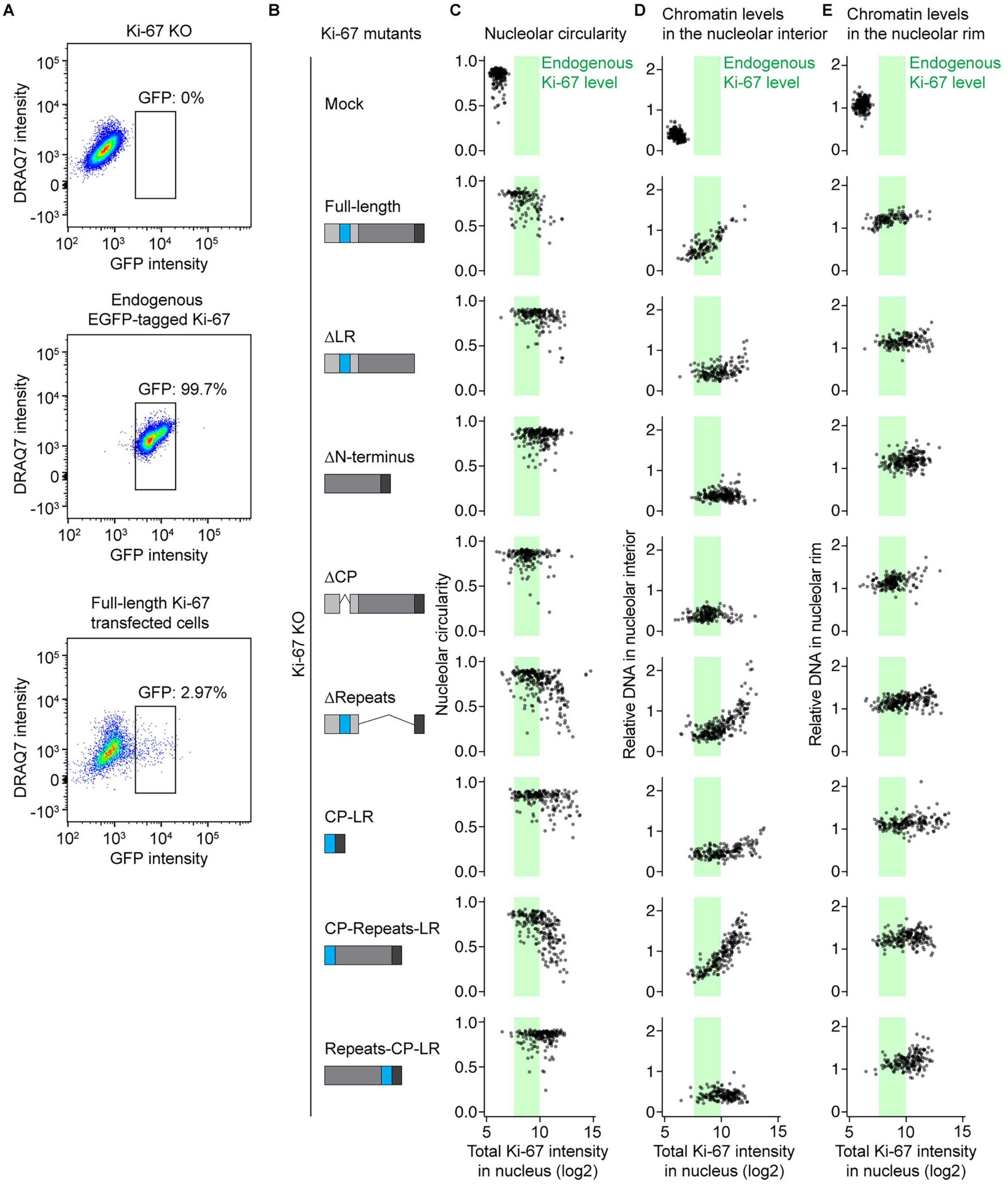
A
Ki-67 KO
GFP: 0%
DRAQ7 intensity
GFP intensity
Endogenous
EGFP-tagged Ki-67
GFP: 99.7%
Full-length Ki-67
transfected cells
GFP: 2.97%
B
Ki-67 mutants
Ki-67 KO
Mock
Full-length
ΔLR
ΔN-terminus
ΔCP
ΔRepeats
CP-LR
CP-Repeats-LR
Repeats-CP-LR
C
Nucleolar circularity
Endogenous
Ki-67 level
D
Chromatin levels
in the nucleolar interior
Relative DNA in nucleolar interior
E
Chromatin levels
in the nucleolar rim
Relative DNA in nucleolar rim
Total Ki-67 intensity
in nucleus (log2)

Figure EV7. The effect of Ki-67 mutant expression levels on nucleolar shape and chromatin levels within nucleoli and their rim, related to Fig. 6.

(**A**) Isolation of cells transfected with Ki-67 mutants at the endogenous Ki-67 levels by FACS. Ki-67 KO cells (left) and endogenously EGFP-Ki-67-expressing cells (middle) were used as a reference for FACS sorting. (**B**) Schematic of Ki-67 domain mutants. (**C**) Ki-67 expression level-dependent increase in the nucleolar irregularity. Median circularity of nucleoli based on NPM1 segmentation per nucleus is plotted against the total intensity of EGFP-Ki-67 in the nucleus. Green rectangles show the wild-type Ki-67 expression range determined by imaging of endogenously tagged EGFP-Ki-67 cells. (**D**, **E**) Ki-67 expression level-dependent chromatin enrichment in the nucleolar interior (**D**) and rim (**E**). Relative DNA signal intensities, calculated as described in Fig. 3D,E, are plotted against total EGFP-Ki-67 intensity in the nucleus. Green rectangles show wild-type Ki-67 expression. For (**C–E**), $n = 220$ nuclei (mock), $n = 126$ nuclei (full-length), $n = 162$ nuclei (ΔLR), $n = 228$ nuclei (ΔN-terminus), $n = 181$ nuclei (ΔCP), $n = 128$ nuclei (LR), $n = 250$ nuclei (ΔRepeats), $n = 189$ nuclei (CP-LR), $n = 212$ nuclei (CP-Repeats-LR), $n = 183$ nuclei (Repeats-CP-LR), 3 biological replicates. Source data are available online for this figure.

© The Author(s)

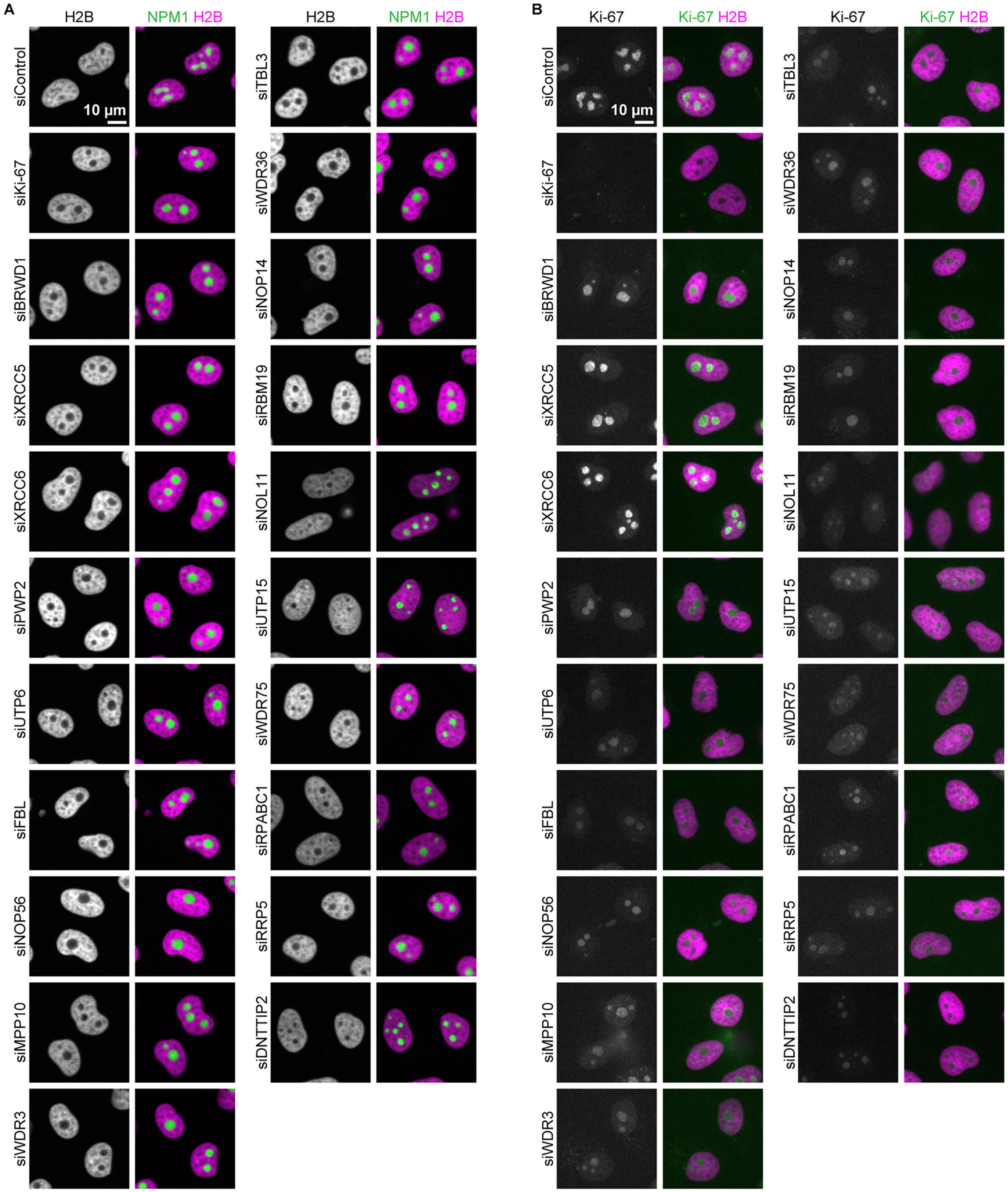
A
H2B
NPM1 H2B
H2B
NPM1 H2B
siControl
10 µm
siKi-67
siBRWD1
siXRCC5
siXRCC6
siPWP2
siUTP6
siFBL
siNOP56
siMPP10
siWDR3
siTBL3
siWDR36
siNOP14
siRBM19
siNOL11
siUTP15
siWDR75
siRPABC1
siRRP5
siDNTTIP2
B
Ki-67
Ki-67 H2B
Ki-67
Ki-67 H2B
siControl
10 µm
siKi-67
siBRWD1
siXRCC5
siXRCC6
siPWP2
siUTP6
siFBL
siNOP56
siMPP10
siWDR3
siTBL3
siWDR36
siNOP14
siRBM19
siNOL11
siUTP15
siWDR75
siRPABC1
siRRP5
siDNTTIP2

© The Author(s)

Figure EV8. Chromatin enrichment and Ki-67 expression in cells upon siRNA depletion of the top 20 candidate genes, inducing nucleolar rounding, related to Fig. 8.

(**A**) Live-cell imaging of chromatin enrichment in the nucleolus following depletion of the top 20 candidate proteins. Cells expressing NPM1-EGFP and H2B-mCherry were transfected with a non-targeting control siRNA (siControl) or siRNAs targeting the top 20 candidate genes (Fig. 1B). Images were acquired 72 h after siRNA transfection. (**B**) Live-cell imaging of endogenous EGFP-Ki-67 following depletion of the top 20 candidate proteins. Cells endogenously tagged EGFP-Ki-67 and stably expressing SNAP-NPM1 and H2B-mCherry were transfected with a siControl or siRNAs targeting the top 20 candidate genes (Fig. 1B). Images were acquired 72 h after siRNA transfection. Source data are available online for this figure.

 © The Author(s)
